# The efficacy of Euler diagrams and linear diagrams for visualizing set cardinality using proportions and numbers

**DOI:** 10.1371/journal.pone.0211234

**Published:** 2019-03-28

**Authors:** Gem Stapleton, Peter Chapman, Peter Rodgers, Anestis Touloumis, Andrew Blake, Aidan Delaney

**Affiliations:** 1 Centre for Secure, Intelligent and Usablity Systems, School of Computing, Engineering and Mathematics, University of Brighton, Brighton, United Kingdom; 2 School of Computing, Edinburgh Napier University, Edinbugh, United Kingdom; 3 School of Computing, University of Kent, Canterbury, United Kingdom; 4 Bloomberg, London, United Kingdom; University of Toronto, CANADA

## Abstract

This paper presents the first empirical investigation that compares Euler and linear diagrams when they are used to represent set cardinality. A common approach is to use area-proportional Euler diagrams but linear diagrams can exploit length-proportional straight-lines for the same purpose. Another common approach is to use numerical annotations. We first conducted two empirical studies, one on Euler diagrams and the other on linear diagrams. These suggest that area-proportional Euler diagrams with numerical annotations and length-proportional linear diagrams without numerical annotations support significantly better task performance. We then conducted a third study to investigate which of these two notations should be used in practice. This suggests that area-proportional Euler diagrams with numerical annotations most effectively supports task performance and so should be used to visualize set cardinalities. However, these studies focused on data that can be visualized reasonably accurately using circles and the results should be taken as valid within that context. Future work needs to determine whether the results generalize both to when circles cannot be used and for other ways of encoding cardinality information.

## Introduction

This paper sets out to shed light on how to represent sets and their cardinalities in a manner most effective for users. This is of particular significance because there are enormous amounts of set-based data available in a wide variety of application areas [2]. Set visualization techniques often exploit closed curves (or variations thereof) [9, 11, 25, 32, 36] or lines [1, 8, 15, 34]. This paper therefore focuses on such methods by evaluating extensions of Euler diagrams (closed curves) [39, 41] and linear diagrams (lines) [34] that represent cardinality information. In addition, we consider two common ways of representing cardinality: proportions (of areas or lengths) and numerical annotations.

An example of set cardinality can be seen with news providers that typically allow users to subscribe and receive dedicated feeds for multiple interest areas, such as “Politics”, “Sport” and “Business”. Each area of interest defines a set over those who subscribe to it. The cardinality of each set is defined by the number of subscribers. More detail of the size of the set intersections may be useful for analysis. For instance, one such intersection is those subscribers to both “Politics” and “Sport” but not any other area, the intersection cardinality being the number of such subscribers.

Set-based data has long been the focus of the information visualization community, not least because of its extensive occurrence in numerous settings. One such setting is the biomedical domain, where Euler diagrams have gained particular prominence as a visualization technique, along with social network visualization and many other areas; see [33] for a survey of Euler diagrams. Example visualization tools include BioVenn [19], Euler3 [35], eulerAPE [27], VennMaster [20] and venneuler [41]. BioVenn and eulerAPE create area-proportional Venn diagrams (which are a special case of Euler diagrams) but both are limited to three sets, as is Euler3. VennMaster and venneuler are more sophisticated, utilizing area-proportional Euler diagrams to visualize an arbitrary number of sets, although the diagrams become increasingly cluttered as the number of sets increase. Ten sets has been suggested as a limit on effective Euler diagrams [2], but this is subjective and depends on the complexity of the relationships between the sets.

These techniques have quite different layout and graphical features. As well as using colour in different ways to each other, BioVenn and venneuler use circles, eulerAPE uses ellipses, VennMaster uses regular polygons, and Euler3 uses both circles and polygons. The circles, ellipses and polygons, as well as the regions they form, are intended to be proportional to the cardinalities of the represented sets and their intersections. In addition, circles have been shown to effectively support cognition when using Euler diagrams [5].

However, no area-proportional Euler diagram technique can achieve completely accurate proportions when using convex shapes: it is possible that non-empty set intersections are omitted or that they have may have incorrect areas. The venneuler technique has been theoretically shown to achieve superior visualizations—in the sense of better approximating the region areas—than VennMaster [41]. Thus, in terms of its generality (not being restricted to three sets), superior area accuracy, and use of (cognitively effective) circles we regard venneuler as the best practical layout method for area-proportional Euler diagrams.

**Fig 1:**
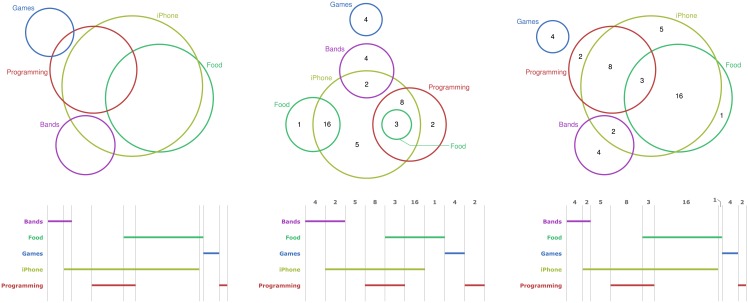
**Representing cardinalities using proportions (left), numerical annotations (middle), and both (right); top**: Euler, bottom: Linear.

Given the approximate nature of the layouts currently available using automated tools, it is unsurprising that Euler diagrams are sometimes augmented with numbers placed in the regions to represent cardinality, as shown in Fig 1; for examples of numerical annotations in area-proportional Euler diagrams see [19, 27] and for non-area-proportional Euler diagrams with numerical annotations see [3, 17]. The key message here is that there are three common methods employed when visualizing sets and their cardinalities: area-proportions, numerical annotations, or their combination. With high interest in these three methods and their growing exploitation for visualizing sets and their cardinalities, it is important to understand their relative cognitive benefits. However, at present there is no empirical insight into which of these methods is cognitively superior when performing set-theoretic tasks that rely on cardinality information—we provide such insight in the section entitled *Euler Diagrams: Results*.

Linear diagrams, on the other hand, are a relative newcomer to the scene of automated set visualization. They represent each set by a (possibly broken) line drawn horizontally. The set intersections are represented by columns, called overlaps. A set-line appears in an overlap if and only if the elements in the corresponding set intersection are in the set. For example, the first overlap of the linear diagrams in Fig 1 shows that there are elements in Bands only (i.e. $\text{Bands}\cap \bar{\text{Food}}\cap \bar{\text{Games}}\cap \bar{\text{iPhone}}\cap \bar{\text{Programming}}$). We note that, whilst techniques to minimize line breaks have been developed, some set lines are neccessarily broken to correctly display many data sets. Originally developed by Leibniz [12], linear diagrams have recently begun to gain prominence, with empirical studies providing insight into the cognitive effects of graphical and topological choices made when laying them out [34]. Even before we knew how to produce effective layouts, it was already shown that people find it easier to construct linear diagrams compared to Euler diagrams [15]. They also bring significant cognitive benefits over Euler diagrams when people complete set-theoretic tasks that require diagram interpretation [6]. However, these prior works did not consider the representation of cardinality information and it’s extraction by people performing tasks.

As well as having cognitive advantages over Euler diagrams, linear diagrams readily lend themselves to visualizing cardinality information: linear diagrams can *always* accurately visualize sets and their cardinalities by exploiting length-proportions, making them superior to Euler diagrams from this perspective. Theoretically, area-proportional Euler diagrams do exist for any finite collection of sets [38] yet an implemented method does not exist. Drawing them by hand is extremely difficult, if not impossible.

The theoretical and cognitive benefits of linear diagrams suggests that the time is right for them to be more widely investigated. In particular, it is natural to ask whether, when set cardinalities are considered, linear diagrams remain superior visualizations—we provide such insight in this paper (see RQ3 below), after evaluating the use of length proportions, numerical annotations and their combination (see RQs 1 and 2).

Apart from the aforementioned prior studies on Euler diagrams and linear diagrams, there have been studies relating to set size in the context of Bayesian reasoning [29, 26]. Although concerned with set size, these studies deal with less general diagrams, as both have a fixed number of sets and no flexibility in set overlap.

Fig 1 shows different ways of depicting set cardinalities. The leftmost diagrams use proportions: the set iPhone has the largest cardinality because the iPhone circle has the largest circle-area and the iPhone line has the longest line-length. The middle diagrams use numerical annotations: the largest set intersection is Food and iPhone because the region inside both the Food and iPhone circles and, in the linear diagram, the Food and iPhone overlap are both annotated with 16 which is the largest number. The rightmost diagrams use both proportions and numerical annotations.

The aim of this paper is to establish which of the three methods—proportions, numerical annotations or both—is most effective for visualizing set cardinalities when using Euler and linear diagrams. An overarching goal is to ensure practical impact of the research, so we evaluated selected state-of-the-art visualization methods that exist for data of this kind: venneuler [41], iCircles [37], and the linear diagram generator in [34]. Restricting ourselves to data that can be visualized with the selected methods, we answer the following research questions by conducting three empirical studies that measured participants’ performance:

**RQ1:** Are area- and length-proportional diagrams enhanced by the inclusion of numerical annotations reflecting actual set cardinality?
**RQ2:** Is a numerical annotation alone, without proportions, more effective?
**RQ3:** Which technique is the most effective overall and, thus, should be used in practice?

The methods used for our empirical studies are in the section entitled *Methods*. Study 1, which focused on the three Euler diagram variants, is covered in the section entitled *Euler Diagrams: Results* and study 2, on the three linear diagram variants, is covered in the section entitled *Linear Diagrams: Results*. These studies suggested that area-proportional Euler diagrams with numerical annotations and length-proportional linear diagrams were most effective. These two techniques were subsequently evaluated against each other, the results of which are in the section entitled *Comparing Euler and Linear Diagrams*. This third study revealed that area-proportional Euler diagrams with numerical annotations were most effective overall. Threats to validity are covered in the penultimate section, after which we conclude. All the diagrams and questions used in our study, the data collected, and statistical output are included in the **submitted supplementary material**. The experimental software can be accessed via the study web page: http://www.eulerdiagrams.org/cardinality/.

## Methods

To address RQs 1-3, three between-group empirical studies were conducted that measured task performance in terms of accuracy and time. This section describes the studies’ design. Ethical approval was obtained from the University of Kent (approval number 0811516).

### Data for visualization

We obtained real-world data from the SNAP data set collection [21]. SNAP contains *ego networks* of 1000 Twitter users and sets comprise users that subscribe to particular lists. Ego networks have a so-called focal node, the ‘ego’ which in the case of Twitter is a user, along with the nodes to whom the ego node is directly connected and any incident edges. In turn, these connected nodes can be viewed as ‘ego’ nodes and may themselves be connected to other nodes, thus forming a social network. We chose SNAP data sets that included a sufficient number of sets to ensure the diagrams generated from them were non-trivial (i.e. would require cognitive effort to interpret). Based on previous experience, five sets were deemed sufficient to require cognitive effort and seven sets were not expected to hinder task performance. As such, data sets with either five or seven sets were selected to give controlled variety in diagram complexity. Previous evaluations of techniques for visualizing sets have used similar or smaller numbers of sets. Kelpfusion was evaluated using four or five sets, with the latter being described as “hard” in terms of difficulty level for participants [25]. An evaluation of LineSets used three, four, or five sets which were described as “easy”, “medium” and “hard” in terms of level of difficulty [1]. In both cases, these data sets were used to generate diagrams for task-based empirical evaluations like that in this paper. Therefore, in terms of number of sets, our evaluation can be seen as increasing the difficultly level of the visualization used in the study as compared to prior work.

We used the venneuler technique to generate area-proportional Euler diagrams as we regard it to be the best available method. Venneuler produces exact areas for circles but, therefore, necessarily *approximate* areas for regions representing set intersections and sometimes *omits regions* that should have non-zero area. This problem impacts our choice of data sets; it does not arise with linear diagrams. We chose 18 five-set data sets and 18 seven- or eight-set data sets with the largest number of intersections that could be visualized with venneuler; a single set was removed from the eight-set data sets. For each number of sets, two data set were used for training participants, the rest were used for the main study.

Table 1 provides insight into the five-set data sets. Rows 2 to 6 provide information on the set intersections present, rows 7 to 9 provide information on the number of data items, and row 10 indicates the allocated task category (explained in the section entitled *Tasks and Training*). So, for data set 1, row 1 tells us that there are four 1-set intersections; these 1-set intersections arise when there are data elements that appear in exactly one of the five sets. Likewise 2-set intersections arise when data elements appear in exactly two sets, and so forth. Rows 7 and 8 indicate, respectively, that the cardinalities of the five sets range from 2 to 16 and the set intersections range from 1 to 6. Table 2 is for the seven-set data sets.

**Table 1:** Complexity of five-sets data.

Data Set	1	2	3	4	5	6	7	8	9	10	11	12	13	14	15	16
1-set intersections	4	5	5	5	4	4	5	5	5	5	5	4	4	4	5	5
2-set intersections	3	3	4	4	3	5	5	5	5	5	5	5	6	5	6	4
3-set intersections	2	1	1	1	3	1		1	1	2	2	2	2	3	4	
4-set intersections												1		1		
Total intersections	9	9	10	10	10	10	10	11	11	12	12	12	12	13	15	9
Card. range sets	2–16	4–34	5–21	3-15	3–54	7–28	11-32	10-30	4-44	11-40	13–119	9–79	11-90	23–43	14–84	1–20
Card. range int.	1–6	1–16	1–14	2–6	2–18	1–10	3-22	2–17	1–10	2–20	2–81	1–42	2–40	2–16	2–16	2–10
Total items	28	45	54	32	64	44	87	59	54	84	155	103	111	74	116	45
Task Type	S>	S<	I>	I<	S>	S<	I>	I<	S>	S<	I>	I<	S>	S<	I>	I<

**Table 2:** Complexity of seven-sets data.

Data Set	17	18	19	20	21	22	23	24	25	26	27	28	29	30	31	32
1-set intersections	7	5	4	7	7	7	5	6	7	7	7	7	7	4	7	7
2-set intersections		2	1	1	2	4	10	2	2	1	3	6	4	2	4	2
3-set intersections					2		1		1		2		1	4		2
4-set intersections														2		
≥ 5-set intersections														1 × 5, 1 × 6		
Total intersections	7	8	5	8	11	11	16	8	10	8	12	13	12	14	11	11
Card. range sets	1–4	1–17	1–20	1–13	5–55	3–25	4–47	4–28	1–39	2–53	10–39	1–105	8–57	11-149	7–33	3–55
Card. range int.	1–4	1–15	1–20	1–11	1–33	1–21	1–14	4–16	1–24	2–53	1–33	1–61	2–57	1–98	2–25	1–33
Total items	12	32	19	35	88	60	62	64	70	93	120	138	167	176	81	86
Task Type	S>	S<	I>	I<	S>	S<	I>	I<	S>	S<	I>	I<	S>	S<	I>	I<

**Fig 2:**
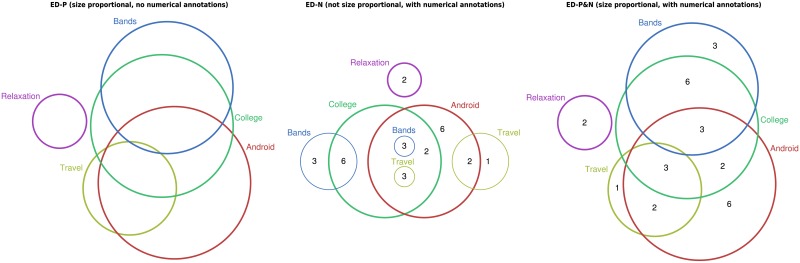
Euler diagrams, data set 1. (from left: ED-P, ED-N, ED-P&N).

**Fig 3:**
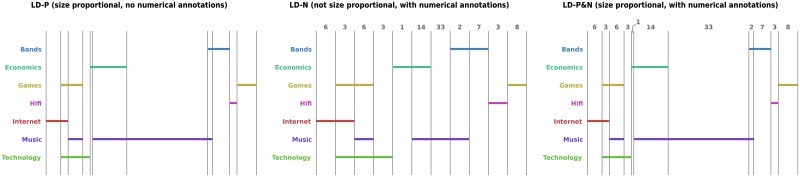
Linear diagrams, data set 32. (from left: LD-P, LD-N, LD-P&N).

As participants may not be familiar with ego networks, the questions they were asked (details in the section entitled *Tasks and Training*) made no reference to Twitter. In addition, to avoid any possibility of previous knowledge affecting the results, all set names were changed. We still used a real-world scenario—the interests of people—to make the tasks accessible. The sets’ names were chosen so that no two sets in any one diagram started with the same letter; this was to reduce the potential for misreading set names. Scaled versions of the Euler and, respectively, linear diagrams generated from data sets 1 and 32 can be seen in Figs 2 and 3. The abbreviations in that figure (and throughout) are: -P (size proportional, with no numerical annotations); -N (not size proportional, with numerical annotations); and -P&N (size proportional, with numerical annotations).

### Creating diagrams for the studies

The 32 data sets chosen for the study were used to create Euler and linear diagrams reflecting the three treatments to be applied. We used selected state-of-the-art layout tools to create the diagrams, ensuring that the results had practical utility, namely: venneuler [41], iCircles [37] and the linear diagram generation tool described in [34].

As identified in the background section, venneuler is currently the most effective tool for generating area-proportional Euler diagrams: it can visualize arbitrarily many sets and does so in a demonstrably more accurate manner than VennMaster. Therefore, we used venneuler to generate the 32 area-proportional Euler diagrams. These diagrams were then manually augmented with numbers to indicate the cardinality of set intersections, creating diagrams for the second treatment group. See the left and right diagrams in Figs 1 and 2 for examples.

We also needed a method to generate non-area-proportional Euler diagrams. Whilst non-area-proportionality necessarily means that there will be layout variations across treatments, we opted to chose a technique that produced layouts with similar graphical features to venneuler to reduce such variations. In particular, this meant selecting a technique that could visualize arbitrarily many sets using circles. There is only one circle-based Euler diagram drawing technique has this property: iCircles [37, 39]. Unlike venneuler, iCircles never omits regions that should have non-zero area. This is because the technique uses, where necessary, multiple circles to represent some sets; see Fig 2, where the set Travel is represented by two circles. Again, we manually placed numbers in the regions to indicate the cardinality of set intersections. See the middle diagrams in Figs 1 and 2 for examples.

When adding numerical annotations to the venneuler and iCircles diagrams, the numbers were placed approximately in the centre of the regions. Where regions were present that represented empty sets (and so should have zero area) no number was included, in part to avoid placing 0 in what could be very small regions and to reduce clutter. The labels indicating the sets represented by the curves were also manually positioned—for all three treatments—to ensure they were close to the associated curve and did not obstruct other parts of the diagram. When this was not possible, labels were placed further from their curve with a line used to indicate the association; see the middle diagram in Fig 1, where the rightmost Food label is linked by a line to its circle.

**Fig 4:**
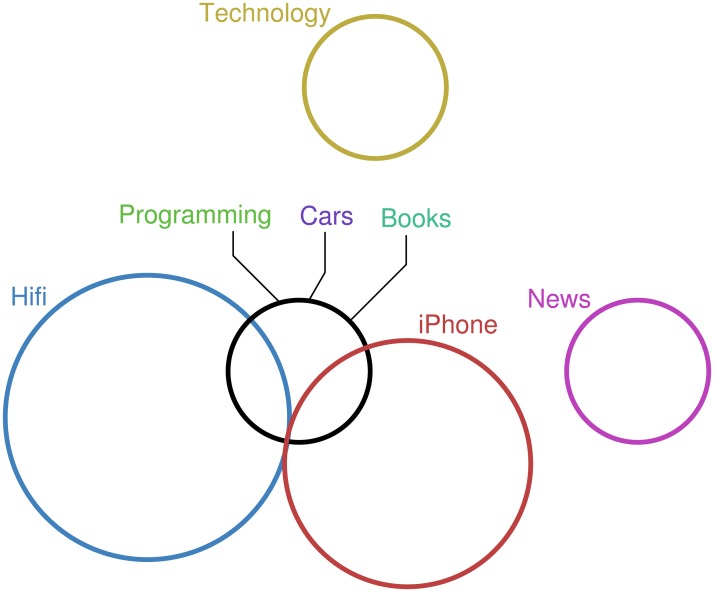
Concurrent curves are depicted in black.

By default, venneuler uses colour fill for its circles which is the same colour as the circle itself. In addition, iCircles uses a fixed colour palette for circle borders, with no interior fill. Since these techniques were devised, it has been found that using a unique colour for each set with no interior fill best supports task performance [4]. Following [4], we used colorbrewer [16] to select a set of perceptually distinguishable colours to assign to the circles and did not use an interior fill. An exception to this occurred when circles ran concurrently. In this case, black was assigned to the concurrent circles; see Fig 4. Each label was treated with the same colour as its associated circle. However, when the circle was black, the relevant colours were used for its labels.

When adding numerical annotations to linear diagrams, the numbers were placed at the top of the associated overlap. When there was insufficient space for the numerical annotation, which only occurred in the length-proportional case, the number was slightly offset and ‘connected’ using a line; see the bottom diagram in Fig 3. Unlike the Euler diagram cases, there were never overlaps present that represented empty sets. In particular, linear diagrams—length proportional or otherwise—always have the correct overlaps present (i.e. exactly one overlap for each non-empty set intersection and no extra overlaps). The colour palette used was the same as for Euler diagrams.

### Tasks and training

It was important to get users to perform tasks that focused on set cardinalities. As proportions alone cannot convey exact cardinality information, unlike numerical annotations, the tasks required the relative sizes of set cardinalities to be compared. The identified tasks fell into two categories, which we call ‘set cardinalities’ (S) and ‘intersection cardinalities’ (I), each with two subcategories:

S>: identify the sets whose cardinality is *more than* that of some specified set.
S<: identify the sets whose cardinality is *less than* that of some specified set.
I>: identify the set intersections whose cardinality is *more than* that of some specified set intersection.
I<: identify the set intersections whose cardinality is *less than* that of some specified set intersection.

Each task was presented with a set of five options, from which the participants had to select the right answers; there was always at least one right answer. To make the tasks accessible, the questions were presented in the context of information about the interests of people, using the data sets described in the section entitled *Data for Visualization*; the task type assigned to each data set is shown in the bottom rows of Tables 1 and 2.

We give example S> and I< tasks, the others are similar. Appealing to Fig 2, participants were asked to “Tick the checkboxes where there the total number of people interested in that topic is **more than** than the total number of people interested in **Relaxation**”, with options: (1) Android, (2) Bands, (3) College, (4) Travel, (5) None of the above. The correct answer is to select all four sets: Android, Bands, College and Travel. The top diagram, which uses proportions, visually indicates this by the size of the Relaxation circle being smaller than the other four circles. From the middle diagram, which uses numbers, it can be seen that two people are interested in Relaxation but 16 people are interested in Android. The numbers inside each remaining circle sum to more than two and, so, have more people interested in them than Relaxation. The bottom diagram shows that more people are interested in Relaxation using both area proportions and numbers.

For Fig 3, participants were asked to “Tick the checkboxes where **fewer people** have exactly that combination of interests than **all of Games, Internet and Technology only**”, with the following options: (1) Games only, (2) Games, Music and Technology only, (3) Bands only, (4) Hifi only, (5) None of the above. In each of the three linear diagrams, the second overlap corresponds to “all of Games, Internet and Technology only” since it includes three line segments, one for each of the named sets. The right-most overlap corresponds to Games only (this overlap includes just a line segment for Games and nothing else). In the top diagram, the Games only overlap is longer than that for Games, Internet and Technology only, so more people are interested in Games only, not fewer. The middle diagram shows that three people are interested in Games, Internet and Technology only whereas eight people are interested in Games only. The bottom diagram uses both length proportions and numbers to show fewer people are interested in Games only. Similarly, the other three relevant overlaps indicate more people, not fewer, are interested in the respective sets. Thus, the correct answer is ‘None of the above’.

**Fig 5:**
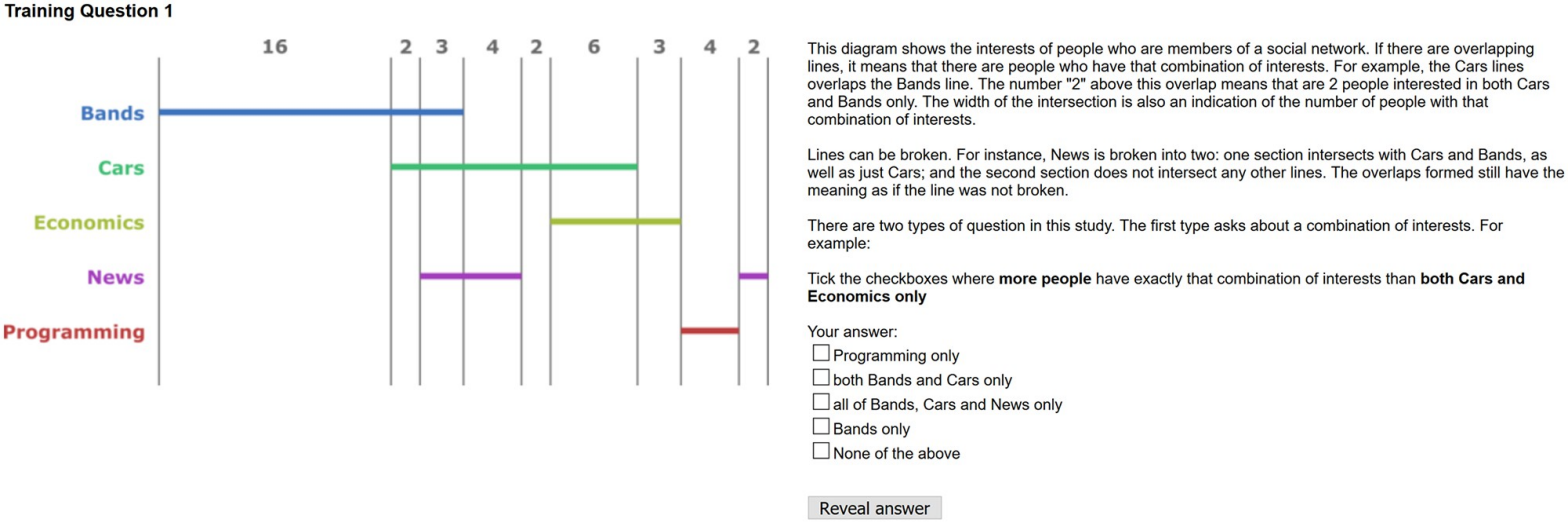
The first training page.

**Fig 6:**
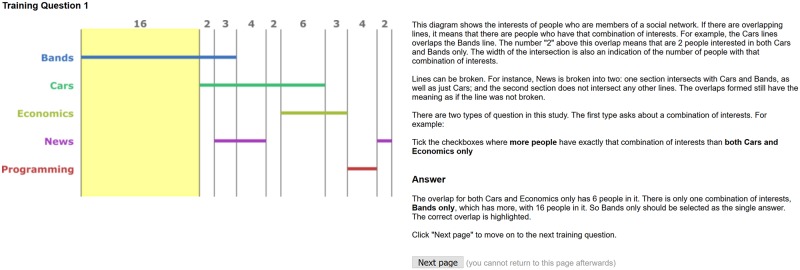
The first explanation page.

Participants were given training in how to read the diagrams and how to answer each of the four questions. An example of the first training question for a participant exposed to linear diagrams with proportions and numbers can be seen in Fig 5. The explanation page, revealed after the participant has submitted their answer is shown in Fig 6. Further details on the training material can be found in the submitted supplementary material and on the web site: www.eulerdiagrams.com/cardinality/.

### Data collection method

Amazon Mechanical Turk was used to crowdsource participants from the general population [7, 18, 30]. In MTurk, the tasks are called HITS—Human Intelligence Tasks—which are completed by anonymous participants. They are paid if they *successfully* complete the HIT. Crowdsourcing is a robust and upcoming method used by the research community for studies of the kind reported on in this paper [18, 30]. The tasks were implemented by adapting the template provided by [26]. In both the training phase and the main study phase, each question was displayed on a separate page of the HIT. Participants could not return to pages and subsequent pages were not revealed until the previous answer was submitted. Unlike the training questions, which were presented in the same order for all participants, in the main study the questions were randomly ordered. Participants were instructed to “maintain concentration on the HIT and answer questions without delay, unless a question explicitly allows you to take a break, in which case you can have a rest before continuing”.

It is recognised that some MTurk participants do not give questions their full attention, or have difficulties with the language used, and this is hard to control [7]. We call such participants *inattentive*.

**Fig 7:**
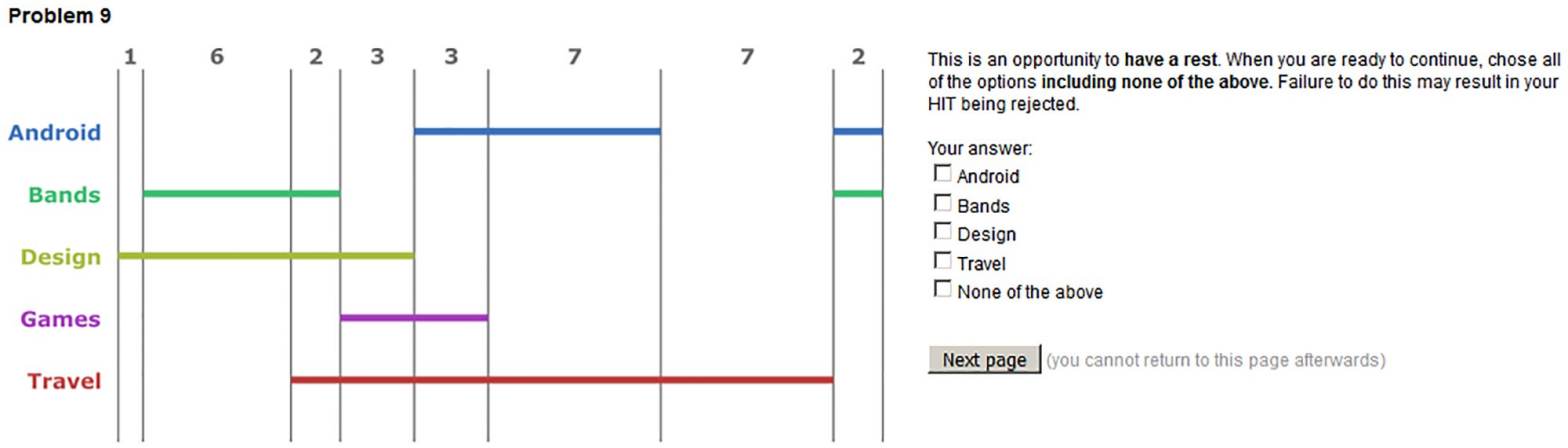
A question designed to identify inattentive participants.

To reduce the impact of language issues, a system qualification was used, allowing only participation from people based in the USA with a HIT approval rate of 95%. Another technique to identify inattentive participants is to include questions that require careful reading yet are trivial to answer [28]. In our study, we included two such questions. They asked participants to select all five options, including the none of the above checkbox, as their answer and were told if they failed to do so that they may not be paid for the HIT. An example of such a question is given in Fig 7. Participants were classified as inattentive if failed to click all checkboxes on either of the two inattentive participant-identifying questions. Attentive participants were paid $3.70 for taking part.

All data obtained from inattentive participants was removed before analysis and these two questions always appeared on the 14^th^ and 28^th^ pages of the HIT. In total, the HIT had 40 pages: a consent page, four training pages, 32 main study questions, two inattentive questions, and a final page that collected demographic information.

### Statistical methods

One technique is judged to be more effective than another if users can perform tasks significantly more accurately with it or, if no significant accuracy difference exists, significantly faster. Whilst we view accuracy as the most important indicator of performance differences, all analysis that was performed is reported in the paper. Throughout, results are declared significant if *p* < 0.05.

To analyse the data, we avoided using popular statistical tests for group comparisons, such as ANOVA and Kruskal-Wallis. For example, the accuracy and time data provided by each individual are expected to be correlated, and thus violating the independence assumption of the ANOVA and Kruskal-Wallis tests. Instead, we employ the generalized estimating equations models [22, 40] to analyse the accuracy and time data sets because they can account for the presence of unknown correlation between individual-specific responses.

Here we present in detail the statistical models used for the first two studies, each of which included three treatment groups. The models used for the last study were simpler, as there were only two treatments.

For each of the three empirical studies, we employed two local odds ratios generalized estimating equations models [40] to analyse the accuracy data. The first model, for the first two studies, was employed to answer RQ1, RQ2 and RQ3, compared the visualization types overall irrespective of task category:

$$\begin{array}{ccc} \log[\frac{\mathrm{Pr}(Y_{ik}\leq j)}{1-\mathrm{Pr}(Y_{ik}\leq j)}] & \;=\; & \beta_{0j}+\beta_{1}x_{ik1}+\beta_{2}x_{ik2} \end{array}$$

where

- Pr(*Y*_*ik*_ ≤ *j*) is the probability for subject *i* to provide at most *j* − 1 correct answers to question *k* and where *j* = 1, …, 5. *j* takes values from 1 to 5 since, for each question, there are 5 checkboxes which need to be correctly checked or not.
- *x*_*ik*1_ is the indicator that the diagram given to subject *i* for answering question *k* was *proportional*, and
- *x*_*ik*2_ is the indicator that the diagram given to subject *i* for answering question *k* was *both proportional and numerical*

for *i* = 1, …, *n*, given *n* participants, and *k* = 1, …, 32. With this model, we could determine whether the odds of providing *j* or fewer correct answers for one of the visualization types (proportional, numerical, or both) was significantly different from others while taking into account the expected correlation among the responses provided by each individual participant. For example, we can compare the odds of the proportional and of the numerical visualization type by calculating exp(*β*_1_). This quantity is equal to the ratio of the odds of providing *j* or fewer correct answers for the proportional visualization type to the odds of providing *j* or fewer correct answers for the numerical visualization type. If exp(*β*_1_) = 1 than the proportional and the numerical visualization types are equally accurate. If exp(*β*_1_) is less (greater) than one, then this implies that the proportional visualization method is more (less) accurate than the numerical method. The unknown parameters *beta*’s are estimated by the data. Once this is achieved, we can declare statistical significance based on *p*-values of the test that the odds ratio under study is equal to one or not. To quantify the variability of the data, we also provide a confidence interval of the odds ratio under study.

The second model, for the first two studies, was employed to see whether the answers to RQ1, RQ2 and RQ3 still held when we take into account task category (set or intersection):

$$\begin{array}{ccc} \log[\frac{\mathrm{Pr}(Y_{ik}\leq j)}{1-\mathrm{Pr}(Y_{ik}\leq j)}] & \;=\; & \beta_{0j}+\beta_{1}x_{ik1}+\beta_{2}x_{ik2}+\beta_{3}x_{ik3}+\\ & & \beta_{4}\left(x_{ik1}\times x_{it3}\right)+\beta_{5}\left(x_{ik2}\times x_{ik3}\right) \end{array}$$

where the variables are as above and, in addition, *x*_*ik*3_ is the indicator that the diagram given to subject *i* for answering question *k* was of type *I*. This model allowed us to estimate the odds of providing *j* or fewer correct answers with one combination of visualization type and task category compared to other combinations and determine whether significant differences existed.

For the time data, we used two generalized estimation models [22] that allowed us to estimate whether the time taken to provide answers was significantly different. Again we present details of the two models used for the first two studies. Following a similar approach for the accuracy data, the first of the two models directly addressed RQ1, RQ2 and RQ3 and the more complex model delved deeper into the data to see whether task category was important. The more complex model is given here, which accounts for the different combinations of visualization type (proportional, numerical, or both) and task category (set or intersection):

$$\begin{array}{ccc} \log(t_{ik}) & = & \delta_{0}+\delta_{1}x_{ik1}+\delta_{2}x_{ik2}+\delta_{3}x_{ik3}+\\ & & \delta_{4}\left(x_{ik1}\times x_{ik3}\right)+\delta_{5}\left(x_{ik2}\times x_{ik3}\right) \end{array}$$

where

- *t*_*ik*_ is the time needed for subject *i* to answer question *k*, and
- *x*_*ik*1_, *x*_*ik*2_ and *x*_*it*3_ are defined in the previous model

for *i* = 1, …, *n*, given *n* participants, and *k* = 1, …, 32. This model allowed us to estimate the ratio of times of answering a question, given a pair of combinations of visualization type and task category; the simpler model excluded all terms involving *x*_*ik*3_. As with the accuracy data, we provide *p*-values to declare statistical significance and confidence intervals to measure the variability of these ratios. Note that SD errors of timings are not appropriate for measuring variability since the timings for each individual are not independent.

Statistical output is included in the submitted supplementary material. We report on the main findings in the following sections.

## Euler diagrams: Results

Initially a pilot study collected data from 90 people, 30 per group. This identified a missing number from one of the numerical-group diagrams and another question had an erroneous answer. Five other questions were associated with minor bugs in the data collection software. All problems were rectified. For the main study, 300 people were recruited, 100 for each group; allocation to groups was random in this and both subsequent studies. Of these 300 participants, 23 were identified as inattentive and, thus, had their data removed before analysis; these inattentive participants were distributed across the groups as follows: 7 in the Euler Diagrams—Proportional (ED-P) group, 6 in the Euler Diagrams—Numerical (ED-N) group, and 10 in the uler Diagrams—Proportional & Numerical (ED-P&N) group. This left data from 277 participants (142 M, 134 F, 1 undisclosed; ages 20–74, mean 36.5), with 93 participants (0 colourblind, 1 did not supply colourblind information) in the ED-P group, 94 participants (1 colourblind) in the ED-N group, and 90 participants (1 colourblind, 2 did not supply colourblind information) in the ED-P&N group.

### ED: Overall comparison

The *accuracy* rate was 82.1% (ED-P: 80.9%, ED-N: 83.9%, ED-P&N: 84.1%). Using the local odds ratio GEE-based method for the accuracy data we estimated a 95% confidence interval (CI) for the odds of providing *j* or fewer correct answers for one treatment than another, as well as *p*-values that allowed us to determine whether the treatments gave rise to significantly different accuracy performance. The estimated odds of having *j* or fewer correct answers with ED-P was 1.3158 times higher than that of ED-N with a 95% CI of (1.0582, 1.6360) and *p*-value of 0.0135 (given to 4d.p.). Therefore, ED-N supported significantly more accurate task performance overall than ED-P. This result and the remaining two comparisons are summarised in Table 3. Overall, we see that ED-N and ED-P&N both supported significantly better accuracy performance than ED-P. However ED-N and ED-P&N were not significantly different overall.

**Table 3:** **ED**: Overall accuracy analysis.

Treatments	Odds	CI	*p*-value	Sig.	Most Accurate
ED-P versus ED-N	1.3158	(1.0582, 1.6360)	0.0135	✓	ED-N
ED-P&N versus ED-N	0.8867	(0.6888, 1.1414)	0.3506		N/A
ED-P versus ED-P&N	1.4839	(1.1834, 1.8606)	0.0006	✓	ED-P&N

The mean *time* to answer a question was 35.5 seconds (ED-P: 31.0 seconds, ED-N: 39.7 seconds, ED-P&N: 35.8 seconds). Using the GEE-based method for the time data we estimated a 95% CI for the ratio of times of answering a question for pairs of treatments, again with a *p*-value to determine whether significantly different time performance exists. The estimated ratio of times of answering a question with ED-P compared to ED-N was 0.7809 with a 95% CI of (0.7082, 0.8612) and *p*-value of <0.0001 (to 4d.p.). Therefore, ED-P supported significantly faster task performance overall than ED-N. This result and the remaining two comparisons are summarised in Table 4. Overall, we found that ED-P supported significantly faster time performance than the other two treatments. However ED-N and ED-P&N were not significantly different overall in terms of time.

**Table 4:** **ED**: Overall time analysis.

Treatments	Ratio	CI	*p*-value	Sig.	Fastest
ED-P versus ED-N	0.7809	(0.7082, 0.8612)	0.0000	✓	ED-P
ED-P&N versus ED-N	0.9126	(0.8189, 1.0170)	0.0980		N/A
ED-P versus ED-P&N	0.8557	(0.7742, 0.9459)	0.0023	✓	ED-P

In *summary*, ED-N and ED-P&N supported the most accurate task performance overall yet were both slower than ED-P. Given accuracy is taken to be more important than time, Euler diagrams with numerical annotations and area-proportional Euler diagrams with numerical annotations are deemed the most effective visualizations overall.

### ED: Set cardinalities

The *accuracy* rate was 90.5% (ED-P: 90.0%, ED-N: 89.1%, ED-P&N: 92.5%). The mean *time* to answer a question was 30.6 seconds (ED-P: 24.4 seconds, ED-N: 36.4 seconds,ED-P&N: 31.1 seconds). The statistical results are in Tables 5 and 6. In *summary*, viewing accuracy as the most important performance indicator, area-proportional Euler diagrams with numerical annotations best support set cardinality tasks, followed by area-proportional Euler diagrams. Euler diagrams with numerical annotations were least effective for set cardinality tasks.

**Table 5:** **ED**: Set cardinality accuracy analysis.

Treatments	Odds	CI	*p*-value	Sig.	Most Accurate
ED-P versus ED-N	0.8070	(0.6083, 1.0704)	0.1367		N/A
ED-P&N versus ED-N	0.5059	(0.3544, 0.7221)	0.0002	✓	ED-P&N
ED-P versus ED-P&N	1.5950	(1.0942, 2.3251)	0.0152	✓	ED-P&N

**Table 6:** **ED**: Set cardinality time analysis.

Treatments	Ratio	CI	*p*-value	Sig.	Fastest
ED-P versus ED-N	0.6839	(0.6224, 0.7516)	<0.0001	✓	ED-P
ED-P&N versus ED-N	0.8751	(0.7879, 0.9719)	0.0127	✓	ED-P&N
ED-P versus ED-P&N	0.7816	(0.7073, 0.8636)	<0.0001	✓	ED-P

### ED: Intersection cardinalities

The *accuracy* rate was 75.3% (ED-P: 71.8%, ED-N: 78.5%, ED-P&N: 75.6%). The mean *time* to answer a question was 40.4 seconds (ED-P: 37.6 seconds, ED-N: 43.0 seconds, ED-P&N: 40.5 seconds). The statistical results are in Tables 7 and 8. In *summary*, for intersection cardinality tasks, either area-proportional Euler diagrams with numerical annotations or Euler diagrams with numerical annotations can be used to support the most effective task performance: they both lead to significantly more accurate task performance than area-proportional Euler diagrams, yet are indistinguishable from each other taking into account both accuracy and time.

**Table 7:** **ED**: Intersection cardinality accuracy analysis.

Treatments	Odds	CI	*p*-value	Sig.	Most Accurate
ED-P versus ED-N	1.8624	(1.4451, 2.4002)	<0.0001	✓	ED-N
ED-P&N versus ED-N	1.2664	(0.9449, 1.6972)	0.1139		N/A
ED-P versus ED-P&N	1.4706	(1.1563, 1.8704)	0.00170	✓	ED-P&N

**Table 8:** **ED**: Intersection cardinality time analysis.

Treatments	Ratio	CI	*p*-value	Sig.	Fastest
ED-P versus ED-N	0.8917	(0.7972, 0.9973)	0.0448	✓	ED-P
ED-P&N versus ED-N	0.9517	(0.8450, 1.0719)	0.4146		N/A
ED-P versus ED-P&N	0.9369	(0.8377, 1.0479)	0.2542		N/A

### ED: Summary of results

The analysis revealed that, whilst ED-P&N and ED-N were indistinguishable overall and for intersection cardinality tasks, ED-P&N better supported set-cardinality tasks, giving this treatment the edge. The ED-P group performed particularly poorly: it was significantly less accurate than both ED-N and ED-P&N overall and for intersection cardinality tasks, and significantly less accurate than ED-P&N for set cardinality tasks. However, ED-P supported significantly better time performance overall and in all but one other case (for intersection cardinality tasks, compared to ED-P&N). Fast performance alongside a higher error rate, though, is not desirable. These results lead us to suggest that, out of these three treatments and for the tasks considered in our study, area-proportional Euler diagrams with numerical annotations should be used to visualize information about sets and their cardinalities. Thus for RQ1 we answer yes—numerical annotations enhance the use of area-proportional Euler diagrams—and for RQ2 we answer no—the use of numerical annotations alone is not more effective.

In terms of effect sizes, the odds of providing *j* or fewer correct answers overall with ED-P was 1.4839 times that of ED-P&N, which increased to 1.5950 for set cardinality tasks and reduced to 1.4706 for intersection cardinality tasks; in all cases, there is a clear accuracy improvement with ED-P&N. This is at the expense of time, where we estimated that ED-P tasks were, overall, completed in 85.57% of the time of ED-P&N tasks; this decreased to 78.16% of the time for set cardinality tasks and increased to 93.69% of the time for intersection cardinality tasks. The odds of providing *j* or fewer correct answers was 0.5059 using ED-N compared to ED-P&N for set cardinality tasks which is substantial in practical terms. ED-N also brought a time penalty for these tasks: ED-P&N participants were estimated to complete tasks in 87.51% of the time compared to using ED-N.

### ED: Interpretation of results

Firstly, we consider set cardinality tasks. For these tasks, participants exposed to ED-P need to compare circle areas. A possible advantage of ED-P&N is that the participants may choose either to compare circle areas or exploit the numerical annotations. The theory of graphical perception suggests it is difficult to accurately judge the relative values of two or more areas if the circles are not in close proximity to each other or the values encoded are similar [10, 14]. Making quantitatively accurate judgements when comparing different circle areas is also cognitively difficult [24]. These perceptual insights support our finding that ED-P performed significantly worse than ED-P&N for set cardinality tasks: we found that the numerical annotations in combination with circle area overcame the limitations inherent in representing set cardinality using area alone. This helps to explain why, for set cardinality tasks using Euler diagrams, RQ1 is answered affirmatively: numerical annotations enhance the use of area proportions.

When examining the improved performance in set cardinality tasks for ED-P&N over ED-N we hypothesise that the proportional areas of ED-P&N supported the arithmetical calculations needed to answer the questions. In some cases, the ED-N areas were contradictory to the required answer, so potentially causing confusion or inaccuracy, even though the ED-N group’s training did not reference areas.

The question then arises as to why, for set cardinality tasks, ED-N did not outperform ED-P and, moreover, ED-P was *faster* than ED-N. First, we suggest that the ED-N group has an increased cognitive load when mentally calculating the cardinality of sets relative to the load associated with comparing circle areas using ED-P. This is likely to explain the speed benefit of ED-P. Confusing situations may also arise when mentally calculating the cardinality of two or more sets: the relative areas of the circles may mismatch the relative sums resulting from the calculations. This could cause doubt over the correctness of the mental arithmetic again causing a time penalty. These insights help to explain why, for set cardinality tasks using Euler diagrams, RQ2 is answered negatively: numerical annotation alone is not more effective.

Focusing on intersection cardinality tasks, ED-P performed particularly poorly: it was significantly less accurate than both other treatments. In general, when visualizing quantitative values, area cannot be accurately perceived [23]. This suggests that region area is likely to be a poor graphical property to manipulate when visualizing set-intersection cardinalities. This explains why ED-P is less accurate as it relies on area comparisons: the difficultly of perceiving differences in areas is particularly challenging for this task since the regions are non-uniform shapes. Moreover, for ED-N and ED-P&N, no mental arithmetic is required for this task: the numerical annotations present in the diagrams can be directly compared. EP-N and ED-P&N were not significantly different, suggesting that numerical annotations are the most important indicator of set-intersection cardinality, further reinforcing our positive answer to RQ1 and negative answer to RQ2 when using Euler diagrams.

Drawing on all the discussion, this study supports the principle that area is a poor graphical property to manipulate when visualizing quantitative data. However, we found that the limitations of area are not only overcome by the inclusion of numerical annotations but the combination of them is superior to annotation alone.

## Linear diagrams: Results

A pilot study collected data from 90 people, 30 per group. No problems were identified so we proceeded with the main study. We recruited 300 people, 100 for each group. Of these, 28 were identified as inattentive and, thus, had their data removed before analysis; these inattentive participants were distributed across the groups as follows: 12 in the Linear Diagrams—Proportional (LD-P) group, 7 in the Linear Diagrams—Numerical (LD-N) group, and 9 in the Linear Diagrams—Proportional & Numerical (LD-P&N) group. This left data from 272 participants (131 M, 141 F; ages 18–71, mean 36.1), with 88 participants (0 colourblind, 2 did not supply colourblind information) in the LD-P group, 93 participants (1 colourblind) in the LD-N group, and 91 participants (3 colourblind) in the LD-P&N group.

### LD: Overall comparison

The *accuracy* rate was 82.1% (LD-P and LD-N: both 81.7%, LD-P&N: 82.8%). The mean *time* to answer a question was 36.4 seconds (LD-P: 32.3 seconds, LD-N: 37.6 seconds, LD-P&N: 39.2 seconds). The statistical results are in Tables 9 and 10. In *summary*, whilst no significant differences existed in accuracy performance, the LD-P group performed significantly faster overall. Therefore, length-proportional linear diagrams are taken to be the most effective overall visualization method, out of the three linear diagram variants.

**Table 9:** **LD**: Overall accuracy analysis.

Treatments	Odds	CI	*p*-value	Sig.	Most Accurate
LD-P versus ED-N	1.1348	(0.9400, 1.3702)	0.1882		N/A
LD-P&N versus LD-N	1.1018	(0.8897, 1.3644)	0.3741		N/A
LD-P versus LD-P&N	1.0300	(0.8457, 1.2570)	0.7690		N/A

**Table 10:** **LD**: Overall time analysis.

Treatments	Ratio	CI	*p*-value	Sig.	Fastest
LD-P versus LD-N	0.8134	(0.7436, 0.8898)	<0.0001	✓	LD-P
LD-P&N versus LD-N	0.9798	(0.8980, 1.0689)	0.6457		N/A
LD-P versus LD-P&N	0.8302	(0.7636, 0.9026)	<0.0001	✓	LD-P

### LD: Set cardinalities

The *accuracy* rate was 91.7% (LD-P: 92.3%, LD-N: 90.5%, LD-P&N: 92.4%). The mean *time* to answer questions was 29.5 seconds (LD-P: 24.5 seconds, LD-N: 31.1 seconds, LD-P&N:32.6 seconds). The statistical results are in Tables 11 and 12. In *summary*, for set cardinality tasks, our results suggest that length-proportional linear diagrams are the most effective visualization method out of the three compared linear diagram variants.

**Table 11:** **LD**: Set cardinality accuracy analysis.

Treatments	Odds	CI	*p*-value	Sig.	Most Accurate
LD-P versus LD-N	1.0264	(0.7906, 1.3332)	0.8454		N/A
LD-P&N versus LD-N	1.2253	(0.9403, 1.5967)	0.1324		N/A
LD-P versus LD-P&N	0.8367	(0.6366, 1.1021)	0.2057		N/A

**Table 12:** **LD**: Set cardinality time analysis.

Treatments	Ratio	CI	*p*-value	Sig.	Fastest
LD-P versus LD-N	0.7846	(0.7208, 0.8541)	<0.0001	✓	LD-P
LD-P&N versus LD-N	0.9971	(0.9151, 1.0864)	0.9462		N/A
LD-P versus LD-P&N	0.7870	(0.7258, 0.8533)	<0.0001	✓	LD-P

### LD: Intersection cardinalities

The *accuracy* rate was 72.5% (LD-P: 71.1%, LD-N: 72.9%, LD-P&N: 73.3%). The mean *time* to answer questions was 43.4 seconds (LD-P: 40.0 seconds, LD-N: 44.1 seconds, LD-P&N: 45.8 seconds). The statistical results are in Tables 13 and 14. In *summary*, our study suggests that length-proportional linear diagrams support the most effective task performance, when considering intersection cardinality tasks. The addition of numerical annotations hindered task performance and numerical annotations alone were not as effective for intersection cardinality tasks.

**Table 13:** **LD**: Intersection cardinality accuracy analysis.

Treatments	Odds	CI	*p*-value	Sig.	Most Accurate
LD-P versus LD-N	1.2039	(0.9450, 1.5339)	0.1332		N/A
LD-P&N versus LD-N	1.0342	(0.7835, 1.3650)	0.8125		N/A
LD-P versus LD-P&N	1.1641	(0.9025, 1.5015)	0.2419		N/A

**Table 14:** **LD**: Intersection cardinality time analysis.

Treatments	Ratio	CI	*p*-value	Sig.	Fastest
LD-P versus LD-N	0.8433	(0.7601, 0.9355)	0.0013	✓	LD-P
LD-P&N versus LD-N	0.9628	(0.8731, 1.0618)	0.4477		N/A
LD-P versus LD-P&N	0.8758	(0.7959, 0.9638)	0.0067	✓	LD-P

### LD: Summary of results

The empirical study revealed that, in terms of accuracy, all three treatments are equally as effective: no significant performance differences were revealed. However, in all cases, length-proportional linear diagrams supported significantly faster task performance than both length-proportional linear diagrams with numerical annotations and linear diagrams with numerical annotations. In conclusion, out of these three treatments and for the tasks considered in our study, we suggest that length-proportional linear diagrams should be used to visualize information about sets and their cardinalities. Thus for RQ1 we answer no—numerical annotations do not enhance the use of length-proportional linear diagrams—and for RQ2 we also answer no—the use of numerical annotations alone is not more effective.

As no significant accuracy performances existed, we focus our attention on time when considering effect sizes. We estimated that LD-P tasks were, overall, completed in 81.34% of the time of LD-N tasks; this decreased to 78.46% of the time for set cardinality tasks and increased to 84.33% of the time for intersection cardinality tasks. In comparison to LD-P&N, we estimated that LD-P tasks were, overall, completed in 83.02% of the time of LD-P&N tasks; this decreased to 78.70% of the time for set cardinality tasks and increased to 87.58% of the time for intersection cardinality tasks.

### LD: Interpretation of results

When using LD-P, both the set-cardinality and intersection-cardinality tasks required participants to use estimation to compare the length of line segments (i.e. the total lengths of the lines for sets or the lengths of overlaps for intersections). The same strategy could be used by the LD-P&N group whereas the LD-N group had to rely on mental arithmetic for the set cardinality tasks or simply comparing numerical annotations for the intersection cardinality tasks.

Our interpretation of the results begins by focusing on intersection-cardinality tasks. Considering LD-P and LD-P&N, we know that length is considered an effective graphical element to manipulate when visualizing quantitative values [23], making the annotations redundant. This explains why there was no accuracy difference in this case. We posit that the numerical annotations were either still used by participants in the LD-P&N group to reinforce their responses or acted as a distracter [13], evidenced by the slower response time. Regarding LD-P and LD-N, the lengths are just as accurately compared as the numerical annotations, but our results imply the cognitive process of identifying and comparing numbers is slower than just comparing lengths.

For set-cardinality tasks, the reasons for LD-P’s superior performance are more extreme: for LD-P&N and LD-N, our data suggest that for both these groups, participants exploited mental arithmetic when performing the tasks. This is because there were no significant time differences between these two groups but they were both significantly slower than LD-P. It is clear that the act of addition resulted in a slower performance than the act of comparing the total length of several line segments by estimation as is required in the case of LD-P, but with no benefit in accuracy.

LD-P proved to be the most effective visualization technique, supporting the negative answers to both RQ1 and RQ2: numerical annotations do not enhance linear diagrams, with or without proportions, irrespective of the task. Drawing on all the discussion, this study supports the principle that length is an effective graphical property to manipulate when visualizing quantitative data. Interestingly, we find that the inclusion of numerical annotations impedes task performance in terms of speed without ever impacting upon accuracy.

## Comparing Euler and linear diagrams

The first two studies revealed two visualization methods as the most effective: area-proportional Euler diagram with numerical annotations (ED-P&N) and length-proportional linear diagrams without numerical annotations (LD-P). The purpose of this section is to suggest which of these two competing choices most effectively supports performance when focusing on cardinality-oriented tasks.

The pilot collected data from 60 people, 30 per group; no problems were identified. For the main study, we recruited 200 people, 100 for each group. Of these, 15 were identified as inattentive and, thus, had their data removed before analysis; these inattentive participants were distributed across the groups as follows: 5 in the ED-P&N group and 10 in the LD-P group. Therefore, the analysis is based on data from 185 participants (85 M, 100 F; ages 19–67, mean 36.7), with 95 participants (3 colourblind, 1 did not supply colourblind information) in the ED-P&N group and 90 participants (4 colourblind) in the LD-P group.

### ED-P&N vs LD-P: Overall comparison

The *accuracy* rate was 82.6% (ED-P&N: 83.7%, LD-P: 81.3%). The local odds ratio GEE-based model for the accuracy data estimated a 95% CI for the odds of providing *j* or fewer correct answers for ED-P&N versus LD-P. The odds were 0.7815 with a CI of (0.6250, 0.9772) and a *p*-value of 0.0306. Overall, ED-P&N supported significantly more accurate task performance than LD-N. The mean *time* to answer questions was 33.8 seconds (ED-P&N: 36.2 seconds, LD-P: 31.2 seconds). The 95% CI for the odds of providing answers in less time for ED-P&N than with LD-P was computed using the GEE-based method. The ratio was 1.1596 with a CI of (1.0373, 1.2963) and a *p*-value of 0.0092. Overall, the LD-P group performed significantly faster than the ED-P&N group. In *summary*, taking accuracy as the primary performance indicator, our study suggests that, overall, area-proportional Euler diagrams should be used to visualize sets and their cardinalities to perform these task types.

### ED-P&N vs LD-P: Set cardinalities

The *accuracy* rate was 91.1% (ED-P&N: 90.8%, LD-P: 91.3%). Using the local odds ratio GEE-based method for the accuracy data we again estimated 95% CI for set-cardinality tasks only, and the associated *p*-value: the computed odds were 0.9628 with a CI of (0.6884, 1.3466) and a *p*-value of 0.8248. Therefore, there was no significant accuracy performance difference between ED-P&N and LD-P for set-cardinality tasks. The mean *time* to answer questions was 27.6 seconds (ED-P&N: 31.0 seconds, LD-P: 24.1 seconds). Using the GEE-based method for the time data we estimated the ratio of time taken to provide an answer using ED-P&N compared to LD-P to be 1.2556. The associated 95% CI was (1.1310, 1.3910) with a *p*-value of <0.0001. Here, we see that LD-P supported significantly faster time performance than ED-P&N. In *summary*, our study suggests that LD-N supports significantly better performance than ED-P&N for set-cardinality tasks.

### ED-P&N vs LD-P: Intersection cardinalities

The *accuracy* rate was 74.1% (ED-P&N: 76.6%, LD-P: 71.3%). Using the local odds ratio GEE-based method for the accuracy data we estimated that the odds of providing *j* or fewer correct answers using ED-P&N compared to LD-P to be 0.6572 with a 95% CI of (0.5113, 0.8446) and a *p*-value of 0.0010. Hence, ED-P&N supported significantly more accurate performance than LD-P for intersection-cardinality tasks. The mean *time* to answer an intersection-cardinality question was 39.9 seconds (ED-P&N: 41.5 seconds, LD-P: 38.3 seconds). The GEE-based method for the time data estimated the time ratio using ED-P&N compared to LD-P was 1.0710 with a 95% CI of (0.9448, 1.2139) and a *p*-value of 0.2836. Thus, for intersection-cardinality tasks, no significant time performance exists between ED-P&N and LD-P. In *summary*, based on the accuracy results, ED-P&N best supported task performance for intersection-cardinality tasks.

### ED-P&N vs LD-P: Summary of results

The final analyses, which compared the most effective Euler and linear variants, have revealed that different treatments bring different benefits. Overall and for intersection-cardinality tasks, we found that ED-P&N supports the most effective task performance, when viewing accuracy as the most important indicator. In terms of effect sizes, the odds of providing *j* or fewer correct answers overall with LD-P&N was 0.7815 times that of ED-P&N, which reduced to 0.6572 for intersection-cardinality tasks; in these two cases, there is a clear accuracy improvement with ED-P&N. This is at the expense of time, where we estimated that ED-P&N tasks were, overall, completed in 115.96% of the time of LD-P tasks (recall, no significant time difference existed for intersection-cardinality tasks).

For set-cardinality tasks, we found that LD-P, whilst not significantly more accurate, was significantly faster than ED-P&N. In particular, we estimated that ED-P&N tasks took 125.56% of the time for set-cardinality tasks as compared to LD-P.

There is a mixed conclusion from this study for RQ3. If one wishes to perform both set-cardinality and intersection-cardinality tasks, or just the latter, area-proportional Euler diagrams with numerical annotations are the most effective of the two visualization techniques. However, if one only wishes to perform set-cardinality tasks, no significant accuracy differences were revealed but length-proportional linear diagrams will support faster task performance. Given that we view accuracy as more important than time, we tend to suggest using area-proportional Euler diagrams with numerical annotations for performing tasks involving the cardinalities of sets and their intersections.

### ED-P&N vs LD-P: Interpretation of results

It is known that using length to convey quantitative values is more effective than areas [10, 23]. Ignoring the impact of numerical annotations, this would suggest that LD-P would outperform ED-P&N, regardless of task type. However, this is not universally supported by our results since, with respect to accuracy, ED-P&N was superior overall and for intersection-cardinality tasks. This suggests that numerical annotations not only overcome the limitations of using area alone (RQ1 for the ED case) but enhances the use of area so much that it outperforms using length alone, with the exception of set-cardinality tasks.

Considering intersection tasks specifically, the LD-P group needed to compare the lengths of overlaps, each of which is a single line segment or part thereof. For ED-P&N, participants could either compare areas, which we know is cognitively inferior to comparing line lengths, or compare the numerical annotations (without any mental arithmetic required for this task type). Taken with our results, this leads us to posit that participants using ED-P&N relied on comparing the annotations rather than the *irregular* areas of the regions to yield the significantly better accuracy performance. Interestingly, this did not come at any time penalty compared to LD-P, presumably because no mental arithmetic was required.

The set-cardinality tasks paint a different picture: LD-P was significantly faster than ED-P&N whereas there were no significant accuracy differences. Regarding accuracy, our results suggest that, given the difficulty in comparing circle areas [24], the summation of the numerical annotations—where relied on by participants—can be performed just as accurately as comparing line lengths. Unsurprisingly, the reduced cognitive effort of *comparing line lengths* as opposed to *comparing circle areas supported by the summation of numerical annotations*, led to faster performance.

Tying these insights together, taken with the mean accuracy rates for the tasks, the fact that ED-P&N was more accurate overall appears to follow from the fact that it was significantly more accurate for intersection-cardinality tasks. By contrast, LD-P was significantly faster overall, which appears to follows from the fact that it was faster for the set-cardinality tasks, potentially reinforced by participants’ performance on the intersection cardinality tasks (here, LD-P had a higher accuracy rate, but not significantly so). In conclusion, for RQ3, we found that ED-P&N was most effective overall, since we view accuracy as a more important indicator of performance than time.

## Threats to validity

Threats to validity are categorized as internal, construct and external [31]. With regard to internal validity, which examines whether confounding factors impact the results, a major consideration in the design of all three studies related to carry-over effect. This threat occurs when the measures arising from a treatment are affected by another treatment. To eliminate this, we used between group designs and participants could only take part once.

Construct validity focuses on whether the dependent variables (error rate, false negatives, and time) and independent variables (questions and treatments) are accurate measures to test the hypotheses. Participants could give incorrect responses if the diagrams were drawn in such a way that cognition was hindered (this could also increase time taken). However, one of our goals was to evaluate the output of implemented diagram generation software, so we had little control over certain layout variations, such as the centre points of the circles in the Euler diagram case. We manually positioned the labels and selected an effective colour palette to assign to the sets, thus overriding any software defaults (these graphical properties are readily altered, post-layout). Moreover, the manual choice and assignment of colours reduced variation between treatments. In addition, the set names were chosen so that no two sets in a single diagram started with the same letter, to reduce the potential for misreading. To ensure the rigour of time measurements, so far as is reasonably practicable in crowd-sourced studies, and to reduce the impact of fatigue, the two ‘inattentive’ questions encouraged participants to take a break before proceeding to the next question.

Lastly, we focus on external validity, examining the extent to which the results generalise. Firstly, only two types of task were considered: those which focused on sets and those which focused on exhaustive set intersections. Other tasks could have been considered, such as those which focus on non-exhaustive set intersections. For instance, referring to Fig 1, are more people interested in *Bands* than *iPhone and Programming*? The set bands contains 4+ 2 = 6 elements whereas there are 8+ 3 = 11 elements in iPhone and Programming, so the answer is no. It is important to gain further insight, so see whether performance differences exist for other task types.

There is also a limitation with venneuler that impacted on the study design: not all data sets can be appropriately visualized. Sometimes there are regions missing that should be present and other times there are regions present that should be missing. The former case is particularly problematic and arises due to the restriction on using circles; some of the SNAP data sets gave rise to such diagrams and were, thus, discounted from use in our studies—this limitation is of practical relevance. As the number of sets grows, it is more likely that non-empty set intersections are missing from the generated diagrams, causing erroneous deductions to be made. By contrast, this problem does not arise with linear diagrams: it is always possible to accurately visualize relative set cardinalities using length proportions.

## Conclusion

The extensive occurrence of set-based data, and the many attempts to automatically visualize it, are primary drivers for understanding the cognitive impact of different visualization methods on user performance. Mindful of the importance of understanding such choices in real-world settings—that is, by using real-world data and actual implemented visualization systems—this paper addressed three research questions:

RQ1: are area- and length-proportional diagrams enhanced by numerical annotations reflecting actual set cardinality? For area-proportional Euler diagrams, our results support the addition of such annotations: accuracy performance was improved, but at a time penalty. This was true for tasks that required set cardinalities to be compared and, without a time penalty, for tasks that required set intersection cardinalities to be compared. For linear diagrams, the results were different: numerical annotations brought no accuracy improvement and also yielded a time penalty, for both task types. We speculate that this difference is due to the cognitive difficulty of comparing areas contrasted with the relative ease of comparing line lengths. Thus a key take away message for designers of visual systems for displaying set cardinality is that the use of proportions to convey size needs to be carefully considered before being adopted. At least for Euler diagrams, proportions alone are not ideal. Numerical annotations appear beneficial when other means of communicating size are relatively ineffective but bring no significant benefit otherwise. The implication is that designers of graphical notations need to be conscious of the fact that adding annotations need not yield performance benefits but can sometimes do so.

RQ2: is numerical annotation alone, without proportions, more effective? For both Euler and linear diagrams our studies suggest that the answer is no: overall, numerical annotations alone did not lead to superior task performance. For set-cardinality tasks, numerical annotations alone required participants to perform mental arithmetic. This did not impact on accuracy but brought with it a time penalty in both cases. For intersection tasks, the results are not so clear cut: for Euler diagrams, numerical annotation alone brought improved accuracy. This could be due to the approximate nature of the region areas (they need not be accurate) which is overcome by annotations, or the cognitive difficulty associated with comparing the areas of irregular shapes. A key takeaway message here is that graphical properties whose size can be manipulated to convey cardinality information appear to be generally useful as compared to numerical annotations.

RQ3: which technique is most effective overall and, thus, should be used in practice? Our results suggest that Euler diagrams with numerical annotations were most effective overall and, specifically, for intersection cardinality tasks. For set-cardinality tasks, length proportional diagrams gave rise to faster, but not less accurate performance. As we take accuracy to be more important than time, our studies lead us to suggest that the venneuler method, with numerical annotations, subject to the use of an effective colour palette and careful label placement, should be used in practice for these types of task as long as the data can be represented relatively accurately.

Despite our recommendation to use venneuler in practice, users must be aware of the practical limitations of the this method: for data sets of increasing complexity, the method produces diagrams with a variety of inaccurate layout features: regions with inaccurate areas that could lead to erroneous deductions being made (mitigated somewhat by the numerical annotations), non-empty set intersections that are not represented by regions in the diagram, and unwanted regions in the diagram that represent empty set intersections. Linear diagrams do not suffer the same drawbacks as they always accurately visualize the data. Therefore, it is important that future work ascertains the extent to which venneuler is limited in the context of real-world data and whether linear diagrams are still inferior for more complex data sets, where these erroneous features are more likely to arise.

Our results for RQ3 also lead us to hypothesize that the venneuler method could be further enhanced by the inclusion of annotations for the set cardinalities alongside their associated label (e.g. if the set Bands has 6 elements, label the associated circle Band (6), or similar). This would reduce the reliance on mental arithmetic to perform set-cardinality tasks and could give further performance benefits over length-proportional linear diagrams.

## Supporting information

S1 Appendix **Supplementary material for**: The efficacy of Euler diagrams and linear diagrams for visualizing set cardinality using proportions and numbers.
(PDF)

S1 Dataset **Dataset for study one**: Euler diagrams.
(CSV)

S2 Dataset **Dataset for study two**: Linear diagrams.
(CSV)

S3 Dataset **Dataset for study three**: Euler-P&N versus LD-P.
(CSV)
