# Supplementary material for: The efficacy of Euler diagrams and linear diagrams for visualizing set cardinality using proportions and numbers
Source: PLoS One. 2019 Mar 28;14(3):e0211234. doi: 10.1371/journal.pone.0211234 (PMC6438608; doi:10.1371/journal.pone.0211234)
Supplement: S1 Appendix — Supplementary material for: The efficacy of Euler diagrams and linear diagrams for visualizing set cardinality using proportions and numbers. (PDF) [file pone.0211234.s001.pdf]

Supplementary Material for: The efficacy of Euler diagrams and linear diagrams for visualizing set cardinality using proportions and numbers

Gem Stapleton[1*], Peter Chapman[2], Peter Rodgers[3], Anestis Touloumis[1], Andrew Blake[1], Aidan Delaney[4],

1 Centre for Secure, Intelligent and Usablity Systems,
School of Computing, Engineering and Mathematics, University of Brighton, Brighton, UK
2 School of Computing, Edinburgh Napier University, Edinbugh, UK
3 School of Computing, University of Kent, Canterbury, UK
4 Bloomberg, London, UK

* g.e.stapleton@brighton.ac.uk

This document is supplementary material for the associated paper appearing in PLOSOne. In contains the following information:

- Appendix A contains information on how the questions were generated for the tasks in the study.
- Appendix B presents the examples used for training participants.
- Appendix C presents the questions and associated diagrams used in the studies.
- Appendix D provides information on the statistical methods used to analyse the data.
- Appendices E-G present all of the statistical output generated from the methods employed for the three studies.

All of the diagrams included in this document are scaled to 50% of the actual size used in the study in order to fit within the page width.

A - Question Generation

For the S-type questions, a random permutation of the list of labels was generated using the website `www.random.org`. The first label in this permuted list then became the label which would appear in the question, and the next four labels in the permuted list were the labels which would appear as check-boxes, alongside the "None of the above" option. (Where there were only five sets in the diagram, the second step was redundant.)

For the I-type questions, a random permutation of the list containing those intersections containing at most three labels, was generated using the website `www.random.org`. The first intersection in this permuted list became the intersection whihc would appear in the question, and the next four intersetions in the permuted list were the intersections which would appear as check-boxes, alongside the "None of the above" option.

A check was performed on the number of check-boxes that appeared in the correct answer, which ranged from 0 (representing "None of the above") to 4. These numbers were:

- 0/"None of the above": 7 questions
- 1 check-box to be checked: 6 questions
- 2 check-boxes to be checked: 7 questions
- 3 check-boxes to be checked: 7 questions
- 4 check-boxes to be checked: 5 questions

Thus, there was no systematic bias towards any number of check-boxes appearing in the correct answer.

B - Training for Studies A, B & C

This section contains the data presented to participants for the purposes of training. After attempting each question, participants were given the correct answer, an explanation of the correct answer (with reference to the diagram) and a modified diagram with the regions of interest highlighted. An exception was for training question 4, as the correct answer was "None of the above", and thus no regions were highlighted. These modified diagrams for training questions 1 to 3 are presented alongside the respective question.

Question 1

- **Question:** Tick the check boxes where **more people** have exactly that combination of interests than **Cars and Economics only**.
- **Check boxes to be ticked:** Bands
- **Task Type:** Intersection comparison - More than.
- **SNAP Data Set Used:** 44130462
- ED-N

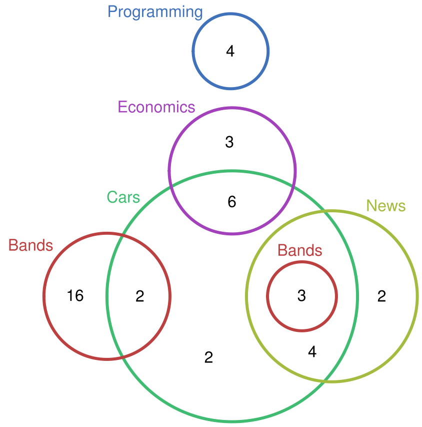

- ED-N:Explanation

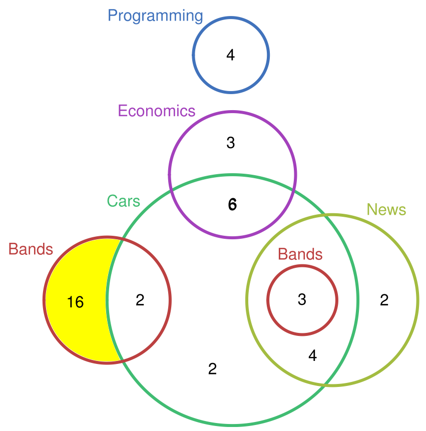

- ED-P

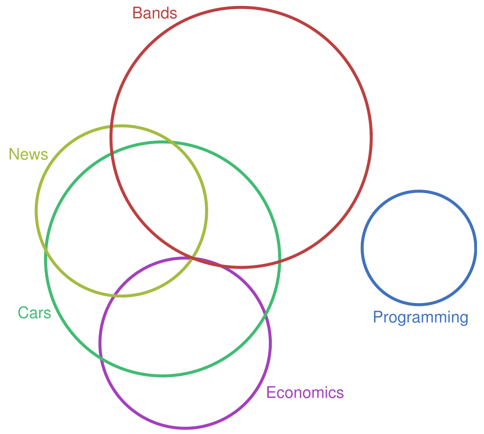

- ED-P:Explanation

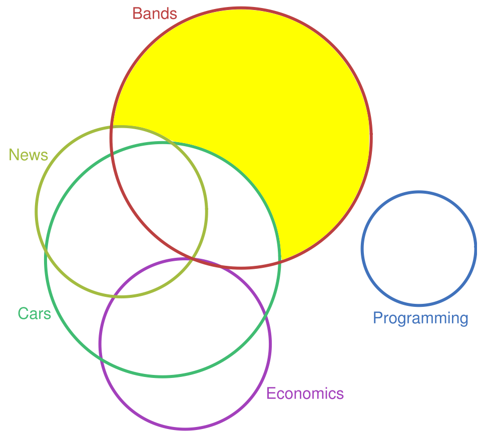

- ED-P&N

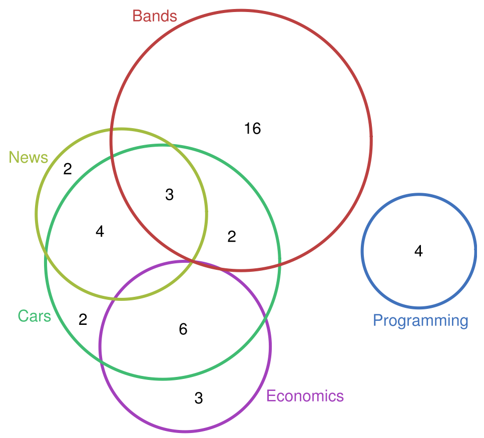

- ED-P&N:Explanation

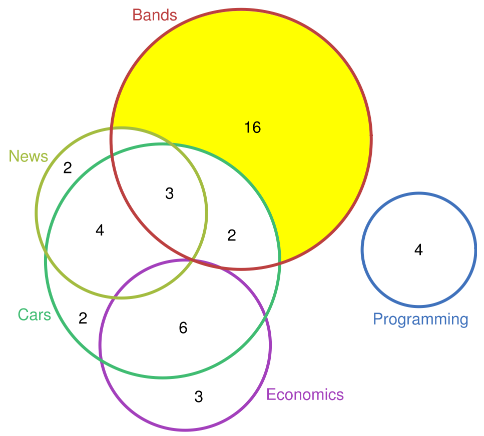

- LD-N

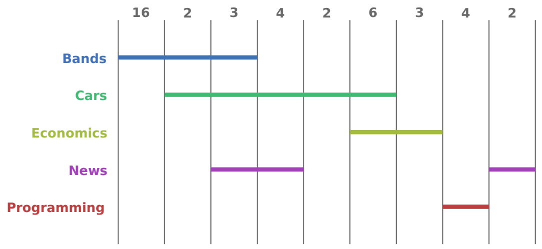

- LD-N:Explanation

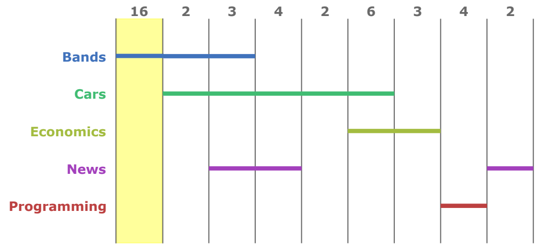

- LD-P

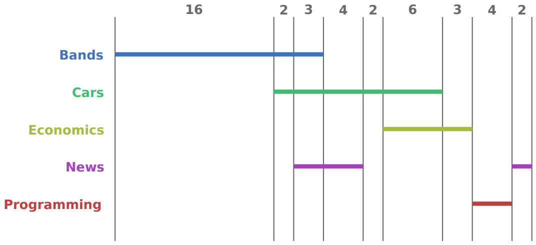

- LD-P:Explanation

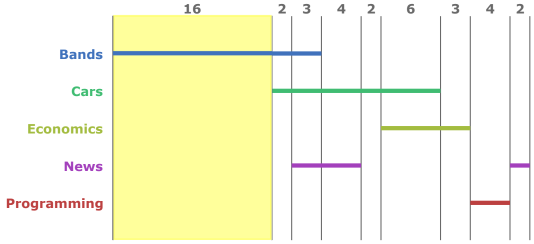

- LD-P&N

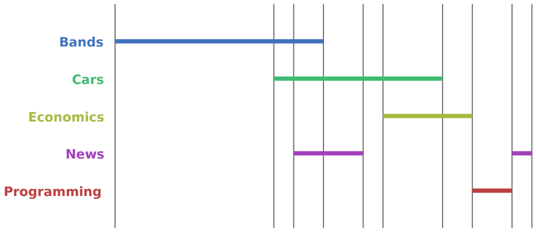

- LD-P&N:Explanation

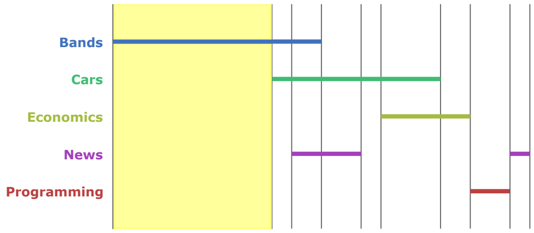

Question 2

- **Question:** Tick the check boxes where the total number of people interested in that topic is **less than** the total number of people interested in **Economics**.
- **Check boxes to be ticked:** Android, Design
- **Task Type:** Set comparison - Less than.
- **SNAP Data Set Used:** 16652550
- ED-N

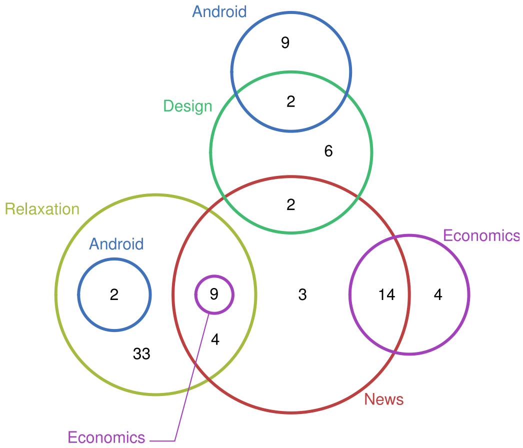

- ED-N:Explanation

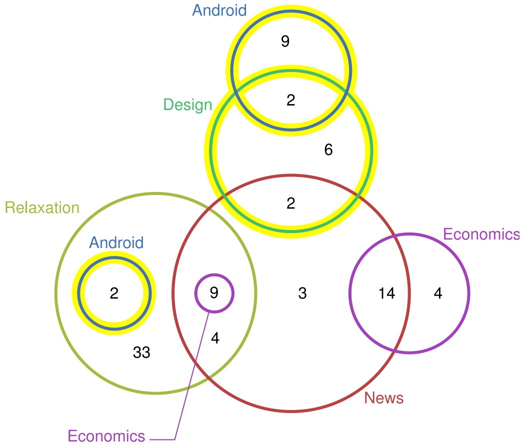

- ED-P

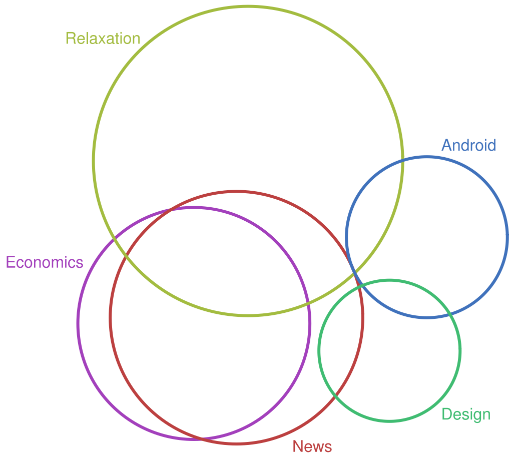

- ED-P:Explanation

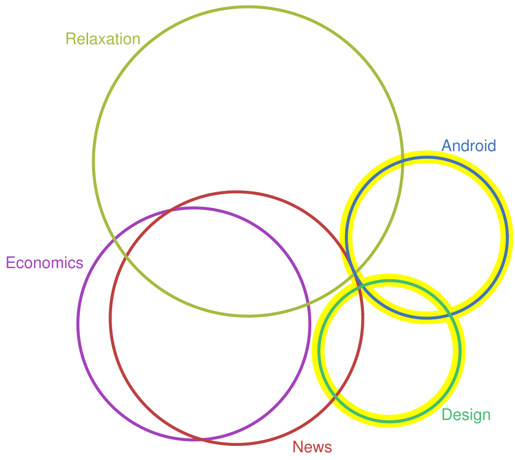

- ED-P&N

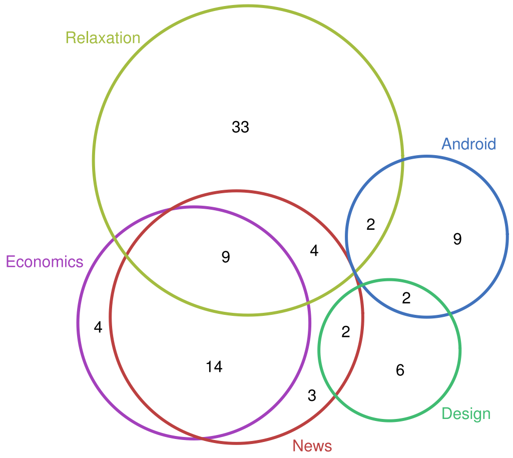

- ED-P&N:Explanation

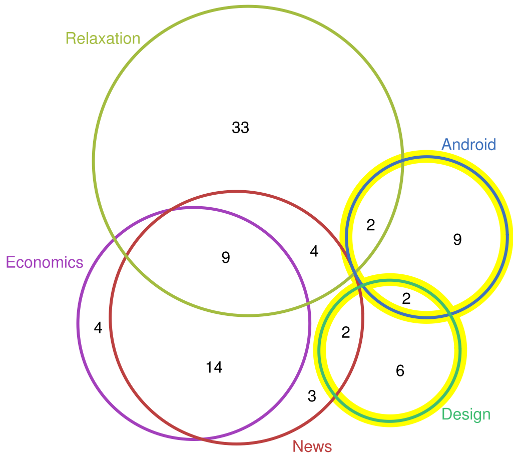

- LD-N

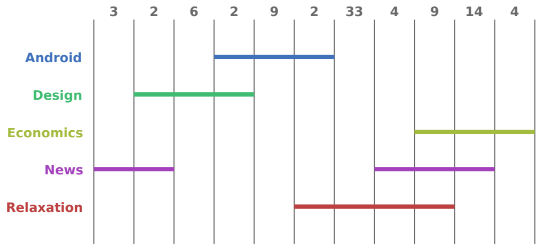

- LD-N:Explanation

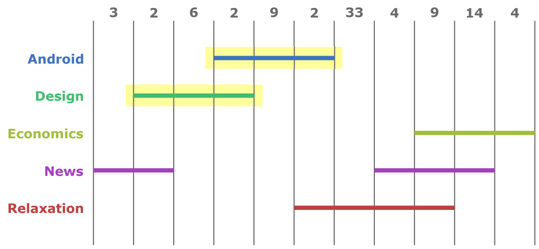

- LD-P

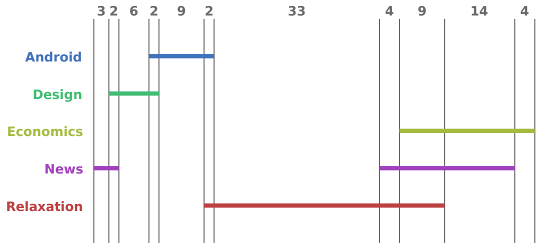

- LD-P:Explanation

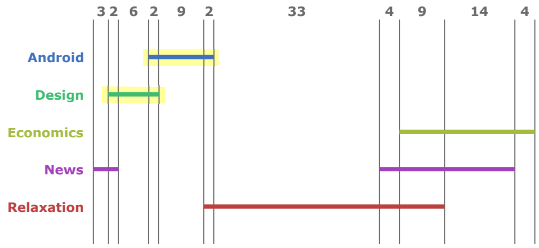

- LD-P&N

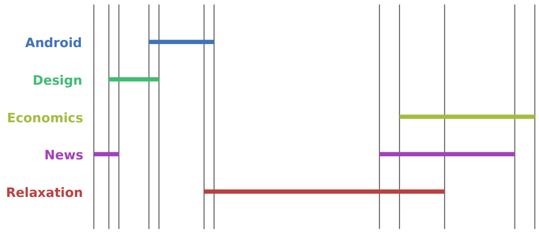

- LD-P&N:Explanation

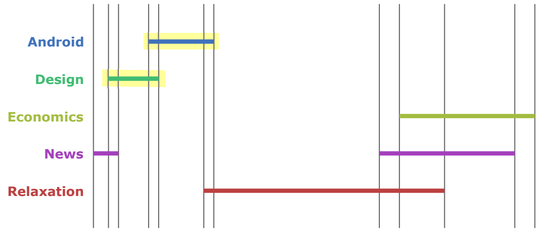

Question 3

- **Question:** Tick the check boxes where **more people** have exactly that combination of interests than **Games only**.
- **Check boxes to be ticked:** Books, Design and Health
- **Task Type:** Intersection comparison - More than.
- **SNAP Data Set Used:** 19492215
- ED-N

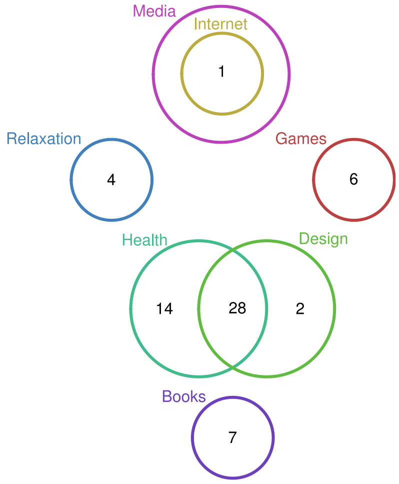

- ED-N:Explanation

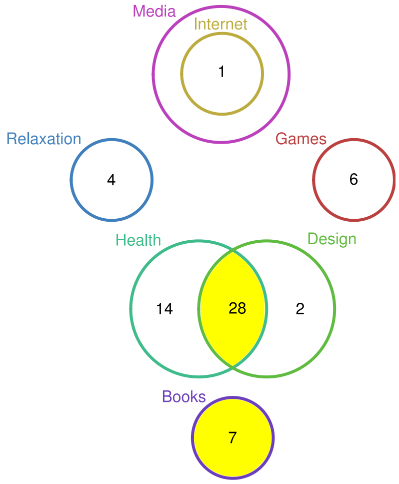

- ED-P

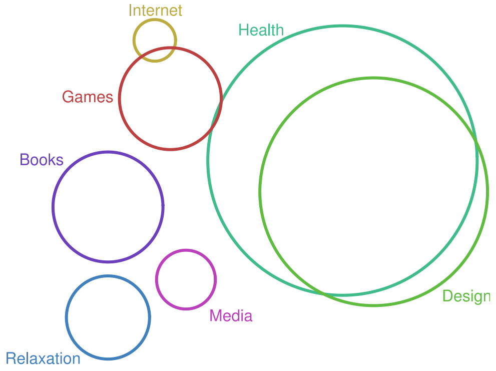

- ED-P:Explanation

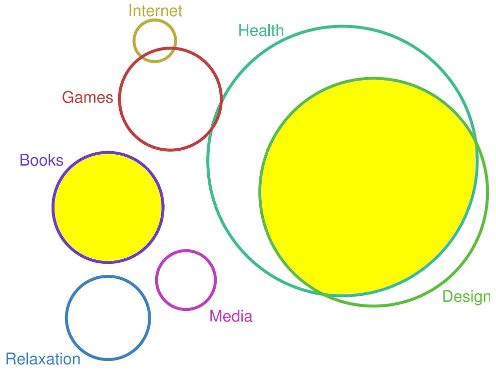

- ED-P&N

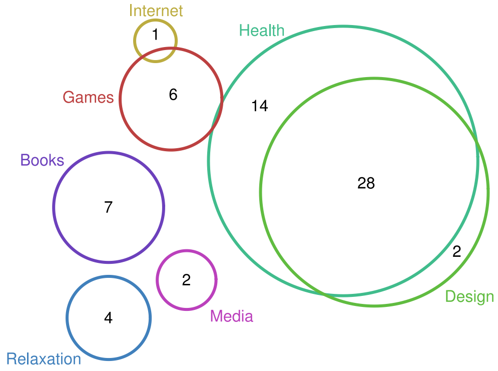

- ED-P&N:Explanation

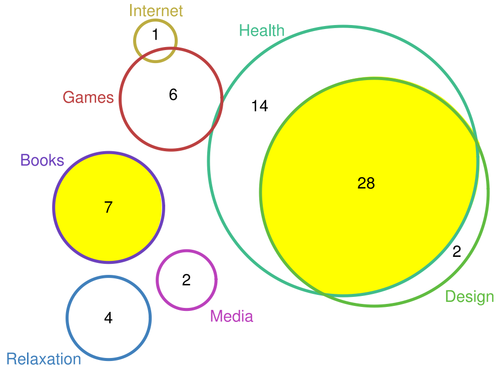

- LD-N

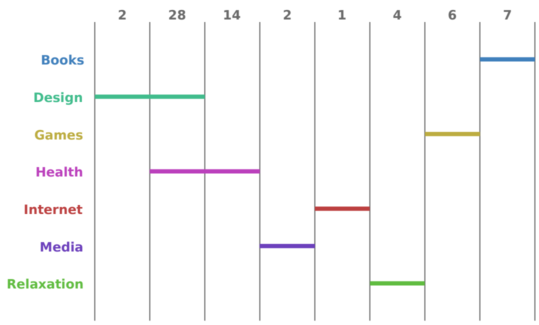

- LD-N:Explanation

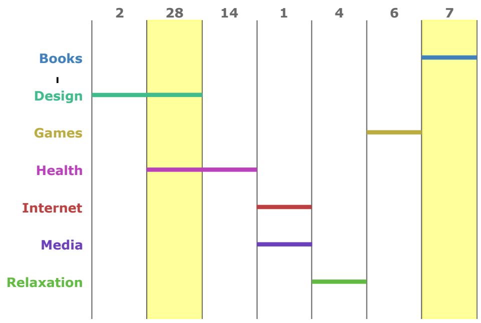

- LD-P

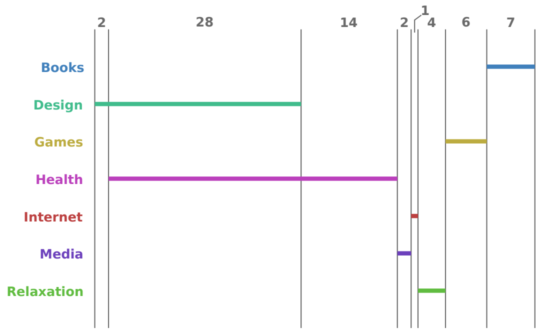

- LD-P:Explanation

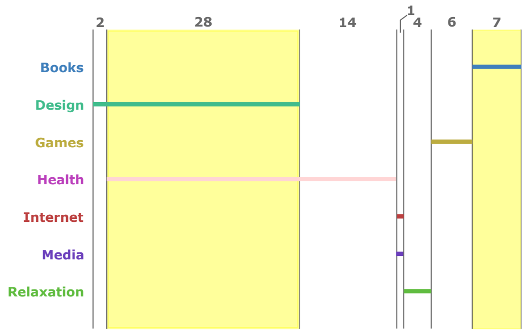

- LD-P&N

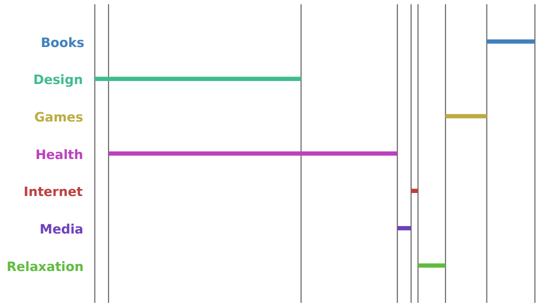

- LD-P&N:Explanation

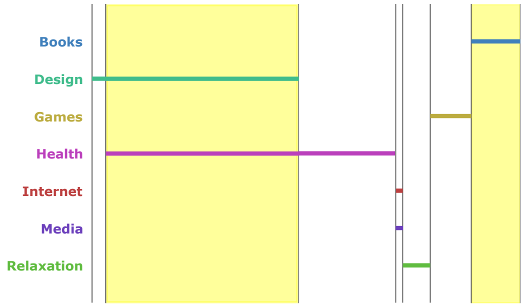

Question 4

- **Question:** Tick the check boxes where the total number of people interested in that topic is **greater than** the total number of people interested in **iPhone**.
- **Check boxes to be ticked:** None of the above
- **Task Type:** Set comparison - More than.
- **SNAP Data Set Used:** 105918870
- ED-N

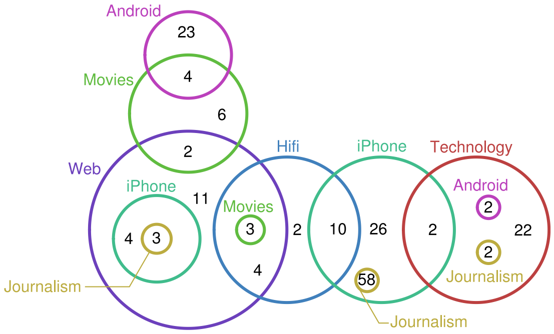

- ED-P

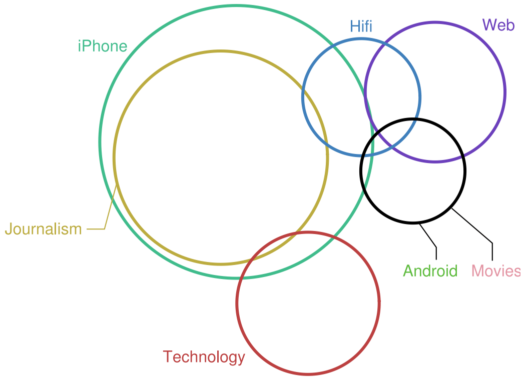

- ED-P&N

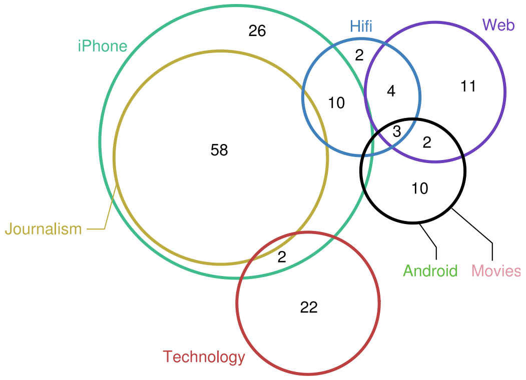

- LD-N

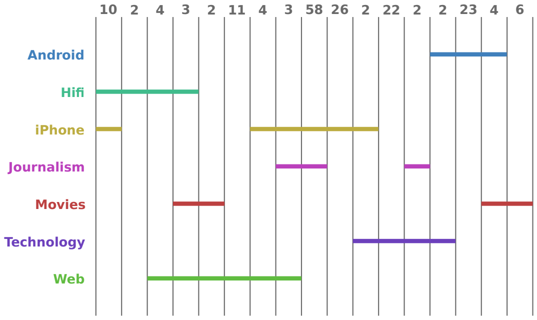
10 2 4 3 2 11 4 3 58 26 2 22 2 2 23 4 6
Android
Hifi
iPhone
Journalism
Movies
Technology
Web

- LD-P

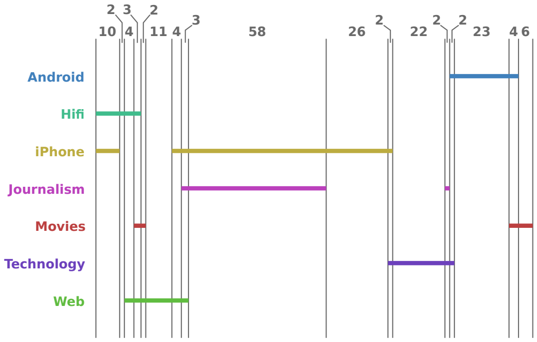
2 3 2 3 2 2 2
10 4 11 4 58 26 22 23 4 6
Android
Hifi
iPhone
Journalism
Movies
Technology
Web

- LD-P&N

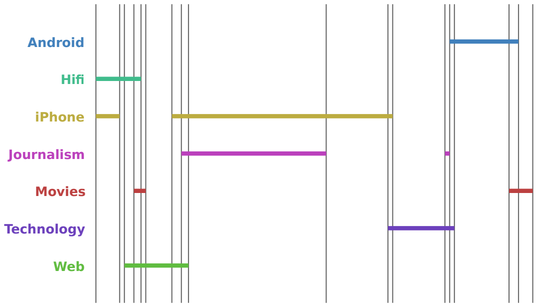
Android
Hifi
iPhone
Journalism
Movies
Technology
Web

C - Studies A, B & C

Question 1

- **Question:** Tick the check boxes where the total number of people interested in that topic is **greater than** the total number of people interested in **Relaxation**.
- **Check boxes to be ticked:** Android, Bands, College, Travel
- **Task Type:** Set comparison - More than.
- **SNAP Data Set Used:** 73024519
- ED-N

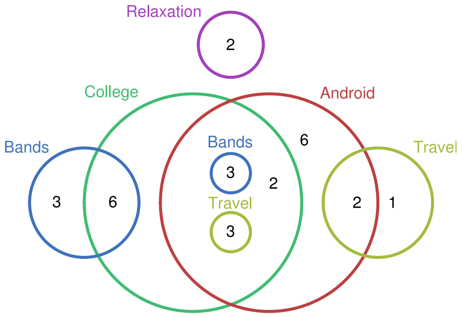

- ED-P

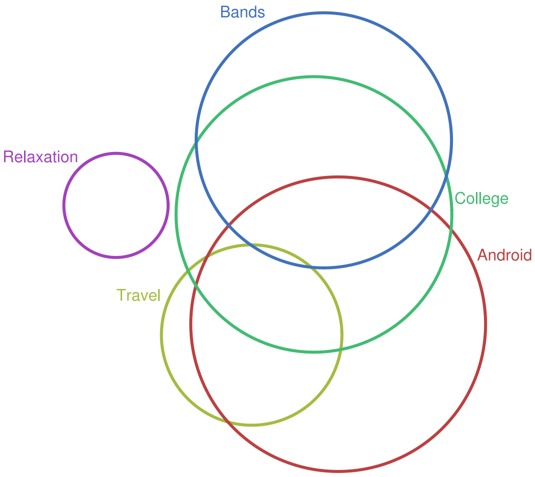

- ED-P&N

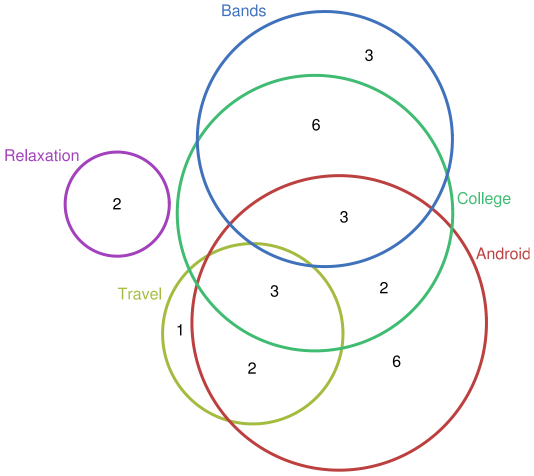
Bands
3
6
Relaxation
2
College
3
Android
Travel
3
2
1
2
6

- LD-N

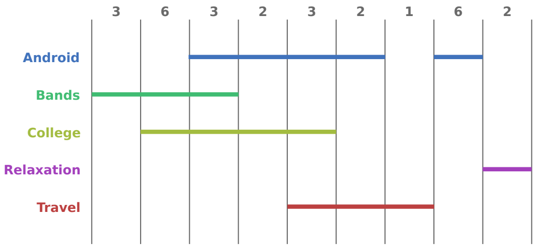
3 6 3 2 3 2 1 6 2
Android
Bands
College
Relaxation
Travel

- LD-P

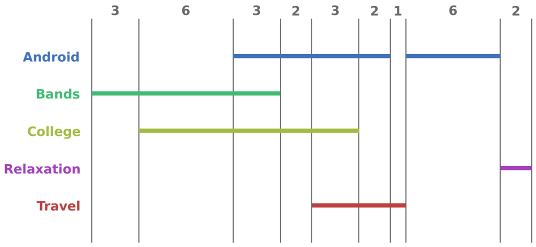
3 6 3 2 3 2 1 6 2
Android
Bands
College
Relaxation
Travel

- LD-P&N

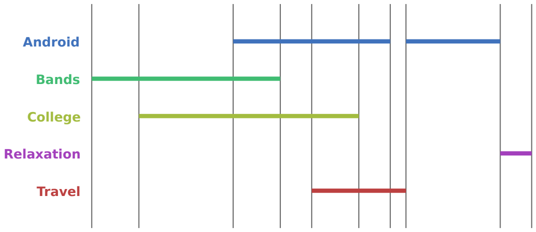
Android
Bands
College
Relaxation
Travel

Question 2

- **Question:** Tick the check boxes where the total number of people interested in that topic is **less than** the total number of people interested in **Programming**.
- **Check boxes to be ticked:** Bands, Games
- **Task Type:** Set comparison - Less than.
- **SNAP Data Set Used:** 73738774
- ED-N

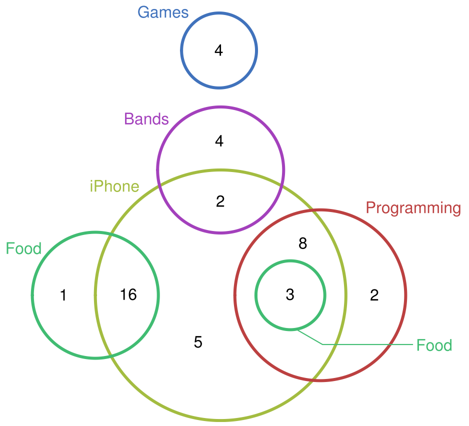

- ED-P

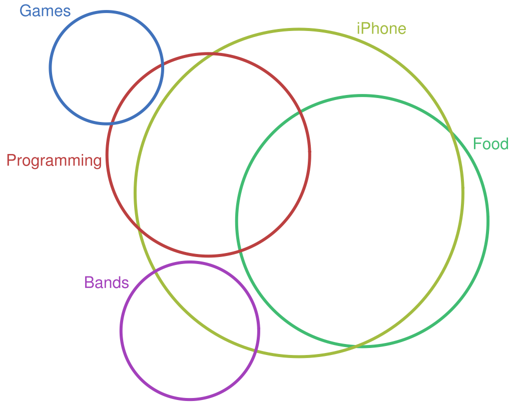

- ED-P&N

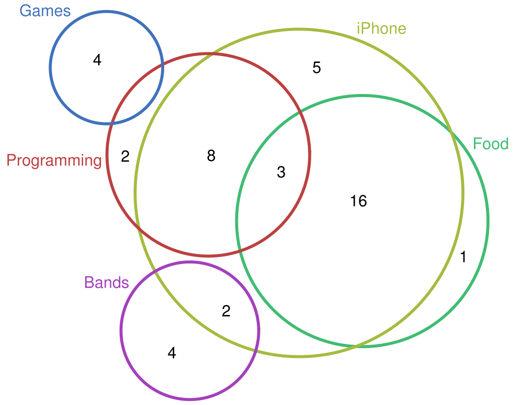
Games
4
iPhone
5
Programming
2
8
3
16
Food
1
Bands
2
4

- LD-N

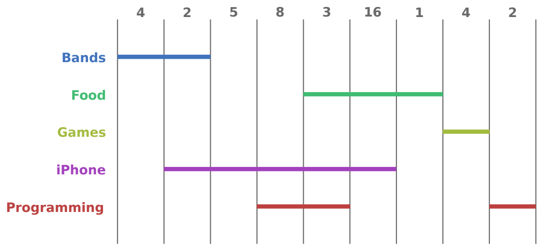
4 2 5 8 3 16 1 4 2
Bands
Food
Games
iPhone
Programming

- LD-P

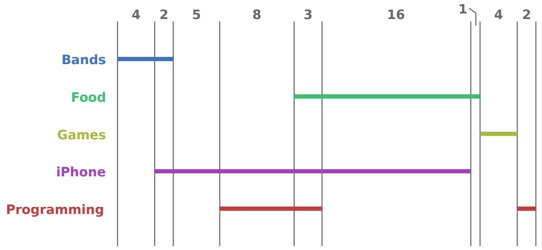
4 2 5 8 3 16 1 4 2
Bands
Food
Games
iPhone
Programming

- LD-P&N

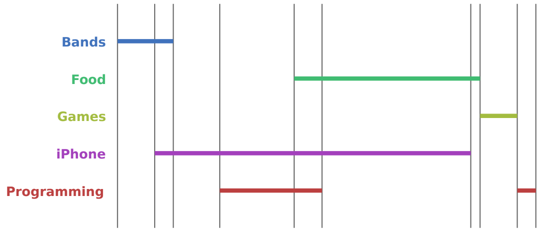
Bands
Food
Games
iPhone
Programming

Question 3

- **Question:** Tick the check boxes where **more people** have exactly that combination of interests than **News only**.
- **Check boxes to be ticked:** None of the above
- **Task Type:** Intersection comparison - More than.
- **SNAP Data Set Used:** 14957252
- ED-N

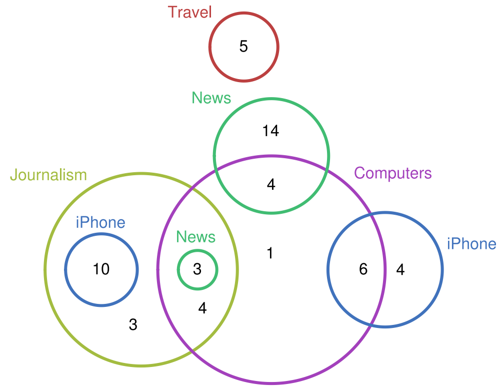

- ED-P

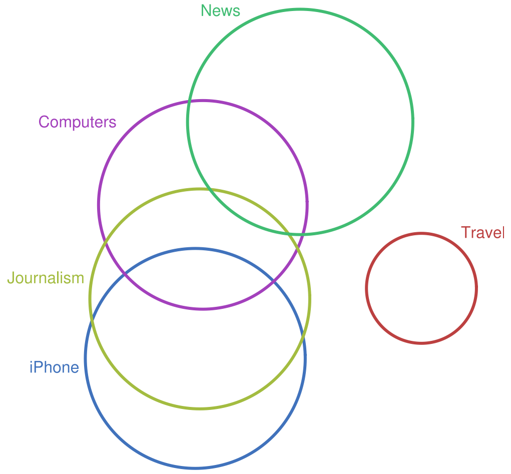

- ED-P&N

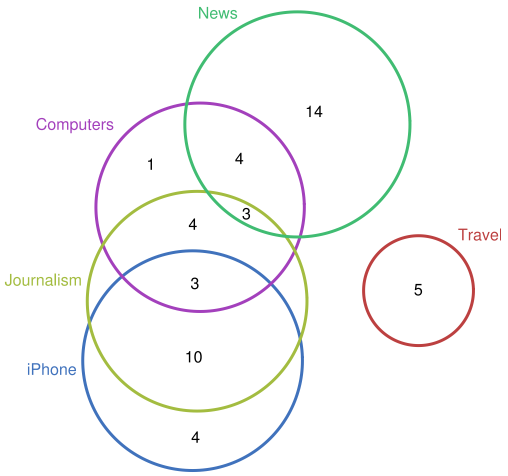
News
Computers
Travel
Journalism
iPhone
14
1
4
3
4
3
5
10
4

- LD-N

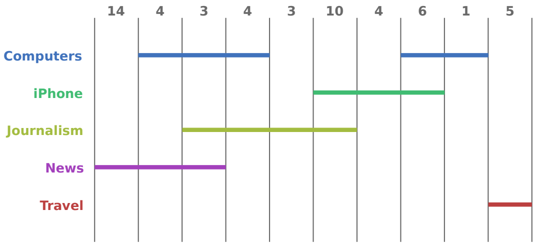
14 4 3 4 3 10 4 6 1 5
Computers
iPhone
Journalism
News
Travel

- LD-P

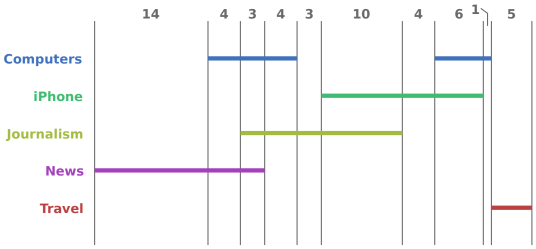
14 4 3 4 3 10 4 6 1 5
Computers
iPhone
Journalism
News
Travel

- LD-P&N

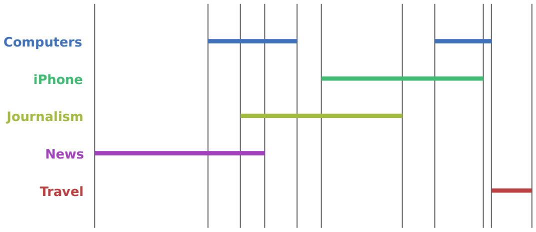
Computers
iPhone
Journalism
News
Travel

Question 4

- **Question:** Tick the check boxes where **more people** have exactly that combination of interests than **Design and Games only**.
- **Check boxes to be ticked:** None of the above
- **Task Type:** Intersection comparison - Less than.
- **SNAP Data Set Used:** 18687625
- ED-N

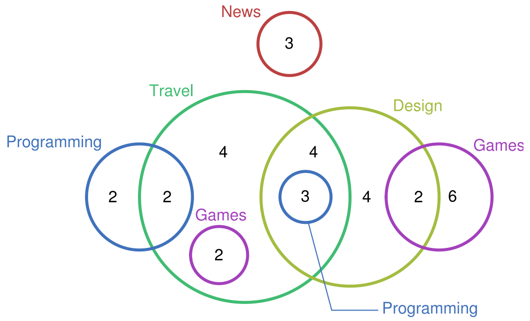

- ED-P

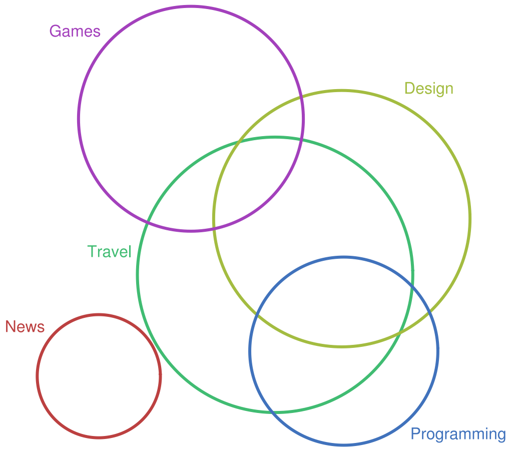

- ED-P&N

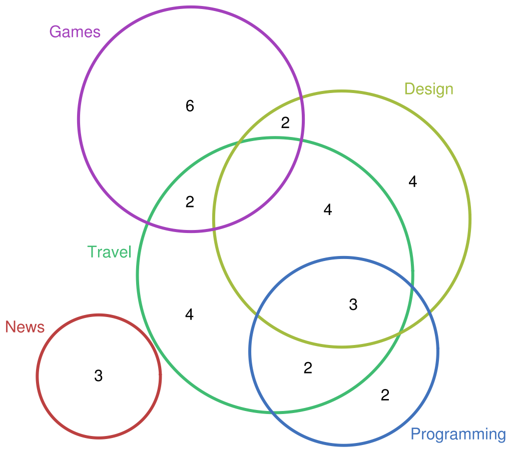

- LD-N

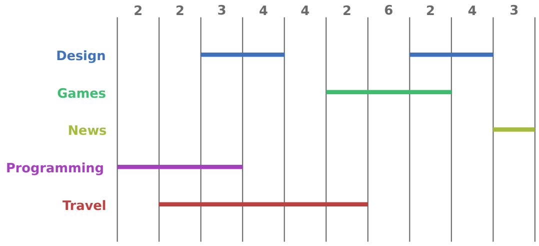
2 2 3 4 4 2 6 2 4 3
Design
Games
News
Programming
Travel

- LD-P

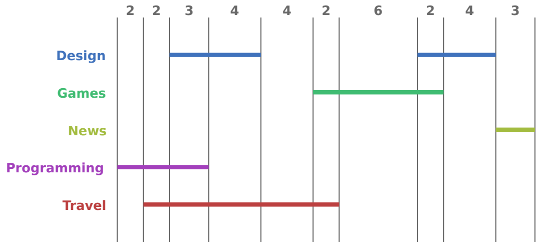
2 2 3 4 4 2 6 2 4 3
Design
Games
News
Programming
Travel

- LD-P&N

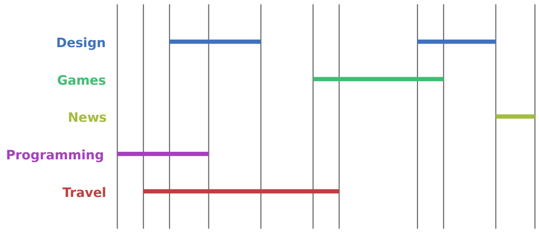
Design
Games
News
Programming
Travel

Question 5

- **Question:** Tick the check boxes where the total number of people interested in that topic is **greater than** the total number of people interested in **Relaxation**.
- **Check boxes to be ticked:** Hifi, iPhone, Media
- **Task Type:** Set comparison - More than.
- **SNAP Data Set Used:** 234169190
- ED-N

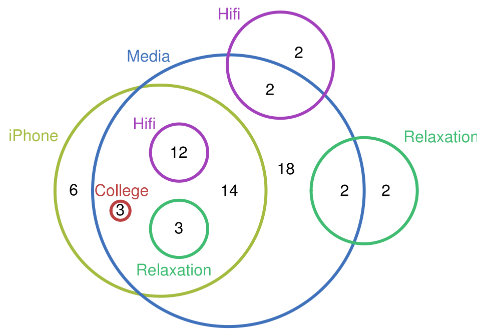

- ED-P

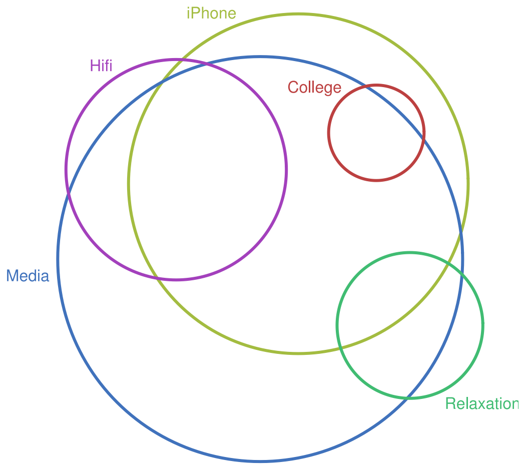

- ED-P&N

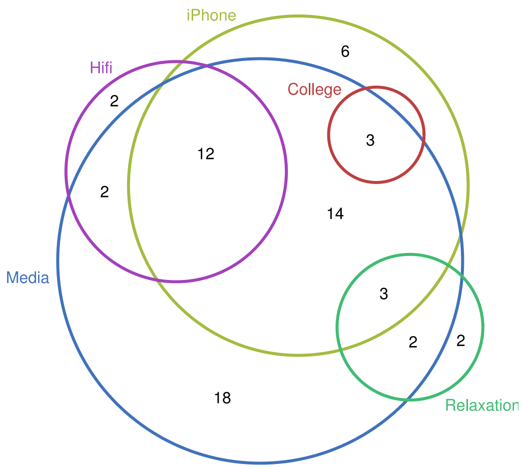
iPhone
6
Hifi
College
2
3
12
2
14
Media
3
2
2
18
Relaxation

- LD-N

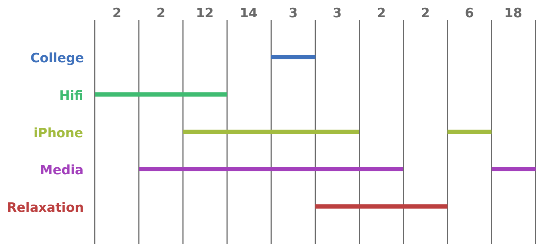
2
2
12
14
3
3
2
2
6
18
College
Hifi
iPhone
Media
Relaxation

- LD-P

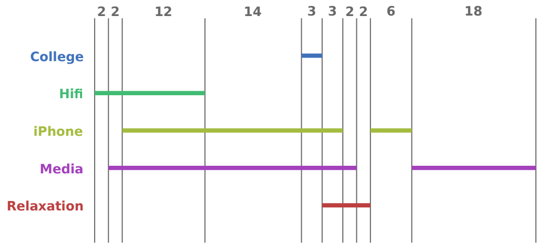
2 2
12
14
3
3
2 2
6
18
College
Hifi
iPhone
Media
Relaxation

- LD-P&N

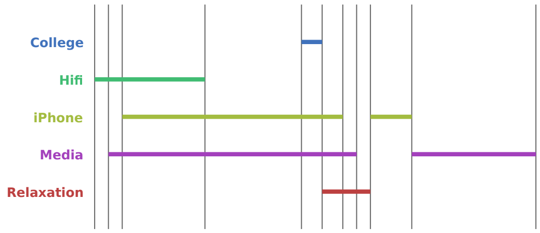
College
Hifi
iPhone
Media
Relaxation

Question 6

- **Question:** Tick the check boxes where the total number of people interested in that topic is **less than** the total number of people interested in **Stars**.
- **Check boxes to be ticked:** Games
- **Task Type:** Set comparison - Less than.
- **SNAP Data Set Used:** 46160500
- ED-N

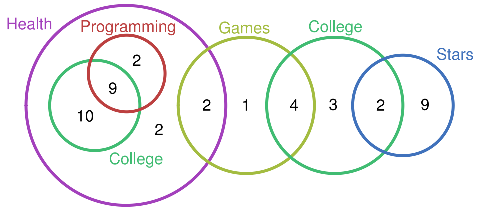

- ED-P

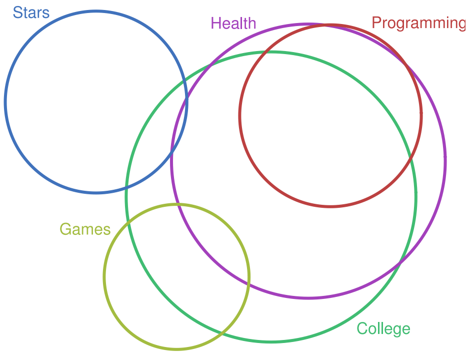

- ED-P&N

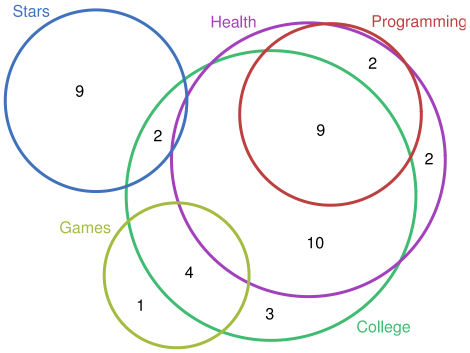

- LD-N

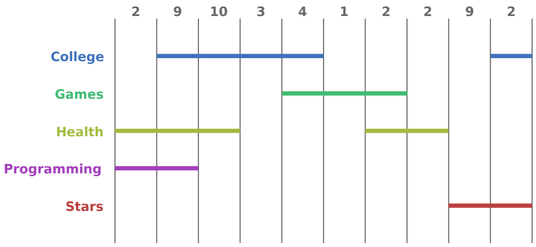

- LD-P

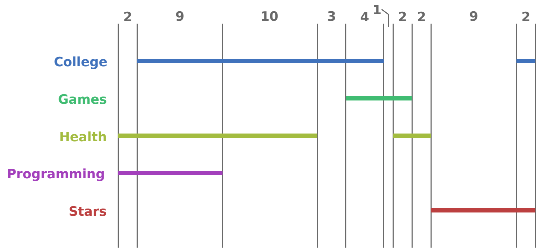

2
9
10
3
4
1
2
2
9
2
College
Games
Health
Programming
Stars

- LD-P&N

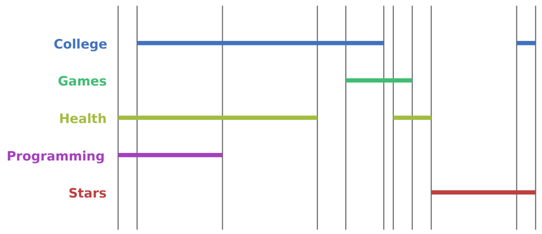

College
Games
Health
Programming
Stars

Question 7

- **Question:** Tick the check boxes where **more people** have exactly that combination of interests than **Internet only**.
- **Check boxes to be ticked:** Cars, Cars and Internet, Design and Programming, Programming
- **Task Type:** Intersection comparison - More than.
- **SNAP Data Set Used:** 87004822
- ED-N

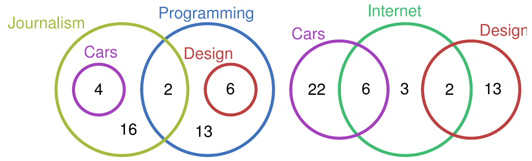

- ED-P

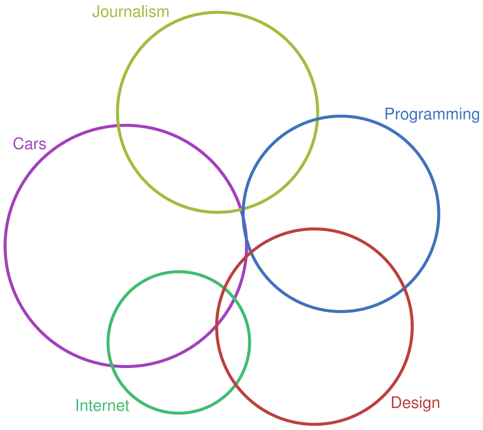

- ED-P&N

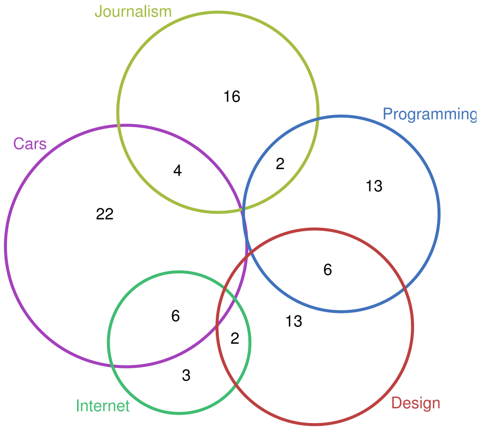

- LD-N

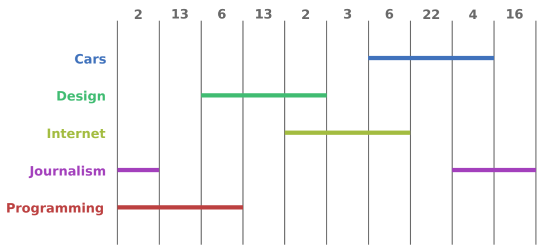
2
13
6
13
2
3
6
22
4
16
Cars
Design
Internet
Journalism
Programming

- LD-P

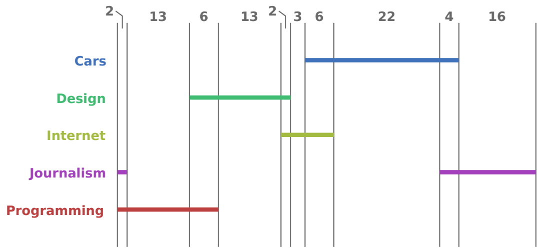
2
13
6
13
2
3
6
22
4
16
Cars
Design
Internet
Journalism
Programming

- LD-P&N

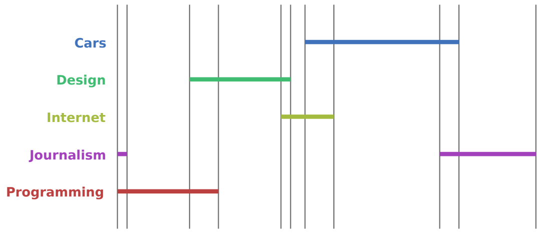
Cars
Design
Internet
Journalism
Programming

Question 8

- **Question:** Tick the check boxes where **more people** have exactly that combination of interests than **Web only**.
- **Check boxes to be ticked:** Cars, Health and Media, Media
- **Task Type:** Intersection comparison - Less than.
- **SNAP Data Set Used:** 14528221
- ED-N

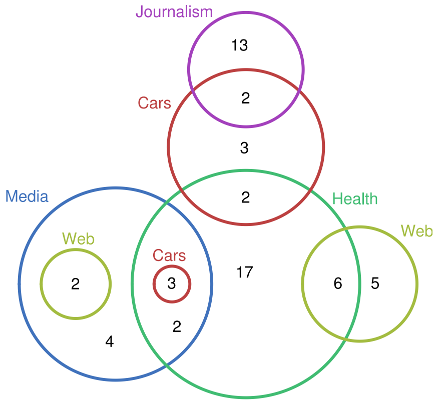

- ED-P

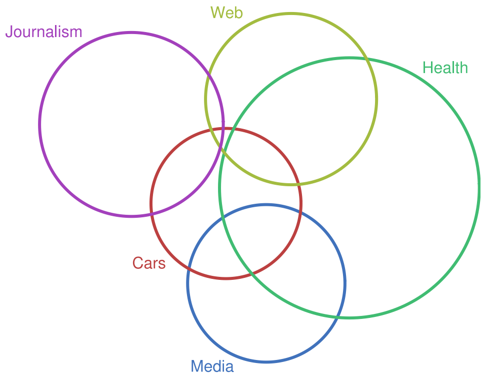

- ED-P&N

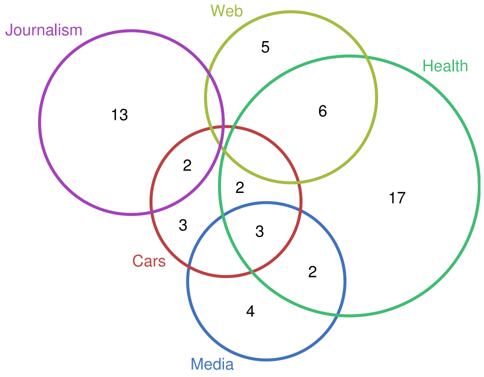

- LD-N

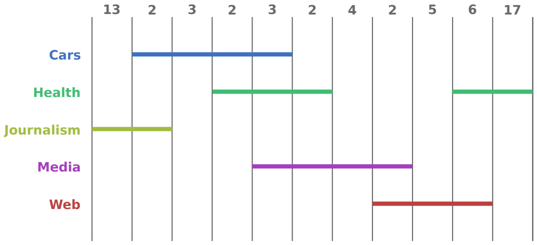
13 2 3 2 3 2 4 2 5 6 17
Cars
Health
Journalism
Media
Web

- LD-P

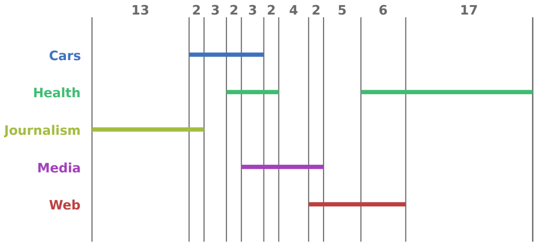
13 2 3 2 3 2 4 2 5 6 17
Cars
Health
Journalism
Media
Web

- LD-P&N

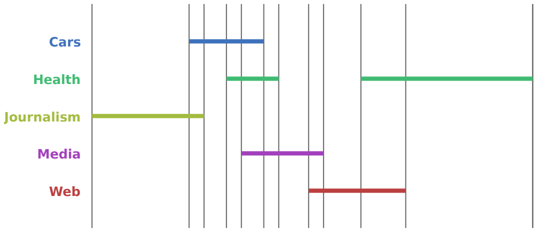
Cars
Health
Journalism
Media
Web

Question 9

- **Question:** Tick the check boxes where the total number of people interested in that topic is **greater than** the total number of people interested in **Games**.
- **Check boxes to be ticked:** Android, College, Economics, Hifi
- **Task Type:** Set comparison - More than.
- **SNAP Data Set Used:** 17561785
- ED-N

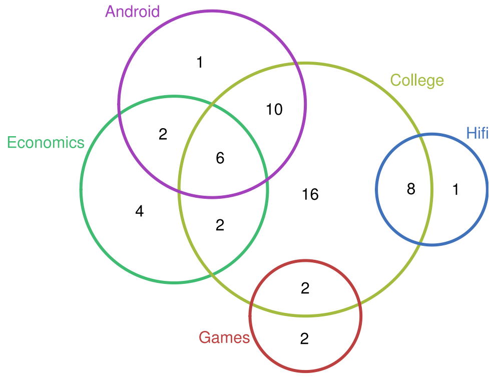

- ED-P

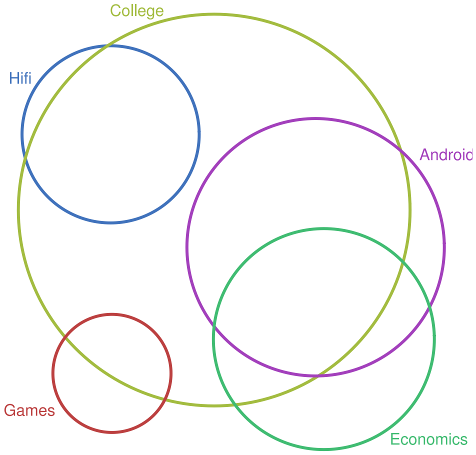

- ED-P&N

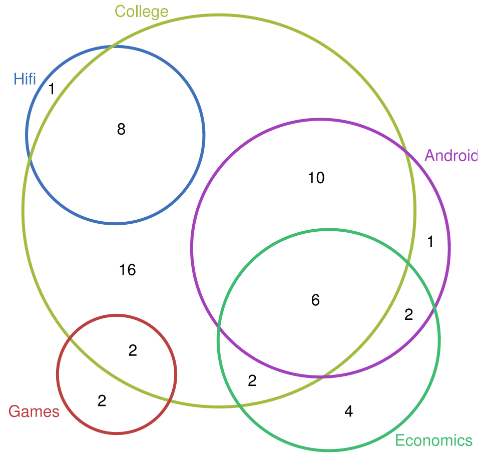
College
Hifi
Android
Games
Economics
1
8
10
1
16
6
2
2
2
2
4

- LD-N

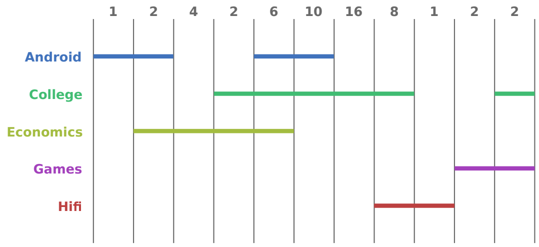
1 2 4 2 6 10 16 8 1 2 2
Android
College
Economics
Games
Hifi

- LD-P

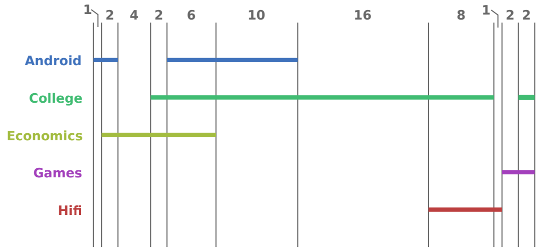
1 2 4 2 6 10 16 8 1 2 2
Android
College
Economics
Games
Hifi

- LD-P&N

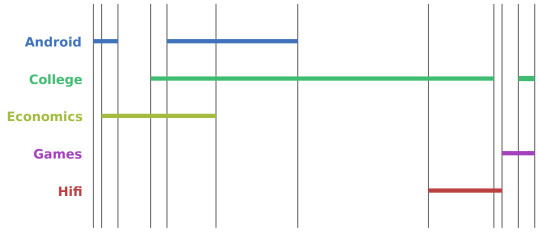
Android
College
Economics
Games
Hifi

Question 10

- **Question:** Tick the check boxes where the total number of people interested in that topic is **less than** the total number of people interested in **Camping**.
- **Check boxes to be ticked:** None of the above
- **Task Type:** Set comparison - Less than.
- **SNAP Data Set Used:** 105150583
- ED-N

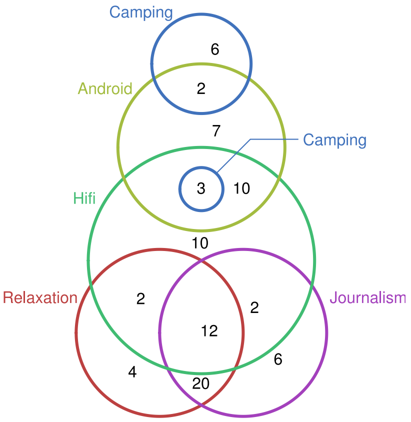

- ED-P

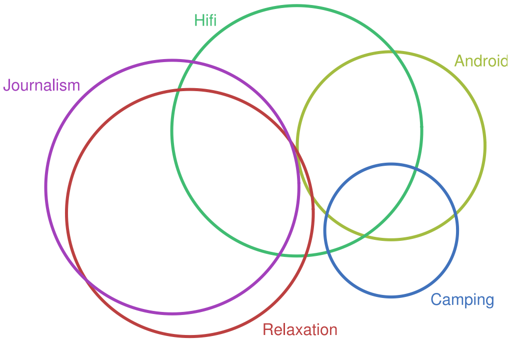

- ED-P&N

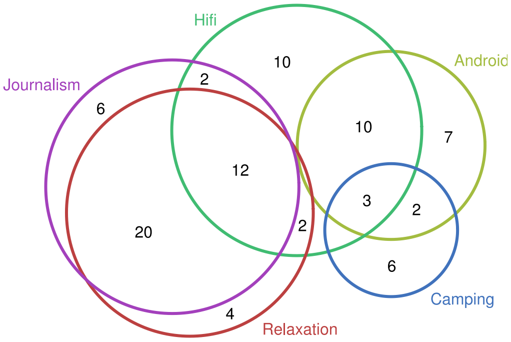

- LD-N

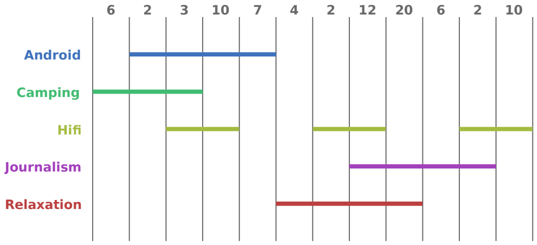
6
2
3
10
7
4
2
12
20
6
2
10
Android
Camping
Hifi
Journalism
Relaxation

- LD-P

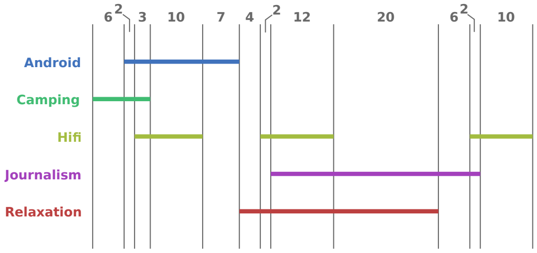
6
2
3
10
7
4
2
12
20
6
2
10
Android
Camping
Hifi
Journalism
Relaxation

- LD-P&N

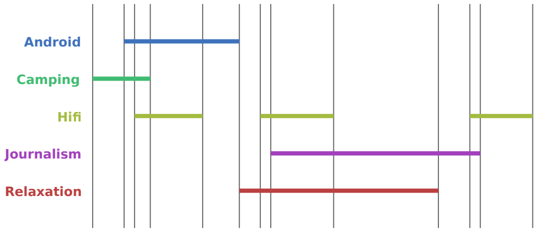
Android
Camping
Hifi
Journalism
Relaxation

Question 11

- **Question:** Tick the check boxes where **more people** have exactly that combination of interests than **Technology only**.
- **Check boxes to be ticked:** None of the above
- **Task Type:** Intersection comparison - More than.
- **SNAP Data Set Used:** 18534908
- ED-N

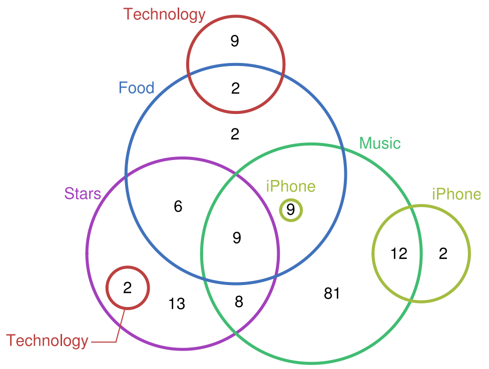

- ED-P

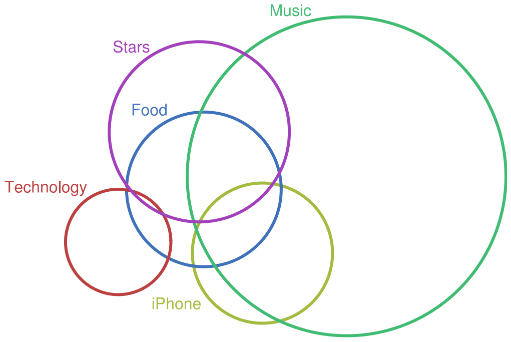

- ED-P&N

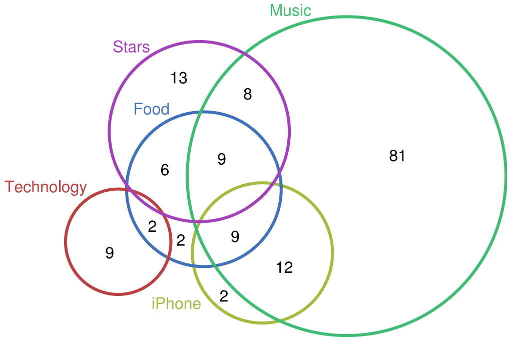

- LD-N

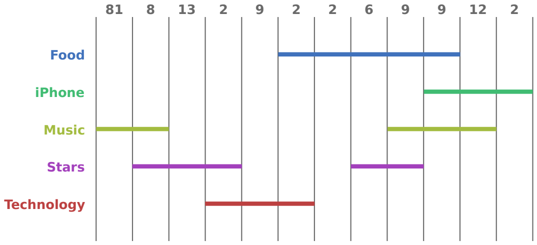
81 8 13 2 9 2 2 6 9 9 12 2
Food
iPhone
Music
Stars
Technology

- LD-P

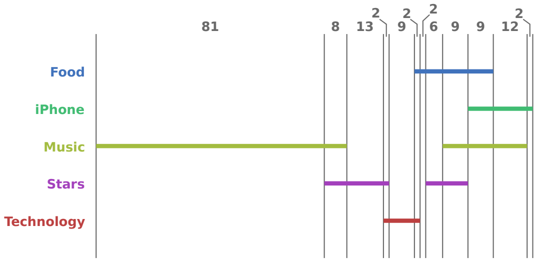
2 2 2 2
81 8 13 9 6 9 9 12
Food
iPhone
Music
Stars
Technology

- LD-P&N

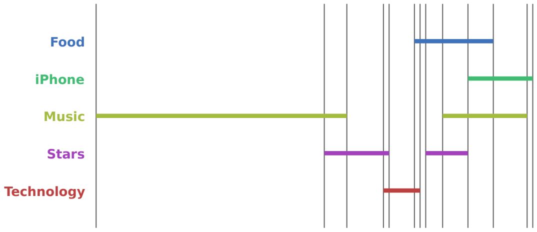
Food
iPhone
Music
Stars
Technology

Question 12

- **Question:** Tick the check boxes where **more people** have exactly that combination of interests than **Android and Games only**.
- **Check boxes to be ticked:** Android and Design, Android and Stars
- **Task Type:** Intersection comparison - Less than.
- **SNAP Data Set Used:** 59588845
- ED-N

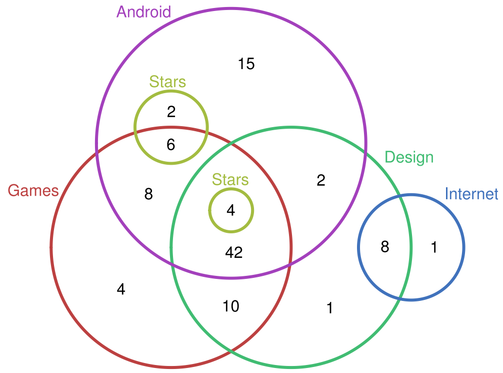

- ED-P

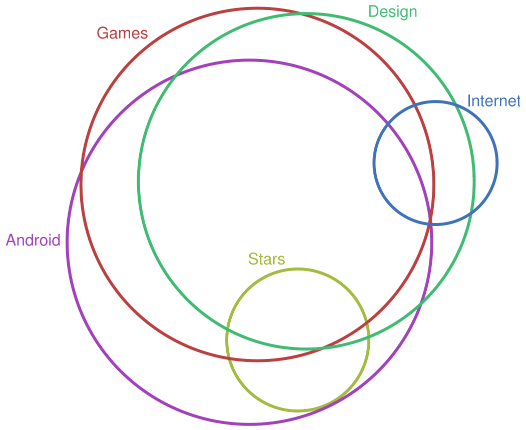

- ED-P&N

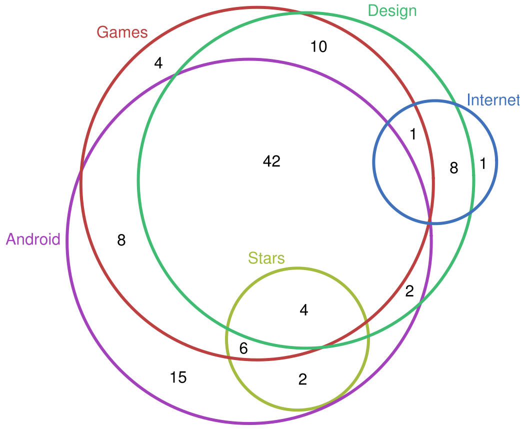
Games
Design
Internet
Android
Stars
4
10
1
42
8
1
8
2
4
6
15
2

- LD-N

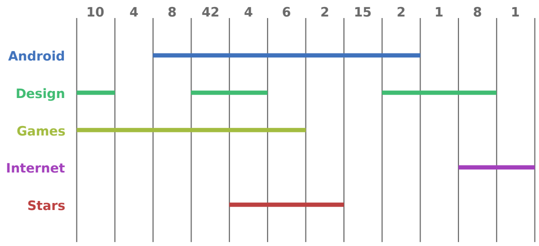
10 4 8 42 4 6 2 15 2 1 8 1
Android
Design
Games
Internet
Stars

- LD-P

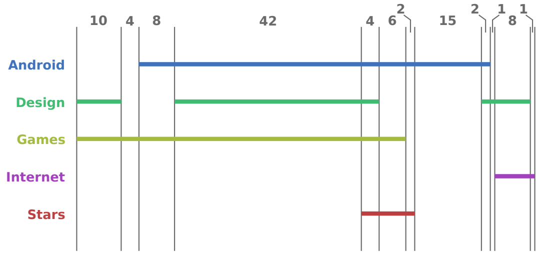
10 4 8 42 4 6 2 15 2 1 8 1
Android
Design
Games
Internet
Stars

- LD-P&N

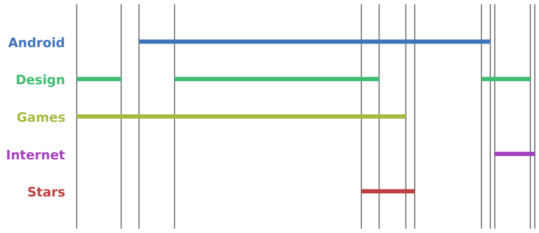
Android
Design
Games
Internet
Stars

Question 13

- **Question:** Tick the check boxes where the total number of people interested in that topic is **greater than** the total number of people interested in **Relaxation**.
- **Check boxes to be ticked:** Food, iPhone, Travel
- **Task Type:** Set comparison - More than.
- **SNAP Data Set Used:** 64441390
- ED-N

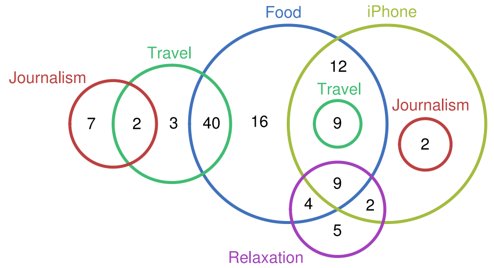

- ED-P

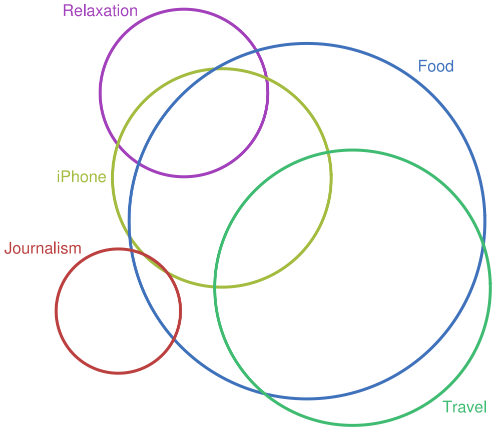

- ED-P&N

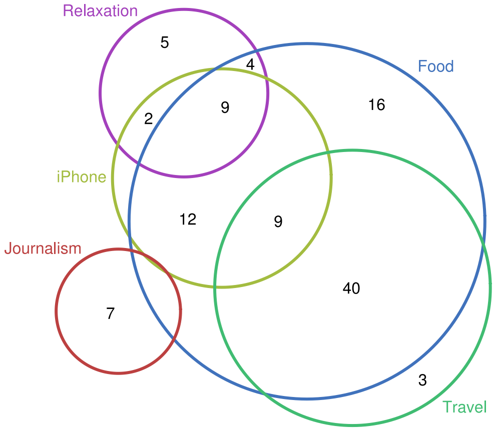

- LD-N

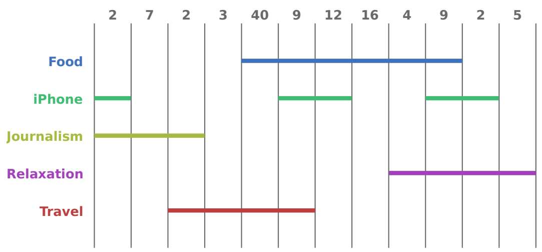
2 7 2 3 40 9 12 16 4 9 2 5
Food
iPhone
Journalism
Relaxation
Travel

- LD-P

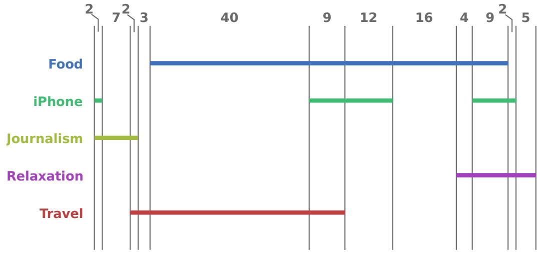
2 2 2
7 3 40 9 12 16 4 9 5
Food
iPhone
Journalism
Relaxation
Travel

- LD-P&N

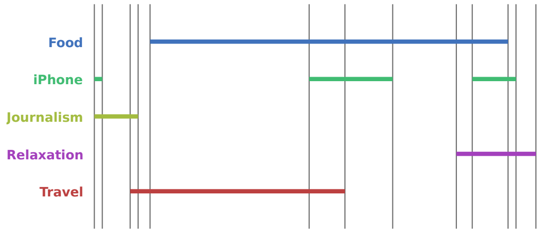
Food
iPhone
Journalism
Relaxation
Travel

Question 14

- **Question:** Tick the check boxes where the total number of people interested in that topic is **less than** the total number of people interested in **Stars**.
- **Check boxes to be ticked:** Music
- **Task Type:** Set comparison - Less than.
- **SNAP Data Set Used:** 190696559
- ED-N

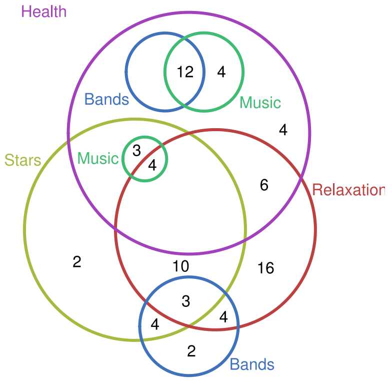

- ED-P

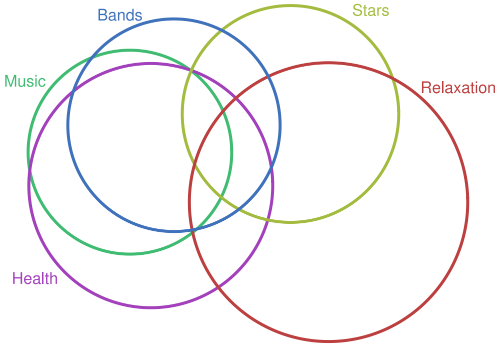

- ED-P&N

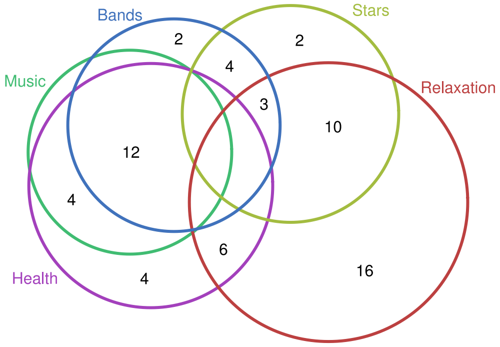

- LD-N

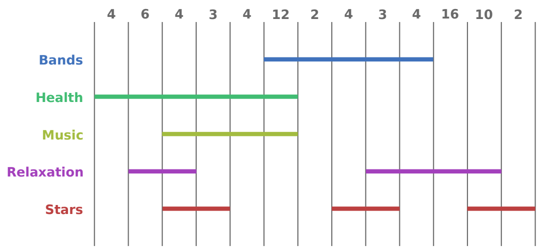

4 6 4 3 4 12 2 4 3 4 16 10 2
Bands
Health
Music
Relaxation
Stars

- LD-P

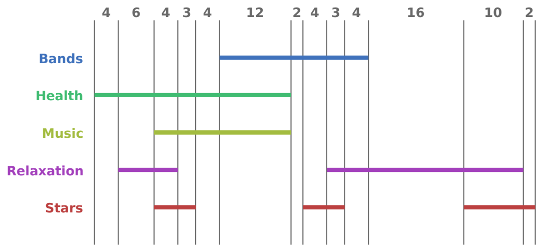

4 6 4 3 4 12 2 4 3 4 16 10 2
Bands
Health
Music
Relaxation
Stars

- LD-P&N

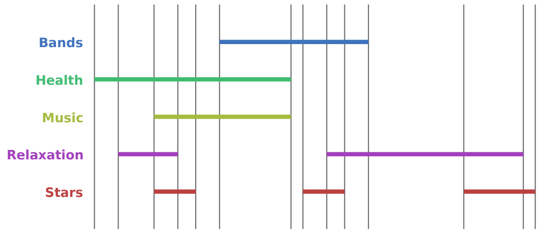

Bands
Health
Music
Relaxation
Stars

Question 15

- **Question:** Tick the check boxes where **more people** have exactly that combination of interests than **Hifi, Movies and Technology only**.
- **Check boxes to be ticked:** Hifi, Journalism and Technology, Hifi and Journalism and Technology, Hifi and Relaxation and Technology
- **Task Type:** Intersection comparison - More than.
- **SNAP Data Set Used:** 22863638
- ED-N

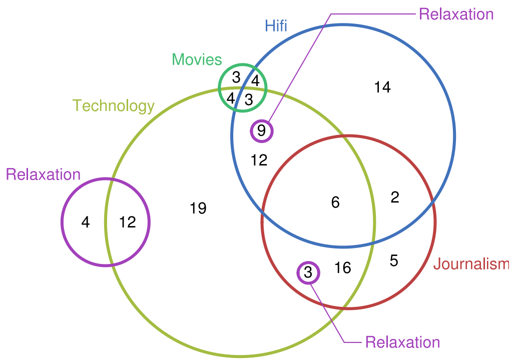

- ED-P

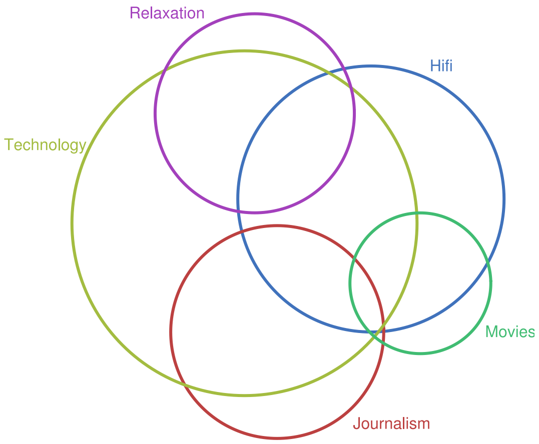

- ED-P&N

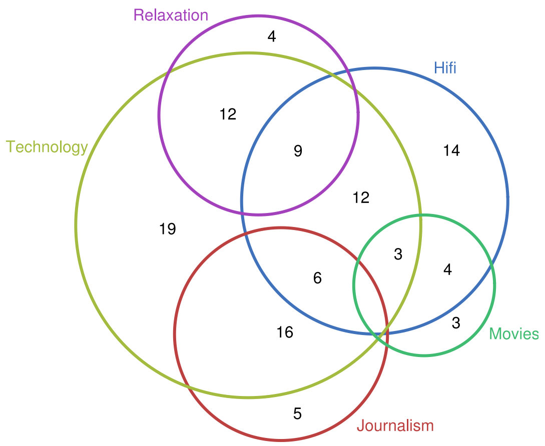
Relaxation
Hifi
Technology
Movies
Journalism
4
12
9
14
12
19
3
4
6
3
16
5

- LD-N

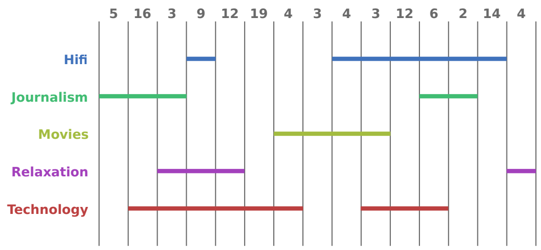
5 16 3 9 12 19 4 3 4 3 12 6 2 14 4
Hifi
Journalism
Movies
Relaxation
Technology

- LD-P

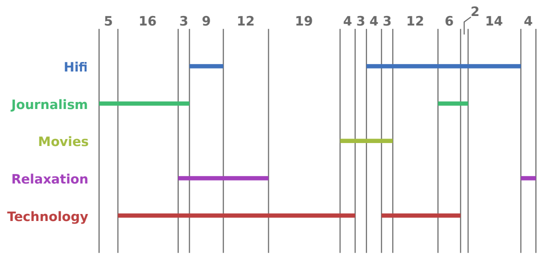
5 16 3 9 12 19 4 3 4 3 12 6 2 14 4
Hifi
Journalism
Movies
Relaxation
Technology

- LD-P&N

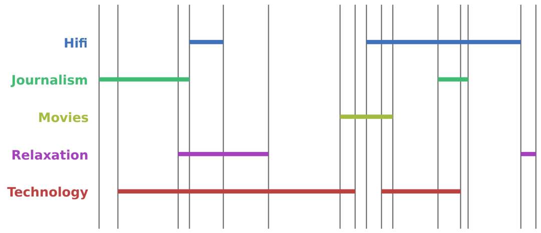
Hifi
Journalism
Movies
Relaxation
Technology

Question 16

- **Question:** Tick the check boxes where **more people** have exactly that combination of interests than **Bands and Web only**.
- **Check boxes to be ticked:** Bands and Food
- **Task Type:** Intersection comparison - Less than.
- **SNAP Data Set Used:** 15864197
- ED-N

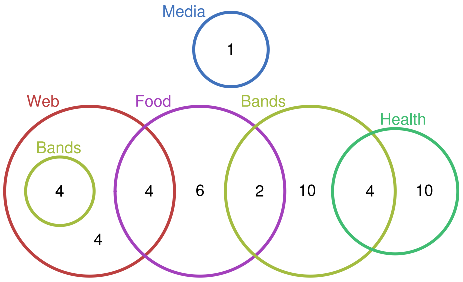

- ED-P

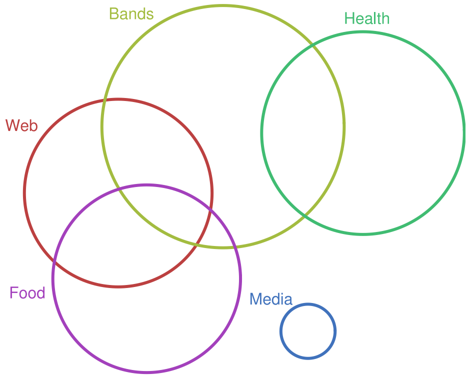

- ED-P&N

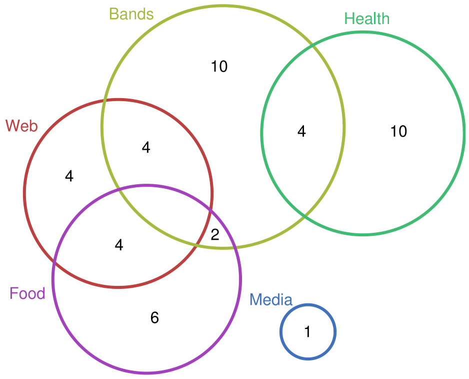

- LD-N

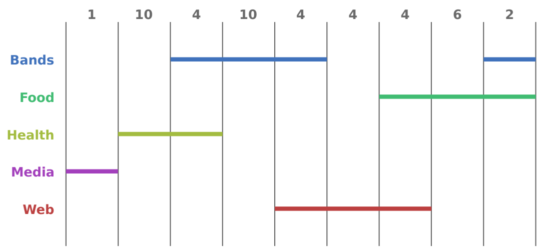

1
10
4
10
4
4
4
6
2
Bands
Food
Health
Media
Web

- LD-P

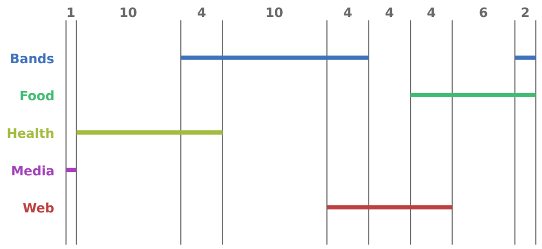

1
10
4
10
4
4
4
6
2
Bands
Food
Health
Media
Web

- LD-P&N

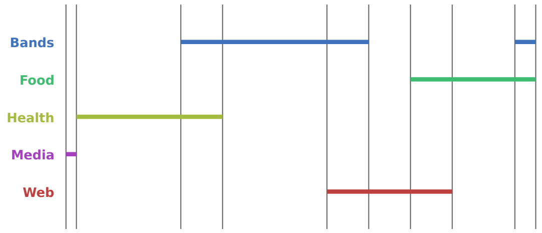

Bands
Food
Health
Media
Web

Question 17

- **Question:** Tick the check boxes where the total number of people interested in that topic is **greater than** the total number of people interested in **Books**.
- **Check boxes to be ticked:** Hifi, iPhone
- **Task Type:** Set comparison - More than.
- **SNAP Data Set Used:** 1046661
- ED-N

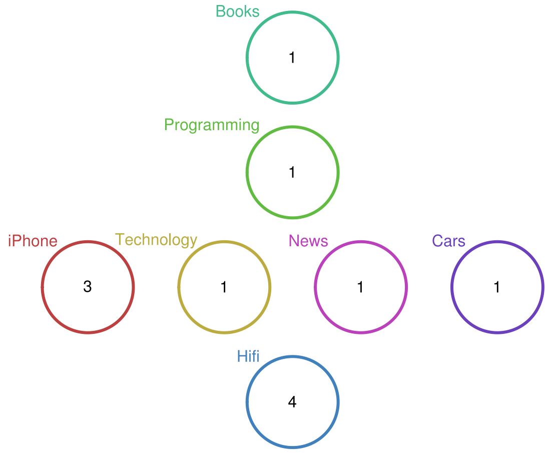

- ED-P

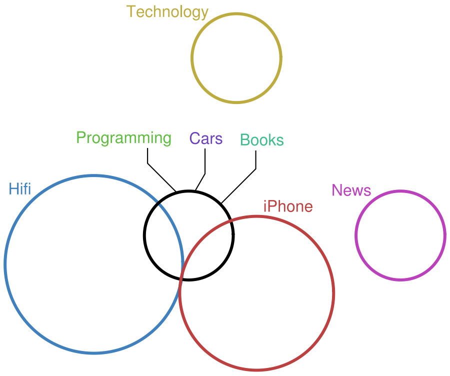

- ED-P&N

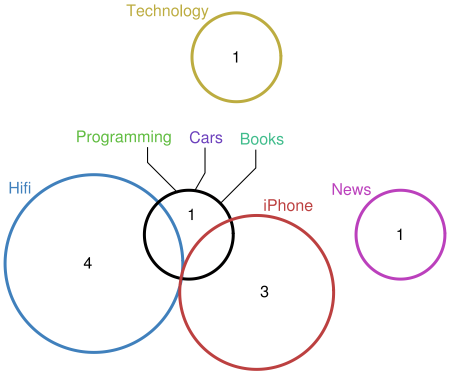

- LD-N

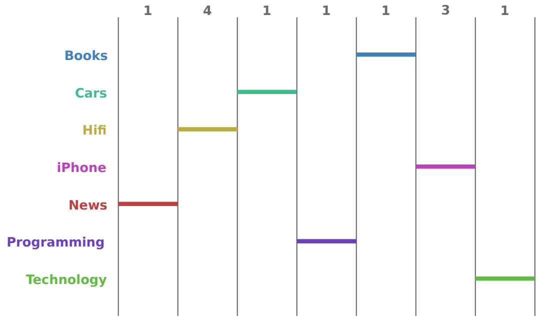
1
4
1
1
1
3
1
Books
Cars
Hifi
iPhone
News
Programming
Technology

- LD-P

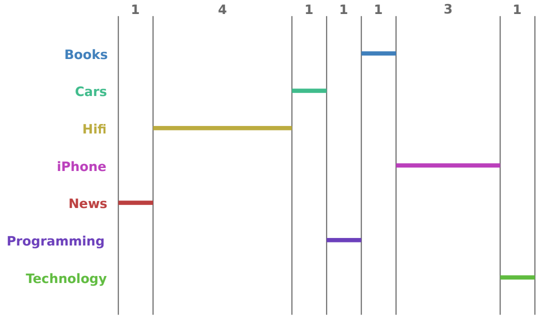
1
4
1
1
1
3
1
Books
Cars
Hifi
iPhone
News
Programming
Technology

- LD-P&N

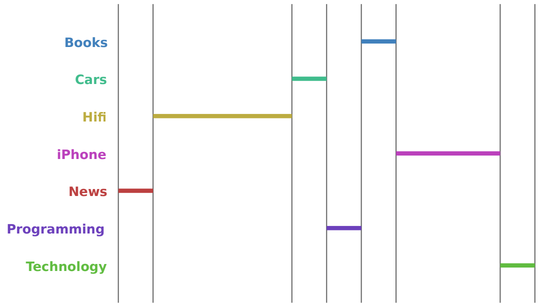
Books
Cars
Hifi
iPhone
News
Programming
Technology

Question 18

- **Question:** Tick the check boxes where the total number of people interested in that topic is **less than** the total number of people interested in **Journalism**.
- **Check boxes to be ticked:** Bands, Design, Music, News
- **Task Type:** Set comparison - Less than.
- **SNAP Data Set Used:** 124296976
- ED-N

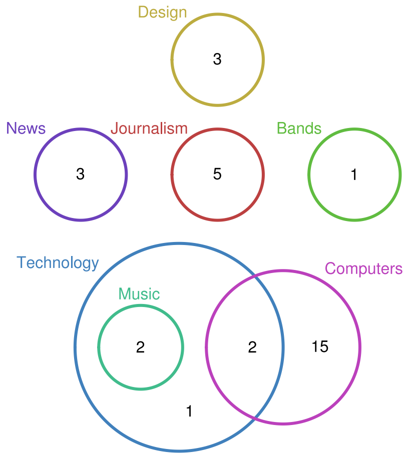

- ED-P

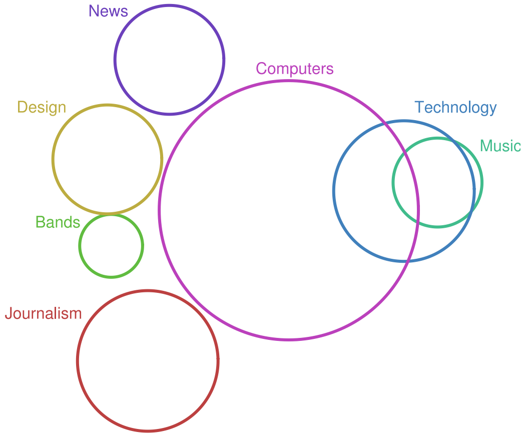

- ED-P&N

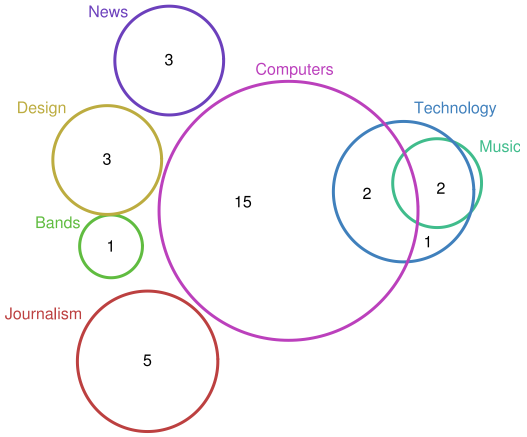
News
3
Computers
Design
3
Technology
Music
15
2
2
Bands
1
1
Journalism
5

- LD-N

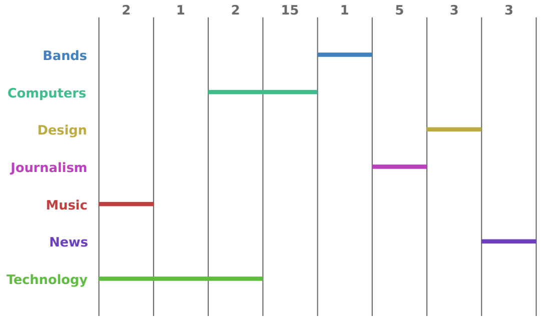
2
1
2
15
1
5
3
3
Bands
Computers
Design
Journalism
Music
News
Technology

- LD-P

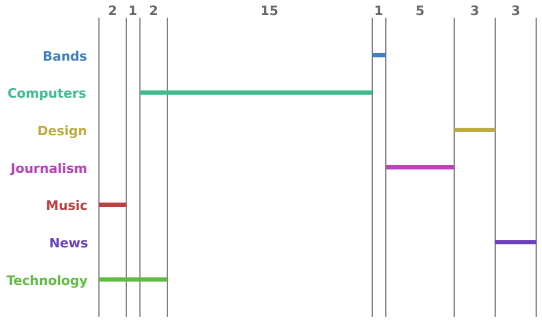
2
1
2
15
1
5
3
3
Bands
Computers
Design
Journalism
Music
News
Technology

- LD-P&N

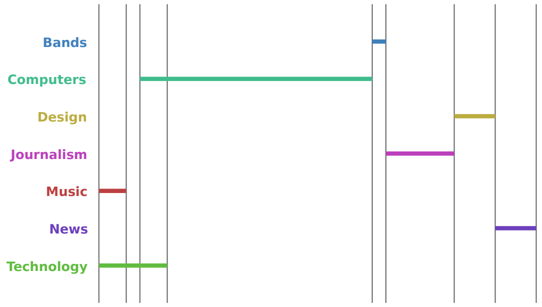
Bands
Computers
Design
Journalism
Music
News
Technology

Question 19

- **Question:** Tick the check boxes where **more people** have exactly that combination of interests than **Camping, Games and Relaxation only**.
- **Check boxes to be ticked:** News, Hifi, Books
- **Task Type:** Intersection comparison - More than.
- **SNAP Data Set Used:** 13747362
- ED-N

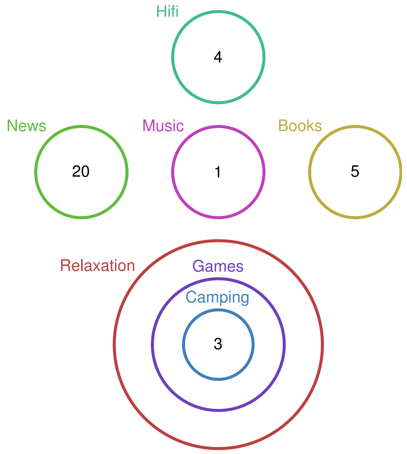

- ED-P

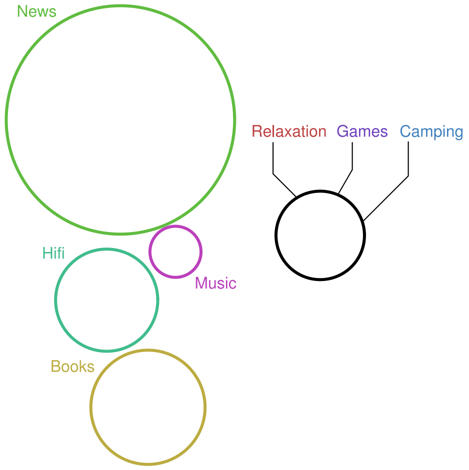

- ED-P&N

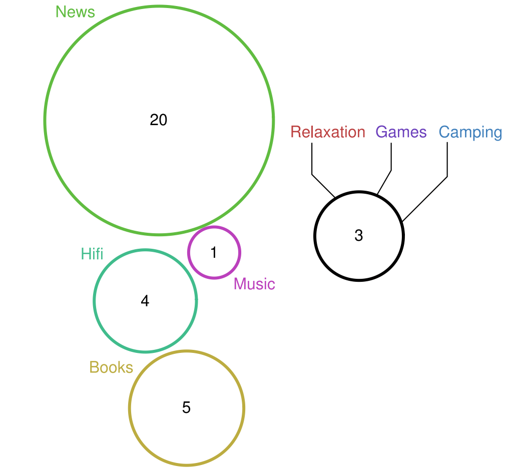
News
20
Relaxation
Games
Camping
3
Hifi
1
Music
4
Books
5

- LD-N

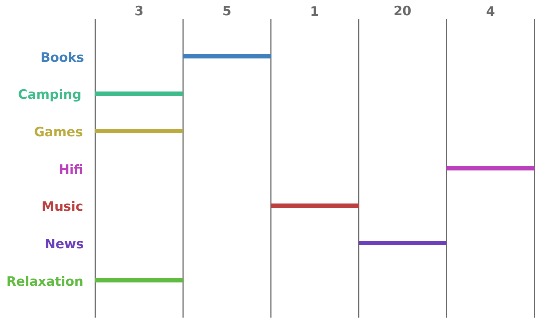
3
5
1
20
4
Books
Camping
Games
Hifi
Music
News
Relaxation

- LD-P

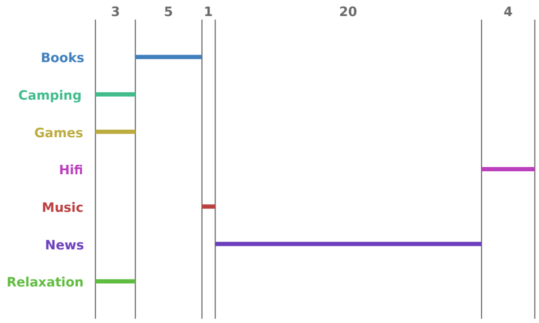
3
5
1
20
4
Books
Camping
Games
Hifi
Music
News
Relaxation

- LD-P&N

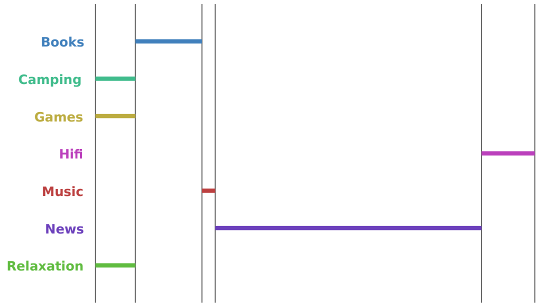
Books
Camping
Games
Hifi
Music
News
Relaxation

Question 20

- **Question:** Tick the check boxes where **more people** have exactly that combination of interests than **iPhone only**.
- **Check boxes to be ticked:** Movies
- **Task Type:** Intersection comparison - Less than.
- **SNAP Data Set Used:** 23790454
- ED-N

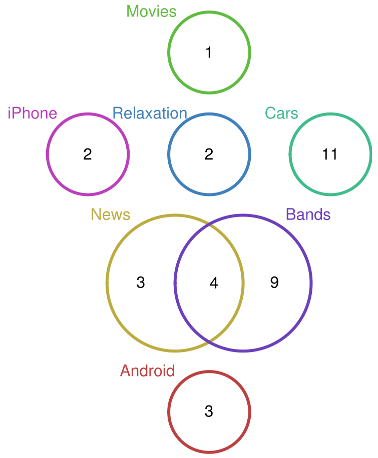

- ED-P

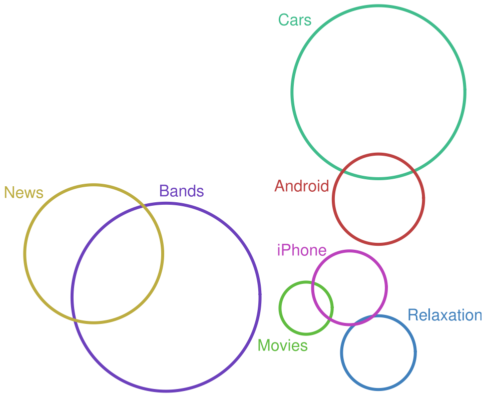

- ED-P&N

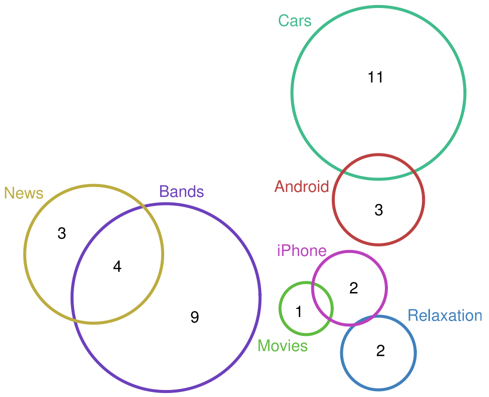
Cars
11
Android
3
News
Bands
3
4
9
iPhone
2
1
Relaxation
Movies
2

- LD-N

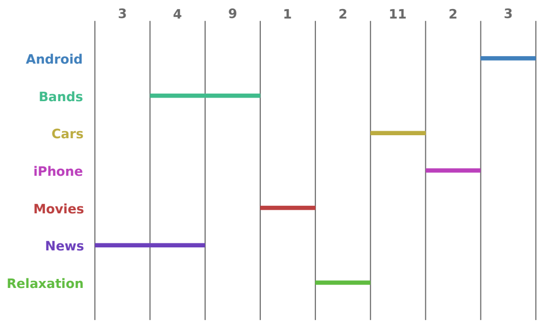
3 4 9 1 2 11 2 3
Android
Bands
Cars
iPhone
Movies
News
Relaxation

- LD-P

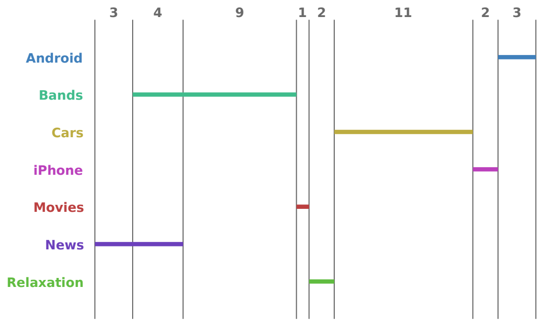
3 4 9 1 2 11 2 3
Android
Bands
Cars
iPhone
Movies
News
Relaxation

- LD-P&N

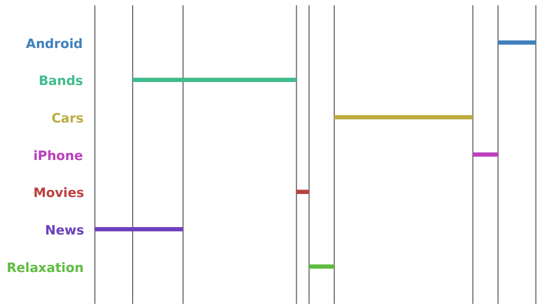
Android
Bands
Cars
iPhone
Movies
News
Relaxation

Question 21

- **Question:** Tick the check boxes where the total number of people interested in that topic is **greater than** the total number of people interested in **Bands**.
- **Check boxes to be ticked:** Games, Music
- **Task Type:** Set comparison - More than.
- **SNAP Data Set Used:** 13274152
- ED-N

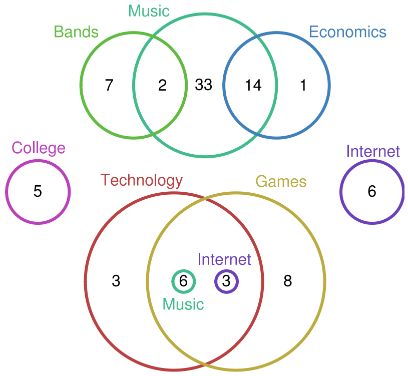

- ED-P

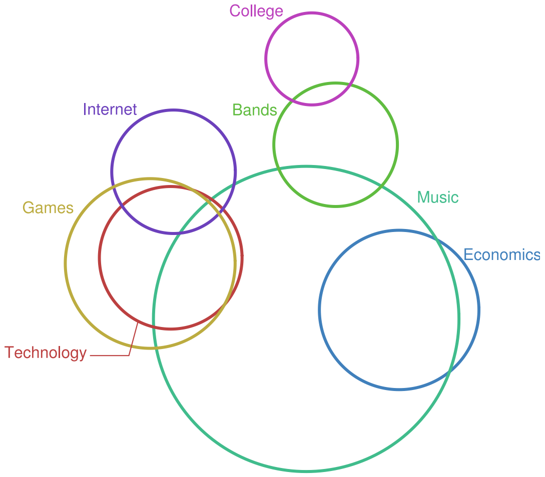

- ED-P&N

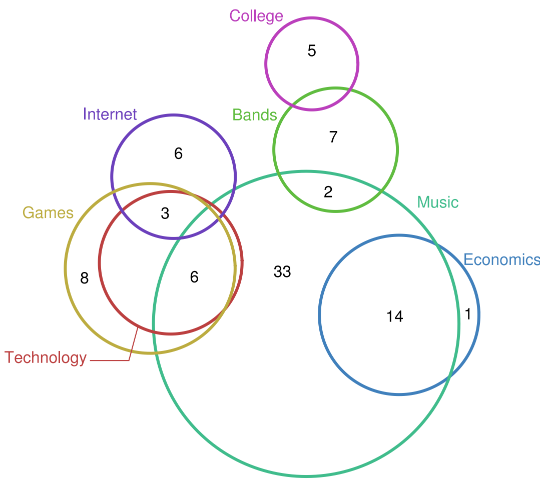
College
5
Internet
Bands
6
7
2
Music
Games
3
Economics
8
6
33
14
1
Technology

- LD-N

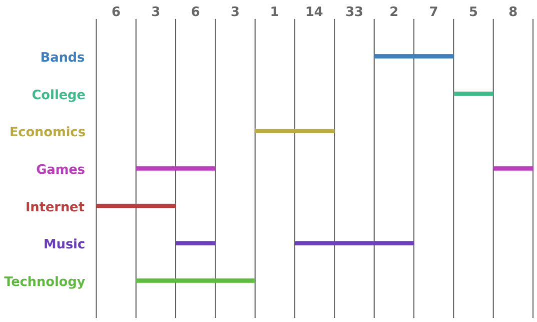
6 3 6 3 1 14 33 2 7 5 8
Bands
College
Economics
Games
Internet
Music
Technology

- LD-P

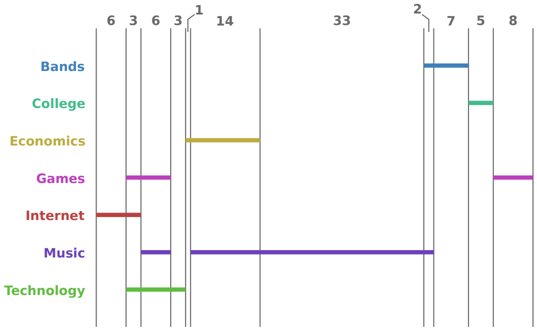
6 3 6 3 1 14 33 2 7 5 8
Bands
College
Economics
Games
Internet
Music
Technology

- LD-P&N

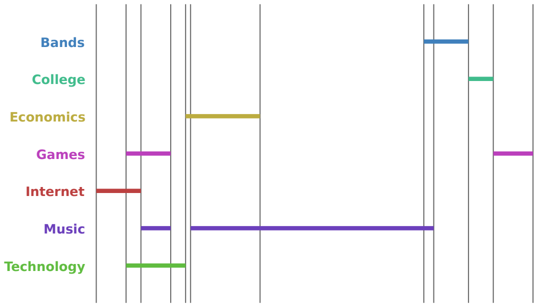
Bands
College
Economics
Games
Internet
Music
Technology

Question 22

- **Question:** Tick the check boxes where the total number of people interested in that topic is **less than** the total number of people interested in **Design**.
- **Check boxes to be ticked:** News
- **Task Type:** Set comparison - Less than.
- **SNAP Data Set Used:** 16834201
- ED-N

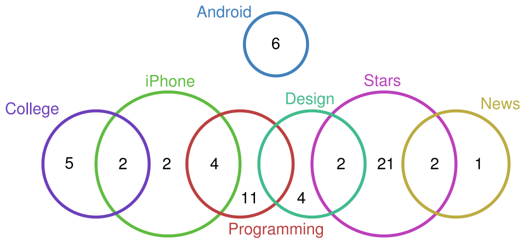

- ED-P

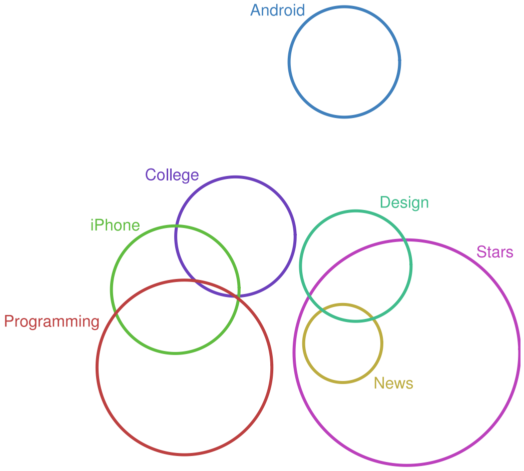

- ED-P&N

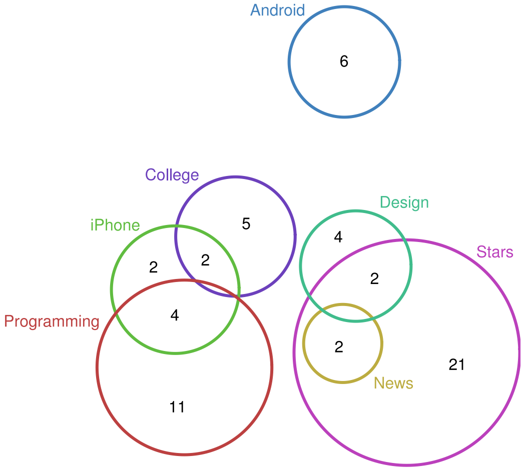

- LD-N

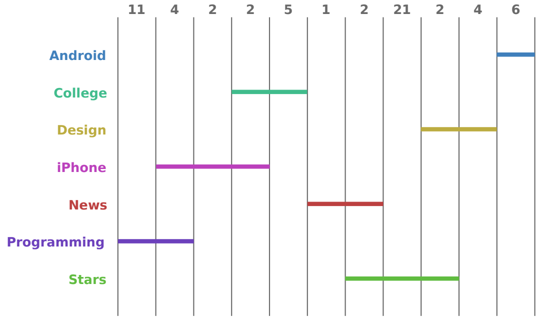
11
4
2
2
5
1
2
21
2
4
6
Android
College
Design
iPhone
News
Programming
Stars

- LD-P

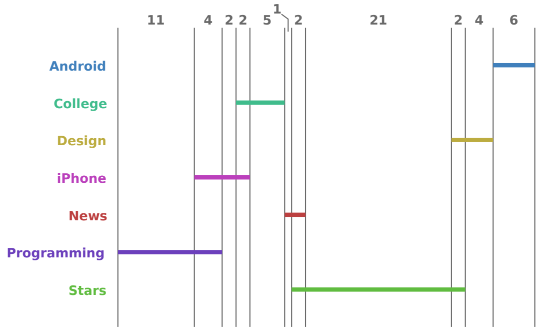
1
11
4
2
2
5
2
21
2
4
6
Android
College
Design
iPhone
News
Programming
Stars

- LD-P&N

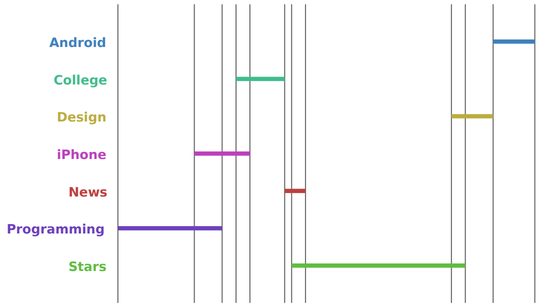
Android
College
Design
iPhone
News
Programming
Stars

Question 23

- **Question:** Tick the check boxes where **more people** have exactly that combination of interests than **Computers, Design and Travel only**.
- **Check boxes to be ticked:** Internet and Travel
- **Task Type:** Intersection comparison - More than.
- **SNAP Data Set Used:** 29514951
- ED-N

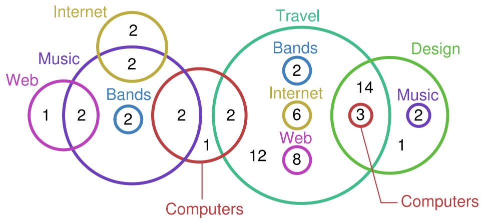

- ED-P

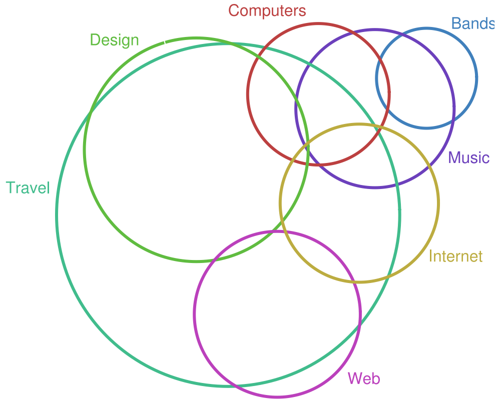

- ED-P&N

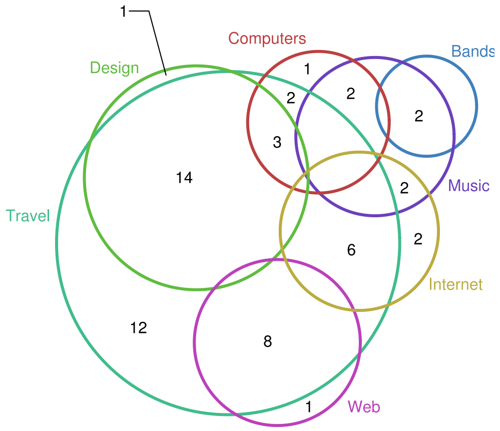

- LD-N

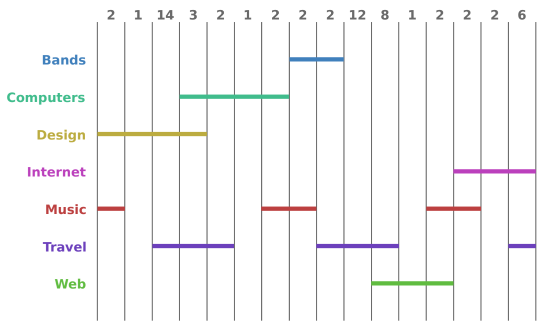
2 1 14 3 2 1 2 2 2 12 8 1 2 2 2 6
Bands
Computers
Design
Internet
Music
Travel
Web

- LD-P

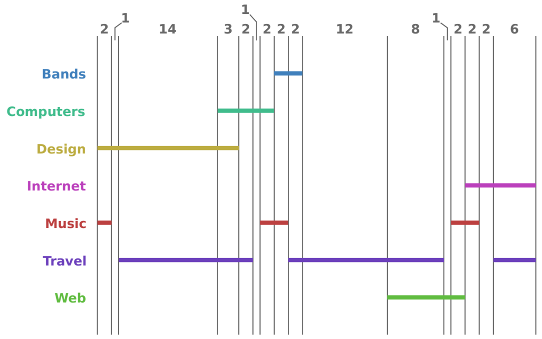
2 1 14 3 2 1 2 2 2 12 8 1 2 2 2 6
Bands
Computers
Design
Internet
Music
Travel
Web

- LD-P&N

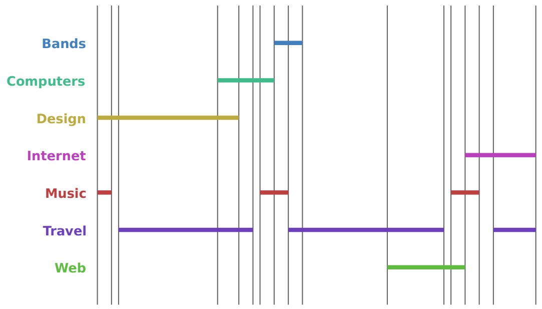
Bands
Computers
Design
Internet
Music
Travel
Web

Question 24

- **Question:** Tick the check boxes where **more people** have exactly that combination of interests than **Camping only**.
- **Check boxes to be ticked:** Camping, Food, Economics
- **Task Type:** Intersection comparison - Less than.
- **SNAP Data Set Used:** 17767841
- ED-N

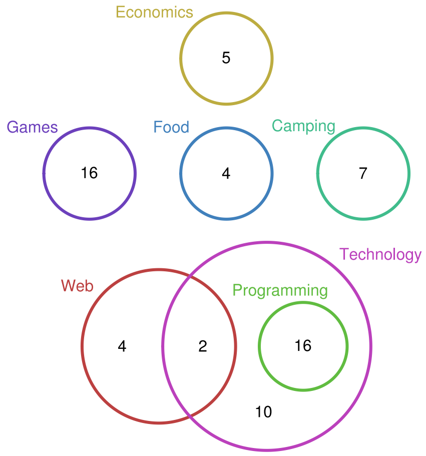

- ED-P

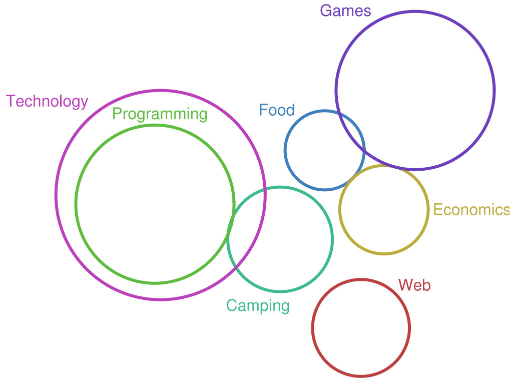

- ED-P&N

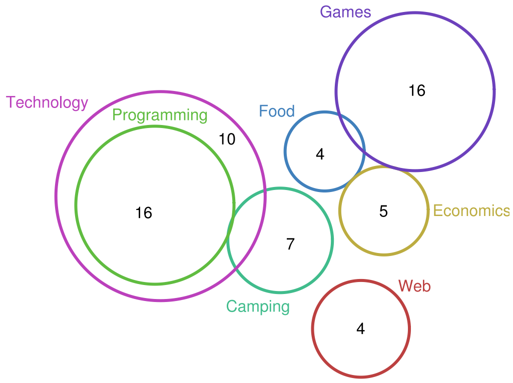

- LD-N

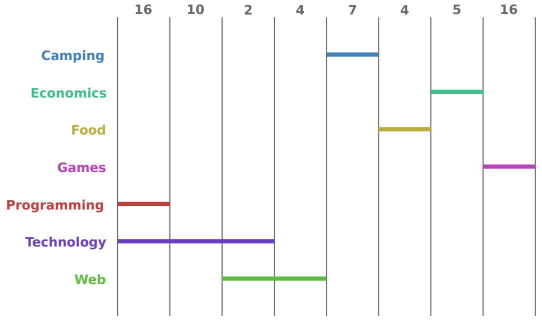
16
10
2
4
7
4
5
16
Camping
Economics
Food
Games
Programming
Technology
Web

- LD-P

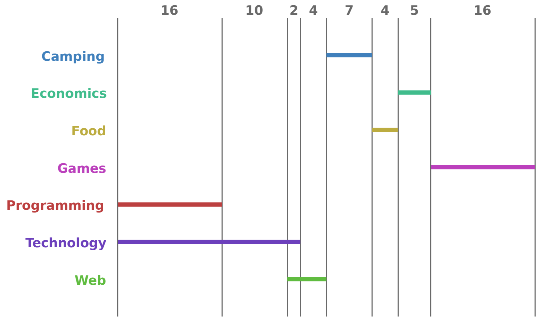
16
10
2
4
7
4
5
16
Camping
Economics
Food
Games
Programming
Technology
Web

- LD-P&N

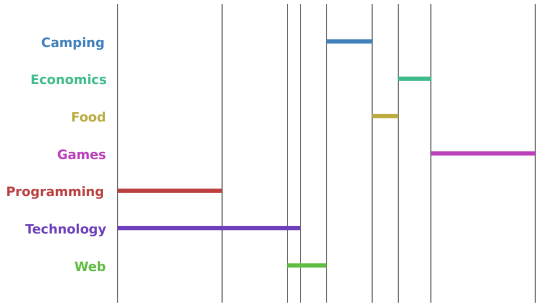
Camping
Economics
Food
Games
Programming
Technology
Web

Question 25

- **Question:** Tick the check boxes where the total number of people interested in that topic is **greater than** the total number of people interested in **Health**.
- **Check boxes to be ticked:** Economics, Internet, Media
- **Task Type:** Set comparison - More than.
- **SNAP Data Set Used:** 22121627
- ED-N

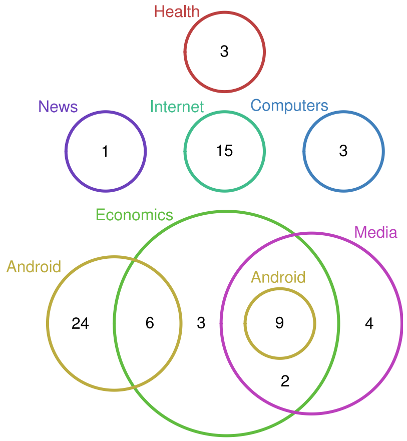

- ED-P

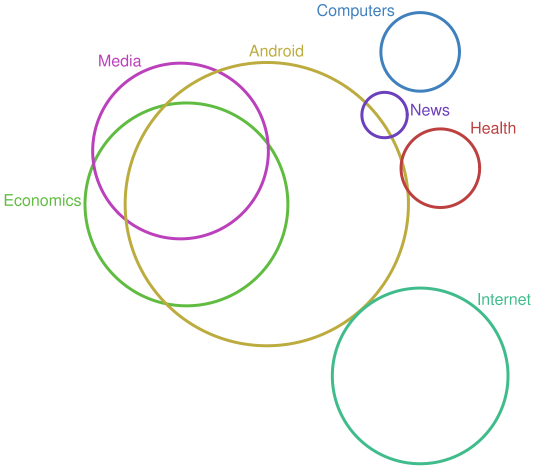

- ED-P&N

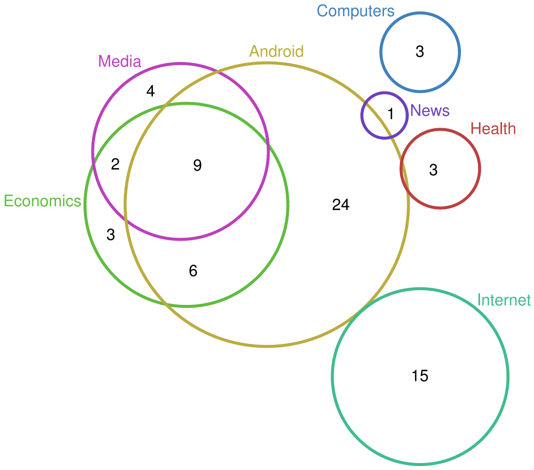
Computers
3
Media
Android
4
1
News
Health
2
9
3
Economics
24
3
6
Internet
15

- LD-N

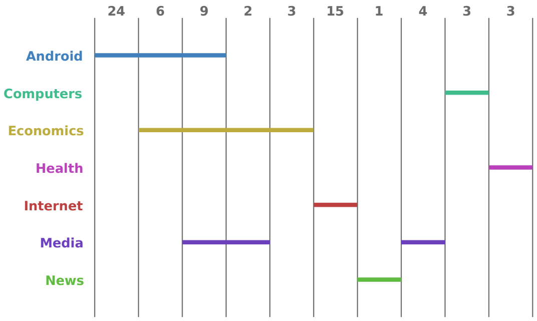
24 6 9 2 3 15 1 4 3 3
Android
Computers
Economics
Health
Internet
Media
News

- LD-P

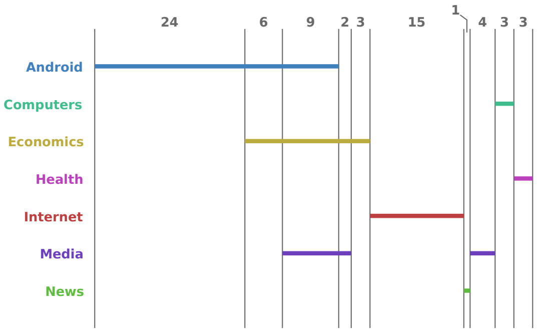
24 6 9 2 3 15 1 4 3 3
Android
Computers
Economics
Health
Internet
Media
News

- LD-P&N

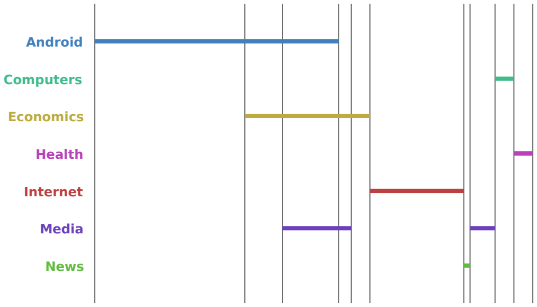
Android
Computers
Economics
Health
Internet
Media
News

Question 26

- **Question:** Tick the check boxes where the total number of people interested in that topic is **less than** the total number of people interested in **Health**.
- **Check boxes to be ticked:** Bands, Games, Movies, Programming
- **Task Type:** Set comparison - Less than.
- **SNAP Data Set Used:** 2029971
- ED-N

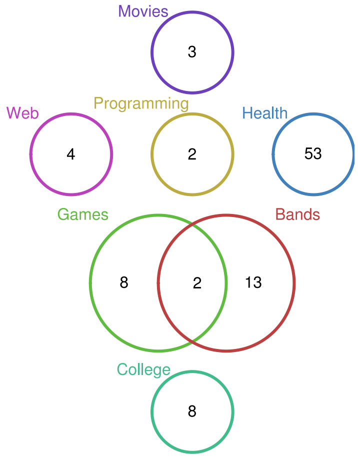

- ED-P

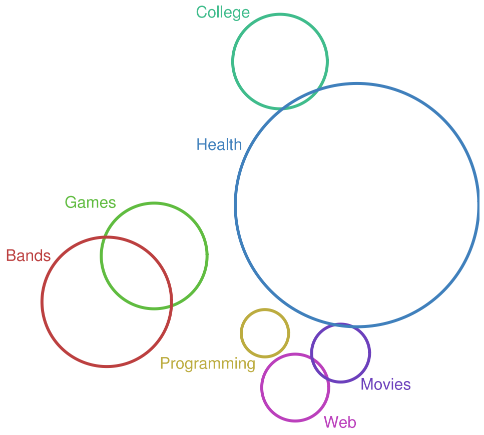

- ED-P&N

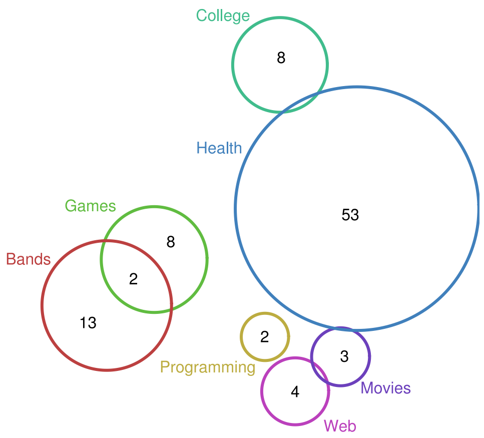
College
8
Health
53
Games
8
Bands
2
13
2
Programming
3
Movies
4
Web

- LD-N

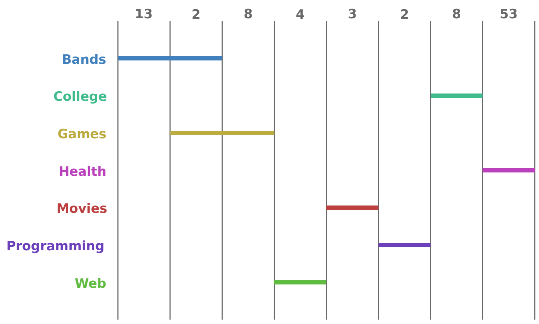
13 2 8 4 3 2 8 53
Bands
College
Games
Health
Movies
Programming
Web

- LD-P

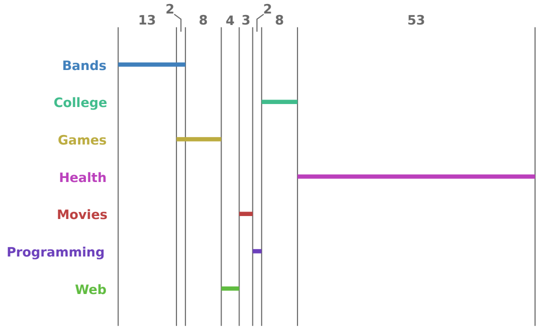
13 2 8 4 3 2 8 53
Bands
College
Games
Health
Movies
Programming
Web

- LD-P&N

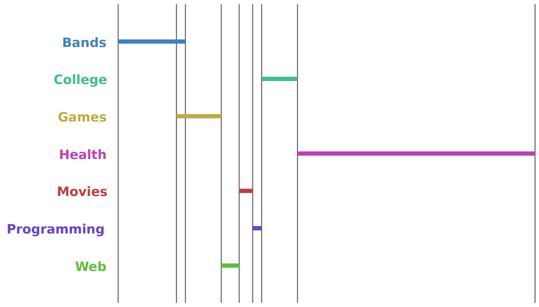
Bands
College
Games
Health
Movies
Programming
Web

Question 27

- **Question:** Tick the check boxes where **more people** have exactly that combination of interests than **Hifi only**.
- **Check boxes to be ticked:** Games and iPhone, Food and Travel
- **Task Type:** Intersection comparison - More than.
- **SNAP Data Set Used:** 629863
- ED-N

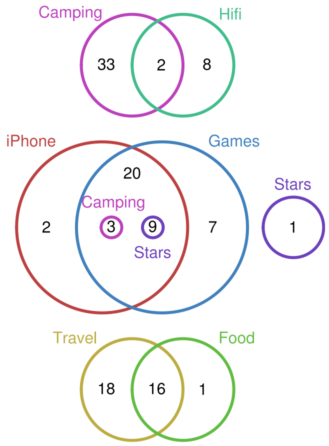

- ED-P

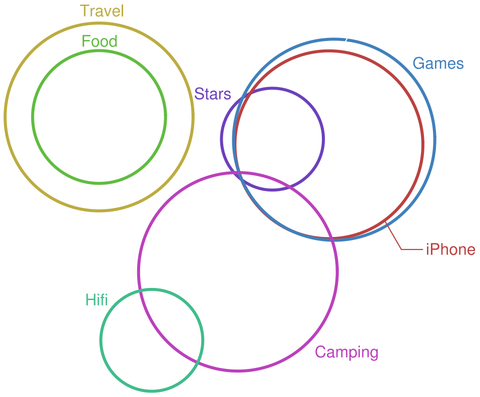

- ED-P&N

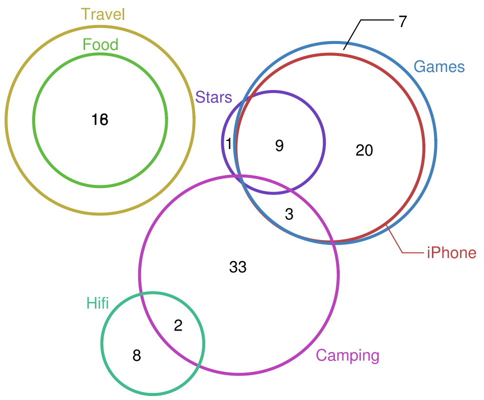
Travel
Food
18
Stars
1
9
20
7
Games
3
iPhone
33
Hifi
2
8
Camping

- LD-N

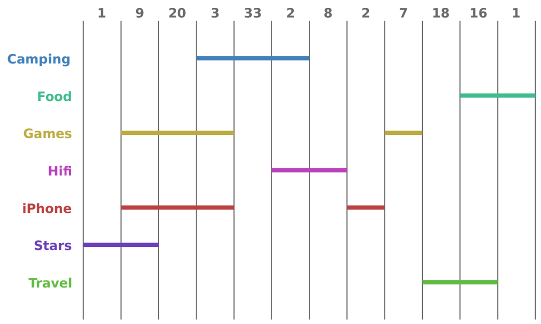
1 9 20 3 33 2 8 2 7 18 16 1
Camping
Food
Games
Hifi
iPhone
Stars
Travel

- LD-P

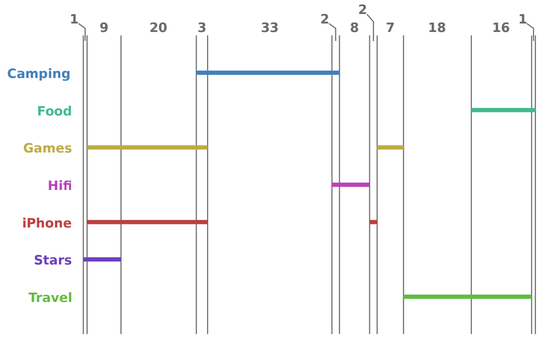
1 9 20 3 33 2 8 2 7 18 16 1
Camping
Food
Games
Hifi
iPhone
Stars
Travel

- LD-P&N

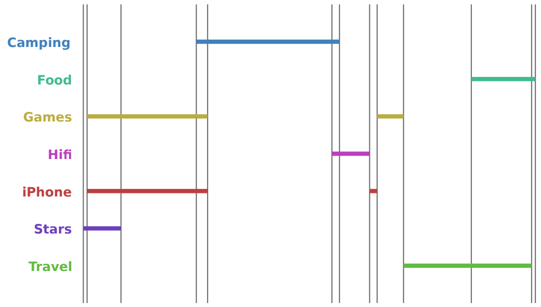
Camping
Food
Games
Hifi
iPhone
Stars
Travel

Question 28

- **Question:** Tick the check boxes where **more people** have exactly that combination of interests than **Food only**.
- **Check boxes to be ticked:** None of the above
- **Task Type:** Intersection comparison - Less than.
- **SNAP Data Set Used:** 30031265
- ED-N

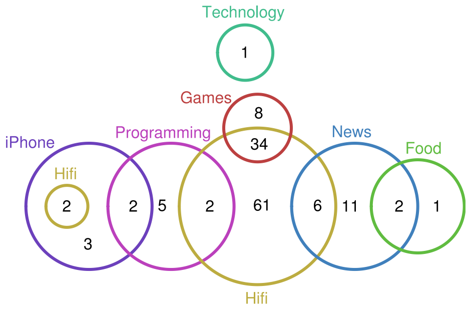

- ED-P

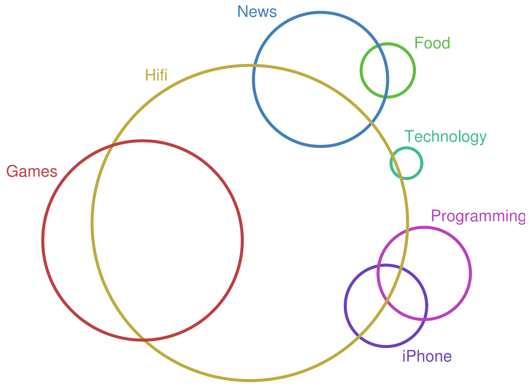

- ED-P&N

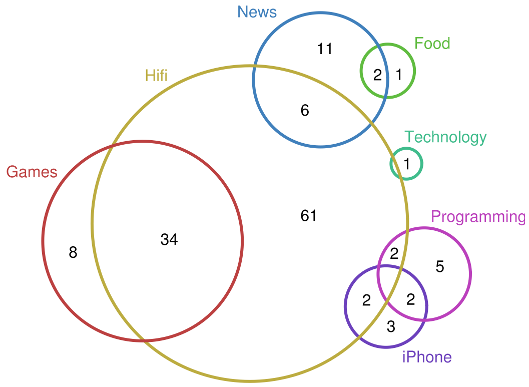

- LD-N

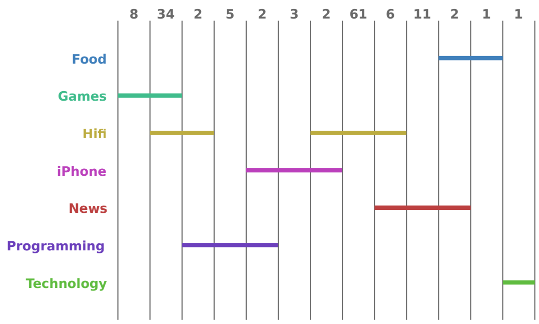
8
34
2
5
2
3
2
61
6
11
2
1
1
Food
Games
Hifi
iPhone
News
Programming
Technology

- LD-P

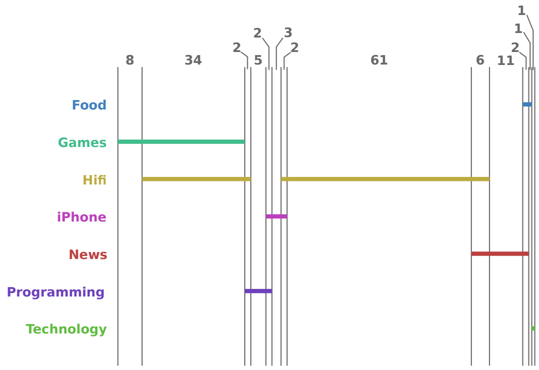
1
1
2
3
2
2
2
8
34
5
61
6
11
Food
Games
Hifi
iPhone
News
Programming
Technology

- LD-P&N

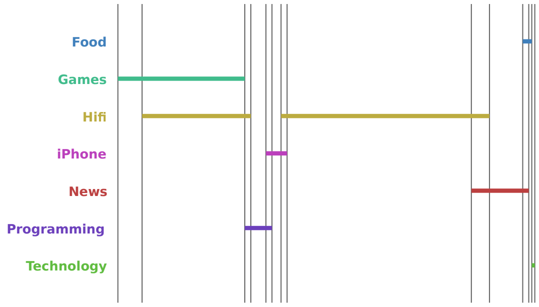
Food
Games
Hifi
iPhone
News
Programming
Technology

Question 29

- **Question:** Tick the check boxes where the total number of people interested in that topic is **greater than** the total number of people interested in **Web**.
- **Check boxes to be ticked:** Design, Health, Stars
- **Task Type:** Set comparison - More than.
- **SNAP Data Set Used:** 20436059
- ED-N

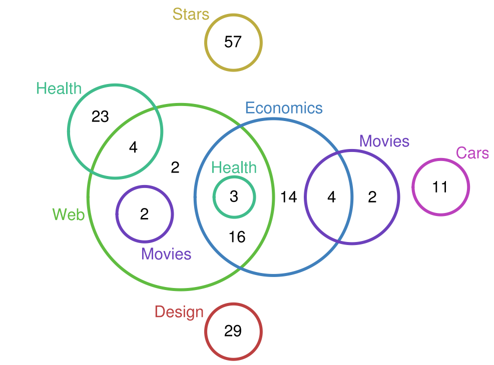

- ED-P

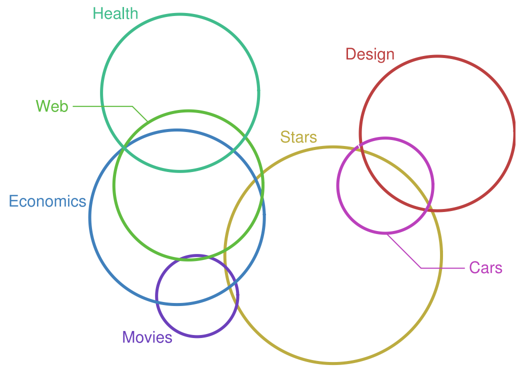

- ED-P&N

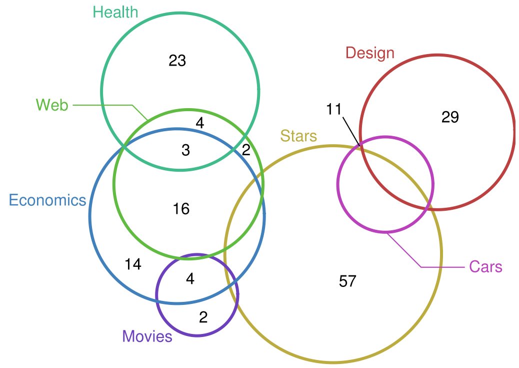

- LD-N

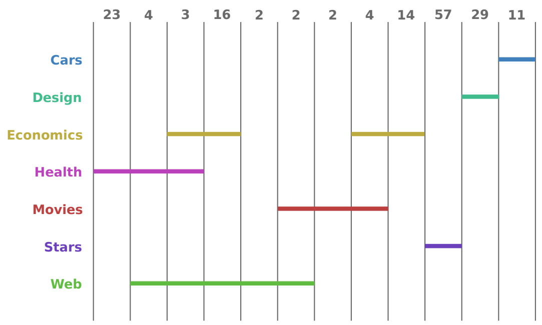
23 4 3 16 2 2 2 4 14 57 29 11
Cars
Design
Economics
Health
Movies
Stars
Web

- LD-P

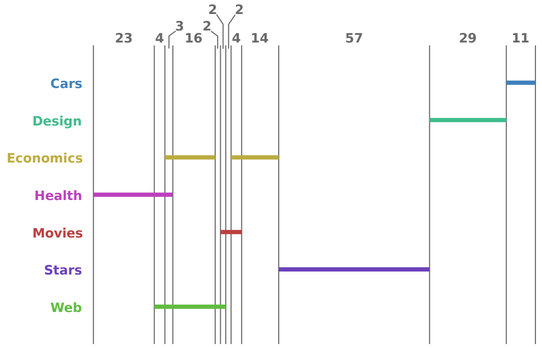
2 2
3 2
23 4 16 4 14 57 29 11
Cars
Design
Economics
Health
Movies
Stars
Web

- LD-P&N

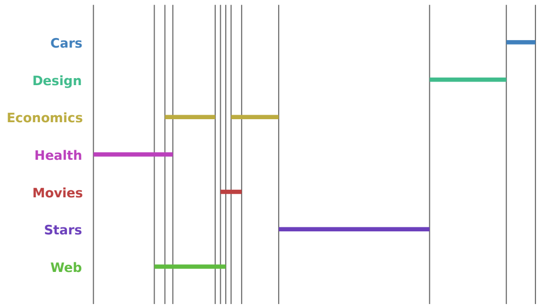
Cars
Design
Economics
Health
Movies
Stars
Web

Question 30

- **Question:** Tick the check boxes where the total number of people interested in that topic is **less than** the total number of people interested in **Economics**.
- **Check boxes to be ticked:** College, Programming
- **Task Type:** Set comparison - Less than.
- **SNAP Data Set Used:** 30207757
- ED-N

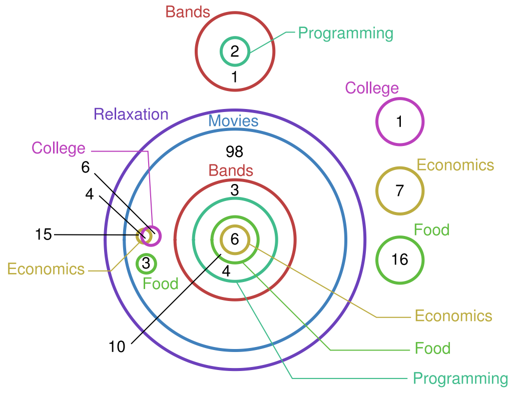

- ED-P

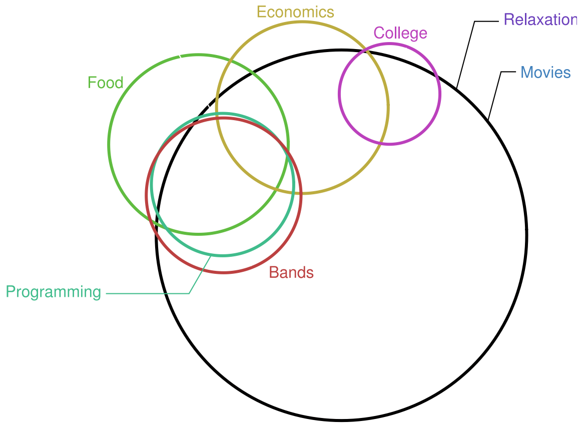

- ED-P&N

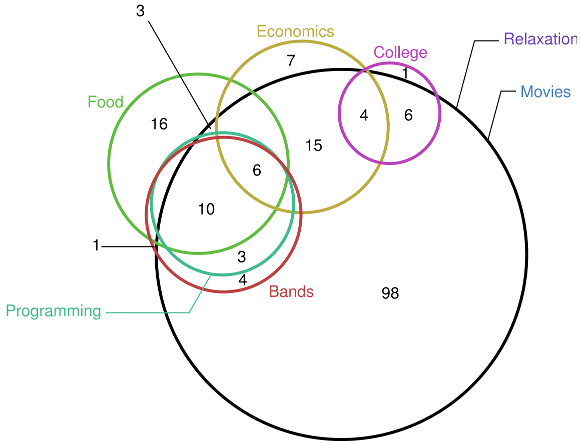
Economics
College
Relaxation
Movies
Food
Programming
Bands
3
7
1
16
4
6
15
6
10
1
3
4
98

- LD-N

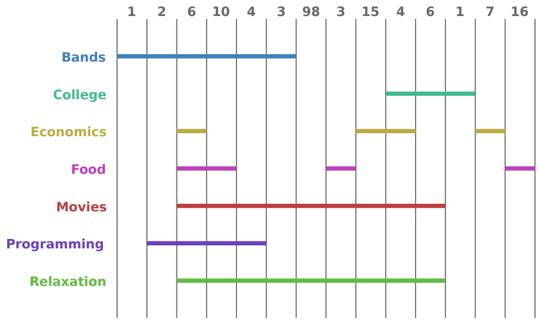
1 2 6 10 4 3 98 3 15 4 6 1 7 16
Bands
College
Economics
Food
Movies
Programming
Relaxation

- LD-P

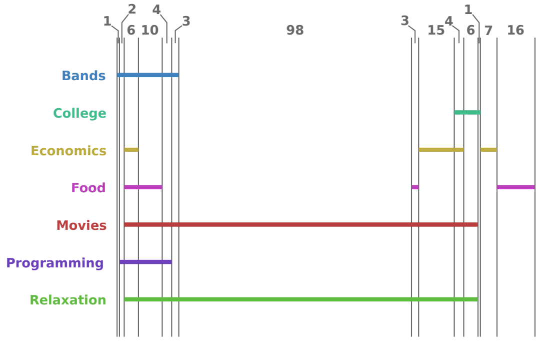
1 2 6 10 4 3 98 3 15 4 1 6 7 16
Bands
College
Economics
Food
Movies
Programming
Relaxation

- LD-P&N

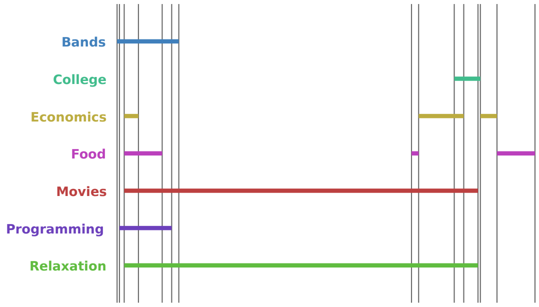
Bands
College
Economics
Food
Movies
Programming
Relaxation

Question 31

- **Question:** Tick the check boxes where **more people** have exactly that combination of interests than **Stars only**.
- **Check boxes to be ticked:** None of the above
- **Task Type:** Intersection comparison - More than.
- **SNAP Data Set Used:** 14060856
- ED-N

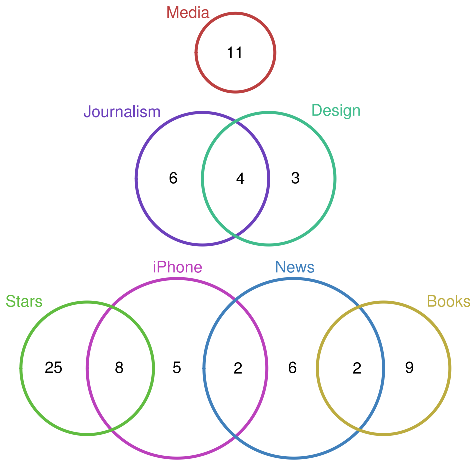

- ED-P

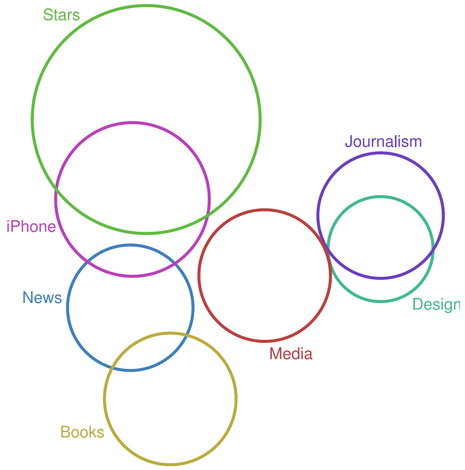

- ED-P&N

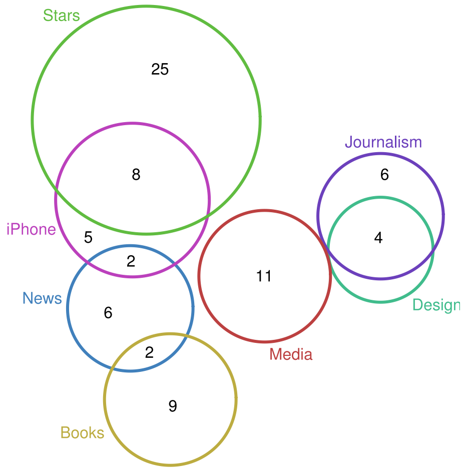
Stars
25
8
iPhone
5
2
News
6
2
9
Books
11
Media
Journalism
6
4
Design

- LD-N

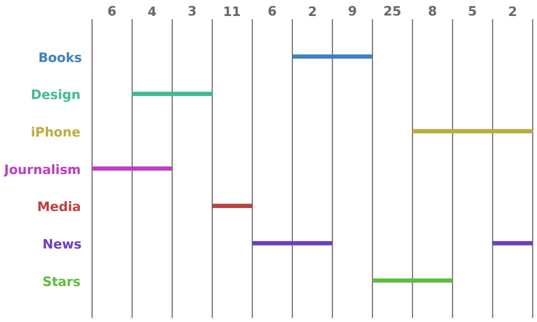
6 4 3 11 6 2 9 25 8 5 2
Books
Design
iPhone
Journalism
Media
News
Stars

- LD-P

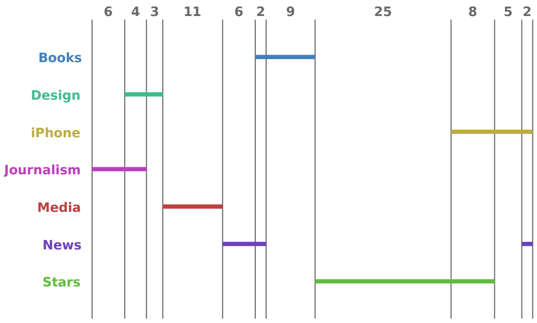
6 4 3 11 6 2 9 25 8 5 2
Books
Design
iPhone
Journalism
Media
News
Stars

- LD-P&N

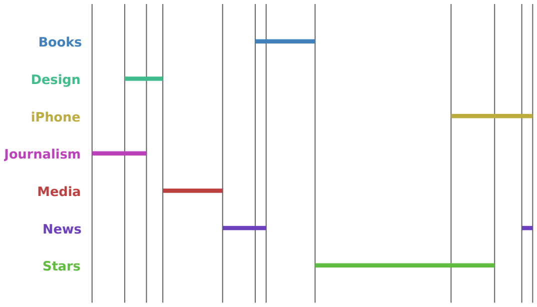
Books
Design
iPhone
Journalism
Media
News
Stars

Question 32

- **Question:** Tick the check boxes where **more people** have exactly that combination of interests than **Games, Internet and Technology only**.
- **Check boxes to be ticked:** None of the above
- **Task Type:** Intersection comparison - Less than.
- **SNAP Data Set Used:** 13274152
- ED-N

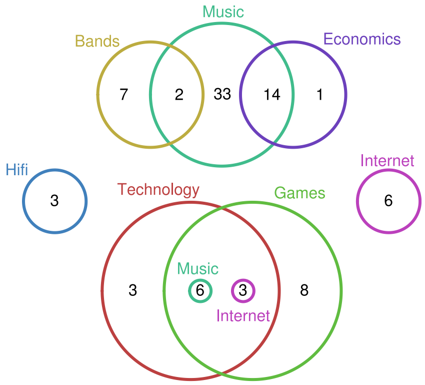

- ED-P

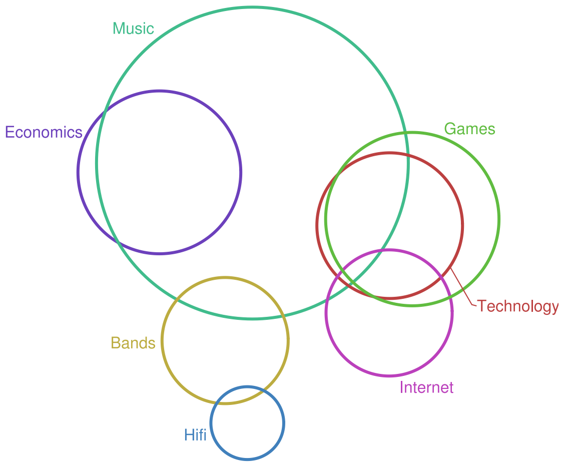

- ED-P&N

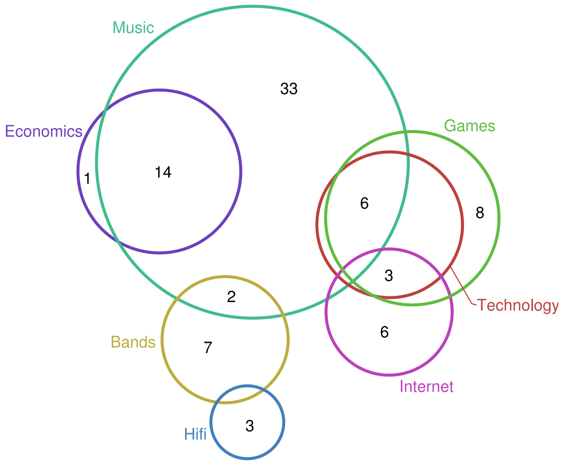
Music
33
Economics
1
14
Games
6
8
3
Technology
2
Bands
7
6
Internet
Hifi
3

- LD-N

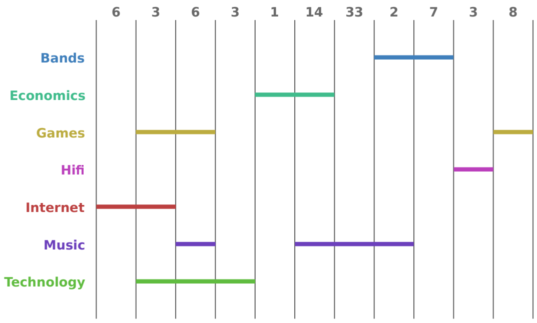
6 3 6 3 1 14 33 2 7 3 8
Bands
Economics
Games
Hifi
Internet
Music
Technology

- LD-P

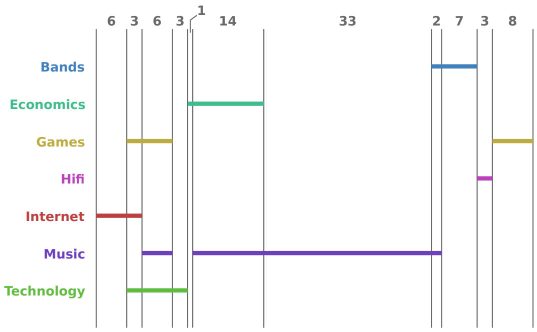
1
6 3 6 3 14 33 2 7 3 8
Bands
Economics
Games
Hifi
Internet
Music
Technology

- LD-P&N

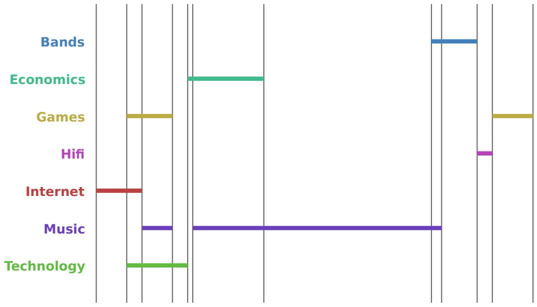
Bands
Economics
Games
Hifi
Internet
Music
Technology

D - Statistical Methods

Here we present in detail the statistical models used for the first two studies, each of which included three treatment groups. The models used for the last study were simpler, as there were only two treatments.

For each of the three empirical studies, we employed two local odds ratios generalized estimating equations models [2] to analyse the accuracy data. An ANOVA calculation was not appropriate for these data as they violated the normality assumption of an ANOVA test. The non-parametric version of ANOVA, Kruskal-Wallis, was also not appropriate, as the responses for each individual are correlated, and thus not independent. The first model, for the first two studies, was employed to answer RQ1, RQ2 and RQ3, compared the visualization types overall irrespective of task category:

$$\log\left[\frac{\Pr(Y_{ik} \leq j)}{1 - \Pr(Y_{ik} \leq j)}\right] \;=\; \beta_{0j} + \beta_1 x_{ik1} + \beta_2 x_{ik2}$$

where

- $\Pr(Y_{ik} \leq j)$ is the probability for subject i to provide at most $j-1$ correct answers to question k and where $j = 1, ..., 5$[1].
- x_{ik1} is the indicator that the diagram given to subject i for answering question k was *proportional*, and
- x_{ik2} is the indicator that the diagram given to subject i for answering question k was *both proportional and numerical*

for $i = 1, \ldots, n$, given n participants, and $k = 1, \ldots, 32$. With this model, we could determine whether the odds of providing j or fewer correct answers for one of the visualization types (proportional, numerical, or both) was significantly different from others while taking into account the expected correlation among the responses provided by each individual participant.

The second model, for the first two studies, was employed to see whether the answers to RQ1, RQ2 and RQ3 still held when we take into account task category (set or intersection):

$$\begin{aligned}\log\left[\frac{\Pr(Y_{ik} \leq j)}{1 - \Pr(Y_{ik} \leq j)}\right] \;&=\; \beta_{0j} + \beta_1 x_{ik1} + \beta_2 x_{ik2} + \beta_3 x_{ik3} + \\ &\quad\; \beta_4(x_{ik1} \times x_{it3}) + \beta_5(x_{ik2} \times x_{ik3})\end{aligned}$$

where the variables are as above and, in addition, x_{ik3} is the indicator that the diagram given to subject i for answering question k was of type *I*. This model allowed us to estimate the odds of providing j or fewer correct answers with one combination of visualization type and task category compared to other combinations and determine whether significant differences existed.

For the time data, we used two generalized estimation models [1] that allowed us to estimate whether the time taken to provide answers was significantly different. Again we present details of the two models used for the first two studies. Following a similar approach for the accuracy data, the first of the two models directly addressed RQ1, RQ2 and RQ3 and the more complex model delved deeper into the data to see whether task category was important. The more complex model is given here, which

[1] j takes values from 1 to 5 since, for each question, there are 5 checkboxes which need to be correctly checked or not.

accounts for the different combinations of visualization type (proportional, numerical, or both) and task category (set or intersection):

$$\begin{aligned}\log(t_{ik}) &= \delta_0 + \delta_1 x_{ik1} + \delta_2 x_{ik2} + \delta_3 x_{ik3} + \\ &\quad \delta_4(x_{ik1} \times x_{ik3}) + \delta_5(x_{ik2} \times x_{ik3})\end{aligned}$$

where

- t_{ik} is the time needed for subject i to answer question k, and
- x_{ik1}, x_{ik2} and x_{it3} are defined in the previous model

for $i = 1, \ldots, n$, given n participants, and $k = 1, \ldots, 32$. This model allowed us to estimate the ratio of times of answering a question, given a pair of combinations of visualization type and task category. The simpler model excluded all terms involving x_{ik3}.

Statistical output is included in the submitted supplementary material. We report on the main findings in the associated paper.

E - Study A (ED) - Statistical Analysis

Here we present the statistical models used specifically for study 1 and the associated output.

Models

For the overall comparison, the following model was fitted to the accuracy data

$$\log\left[\frac{\Pr(Y_{ik} \leq j)}{1 - \Pr(Y_{ik} \leq j)}\right] = \beta_{0j} + \beta_1 x_{ik1} + \beta_2 x_{ik2}$$

where

- $\Pr(Y_{ik} \leq j)$ is the probability for participant i to provide at most $j - 1$ correct answers to question k and where $j = 1, 2, 3, 4, 5$.
- x_{ik1} is the indicator that the diagram given to participant i for answering question k was *ED-P*,
- x_{ik2} is the indicator that the diagram given to participant i for answering question k was *ED-P&N*

for $i = 1, \ldots, 277$ and $j = 1, \ldots, 32$.

For the overall comparison, the following model was fitted to the time data

$$\log(t_{ik}) = \delta_0 + \delta_1 x_{ik1} + \delta_2 x_{ik2}$$

where

- t_{ik} is the time needed for participant i to answer question k,
- x_{ik1} and x_{ik2} are defined in the previous model

for $i = 1, \ldots, 277$ and $k = 1, \ldots, 32$.

For the set and intersection comparisons, following model was fitted to the accuracy data

$$\log\left[\frac{\Pr(Y_{ik} \leq j)}{1-\Pr(Y_{ik} \leq j)}\right] = \beta_{0j}+\beta_1 x_{ik1}+\beta_2 x_{ik2}+\beta_3 x_{ik3}+\beta_4(x_{ik1}\times x_{ik3})+\beta_5(x_{ik2}\times x_{ik3})$$

where

- $\Pr(Y_{ik} \leq j)$ is the probability for participant i to provide at most $j-1$ correct answers to question k and where $j = 1, 2, 3, 4, 5$.
- x_{ik1} is the indicator that the diagram given to participant i for answering question k was *ED-P*,
- x_{ik2} is the indicator that the diagram given to participant i for answering question k was *ED-P&N*
- x_{ik3} is the indicator that the diagram given to participant i for answering question k was of type I

for $i = 1, \ldots, 277$ and $j = 1, \ldots, 32$.

For the set and intersection comparisons, the following model was fitted to the time data

$$\log(t_{ik}) = \delta_0 + \delta_1 x_{ik1} + \delta_2 x_{ik2} + \delta_3 x_{ik3} + \delta_4(x_{ik1}\times x_{ik3}) + \delta_5(x_{ik2}\times x_{ik3})$$

where

- t_{ik} is the time needed for participant i to answer question k,
- x_{ik1}, x_{ik2} and x_{ik3} are defined in the previous model

for $i = 1, \ldots, 277$ and $k = 1, \ldots, 32$.

Overall results

The accuracy analysis:

```
# ED-P vs ED-N
ComparisonStats(fitmodel,c(0, 0, 0, 0, 0, 1, 0))
```

```
## Estimate   95% LB   95% UB  p-value
## 1.315777 1.058239 1.635991 0.013500
```

```
# ED-P&N vs ED-N
ComparisonStats(fitmodel,c(0, 0, 0, 0, 0, 0, 1))
```

```
##  Estimate    95% LB    95% UB   p-value
## 0.8866999 0.6888363 1.1413985 0.3506000
```

```
# ED-P vs ED-P&N
ComparisonStats(fitmodel,c(0, 0, 0, 0, 0, 1, -1))
```

```
## Estimate   95% LB   95% UB  p-value
## 1.483904 1.183448 1.860640 0.000600
```

The inference will be based on the model based estimated odds ratios (the value under Estimate). The results will be declared statistically significant only if the corresponding p-value is less than 0.05 and/or if the corresponfing 95% confidence interval does not contain 1. The two middle columns correspond to the lower and the upper bound of the 95% confidence interval for the odds ratios. The interpretation of

the above analysis is the following: The estimated odds of having j or less correct answers with *ED-P* diagrams is 1.3158 times that with *ED-N* diagrams (p-value $= 0.0135$). The estimated odds of having j or less correct answers with *ED-P* diagrams is 1.4839 times that with *ED-P&N* diagrams (p-value $= 0.0006$). In other words, *ED-N* and *ED-P&N* diagrams are more likely to produce a higher ammount of correct answers than *ED-P* diagrams.

The fitted probabilities for j for each group are

```
X1 <- matrix(fitted(fitmodel)[c(1000,8000,3000),],3,6)
rownames(X1) <- dataset1$diagram[c(1000,8000,3000)]
colnames(X1) <- 0:5
X1
```

```
##                  0          1          2         3         4
## ED-P    0.02431216 0.03763469 0.07304745 0.1789546 0.1394637
## ED-P&N  0.01651485 0.02609171 0.05255504 0.1405389 0.1228714
## ED-N    0.01858585 0.02920467 0.05824127 0.1520142 0.1286269
##                5
## ED-P   0.5465874
## ED-P&N 0.6414281
## ED-N   0.6133271
```

The time analysis:

```
# ED-P vs ED-N
ComparisonStats(fittimemodel,c(0, 1, 0))
```

```
##  Estimate    95% LB    95% UB   p-value
## 0.7809330 0.7081761 0.8611648 0.0000000
```

```
# ED-P&N vs ED-N
ComparisonStats(fittimemodel,c(0, 0, 1))
```

```
##  Estimate    95% LB    95% UB   p-value
## 0.9125743 0.8188579 1.0170163 0.0980000
```

```
# ED-P vs ED-P&N
ComparisonStats(fittimemodel,c(0, 1, -1))
```

```
##  Estimate    95% LB    95% UB   p-value
## 0.8557473 0.7741852 0.9459021 0.0023000
```

The inference will be based on the model based estimated ratios (the value under Estimate). The results will be declared statistically significant only if the corresponding p-value is less than 0.05 and/or if the corresponfing 95% confidence interval does not contain 1. The p-values and the 95% confidence intervals suggest that participants needed less time with *ED-P* diagrams than with *ED-P&N* or *ED-N* diagrams and that there is no significance difference between *ED-P&N* and *ED-N* diagrams.

Set cardinalities results

Accuracy comparisons:

```
# (ED-P and type S) vs (ED-N and type S)
ComparisonStats(fitmodel,c(0, 0, 0, 0, 0, 1, 0, 0, 0, 0))
```

```
##  Estimate    95% LB    95% UB   p-value
## 0.8069548 0.6083496 1.0703979 0.1367000
```

```
# (ED-P&N and type S) vs (ED-N and type S)
ComparisonStats(fitmodel,c(0, 0, 0, 0, 0, 0, 1, 0, 0, 0))
```

```
##  Estimate    95% LB    95% UB   p-value
## 0.5059211 0.3544472 0.7221277 0.0002000
```

```
# (ED-P and type S) vs (ED-P&N and type S)
ComparisonStats(fitmodel,c(0, 0, 0, 0, 0, 1,-1, 0, 0, 0))
```

```
## Estimate   95% LB   95% UB  p-value
## 1.595021 1.094169 2.325136 0.015200
```

The inference will be based on the model based estimated odds ratios (the value under Estimate). The results will be declared statistically significant only if the corresponding p-value is less than 0.05 and/or if the corresponfing 95% confidence interval does not contain 1. The two middle columns correspond to the lower and the upper bound of the 95% confidence interval for the odds ratios. The interpretation of the above analysis is the following: For type S diagrams, the estimated odds of having j or less correct answers with *ED-P* diagrams is 0.8070 times that with *ED-N* diagrams (p-value = 0.1367). The estimated odds of having j or less correct answers with *ED-P* diagrams is 1.5950 times that with *ED-P&N* diagrams (p-value = 0.0152). In other words, for type S, *ED-N* and *ED-P&N* diagrams are more likely to produce a higher ammount of correct answers than *ED-P* diagrams and there is no difference between *ED-N* and *ED-P&N* diagrams.

Time comparisons:

```
# (ED-P and type S) vs (ED-N and type S)
ComparisonStats(fittimemodel,c(0, 1, 0, 0, 0, 0))
```

```
##  Estimate    95% LB    95% UB   p-value
## 0.6839369 0.6223542 0.7516133 0.0000000
```

```
# (ED-P&N and type S) vs (ED-N and type S)
ComparisonStats(fittimemodel,c(0, 0, 1, 0, 0, 0))
```

```
##  Estimate    95% LB    95% UB   p-value
## 0.8750651 0.7878886 0.9718872 0.0127000
```

```
# (ED-P and type S) vs (ED-P&N and type S)
ComparisonStats(fittimemodel,c(0, 1,-1, 0, 0, 0))
```

```
##  Estimate    95% LB    95% UB   p-value
## 0.7815840 0.7073398 0.8636211 0.0000000
```

The inference will be based on the model based estimated ratios (the value under Estimate). The results will be declared statistically significant only if the corresponding p-value is less than 0.05 and/or if the corresponfing 95% confidence interval does not contain 1. For type I, the p-values and the 95% confidence intervals suggest that participants needed less time with *ED-P* diagrams than with *ED-P&N* or *ED-N* diagrams, and that participants needed less time with *ED-P&N* than *ED-N* diagrams.

Intersection cardinalities results

Accuracy analysis:

```
# (ED-P and type I) vs (ED-N and type I)
ComparisonStats(fitmodel,c(0, 0, 0, 0, 0, 1, 0, 0, 1, 0))
```

```
## Estimate   95% LB   95% UB  p-value
## 1.862365 1.445066 2.400170 0.000000
```

```
# (ED-P&N and type I) vs (ED-N and type I)
ComparisonStats(fitmodel,c(0, 0, 0, 0, 0, 0, 1, 0, 0, 1))
```

```
## Estimate   95% LB   95% UB  p-value
## 1.266412 0.944948 1.697235 0.113900
```

```
# (ED-P and type I) vs (ED-P&N and type I)
ComparisonStats(fitmodel,c(0, 0, 0, 0, 0, 1,-1, 0, 1,-1))
```

Time analysis:

```
# (ED-P and type I) vs (ED-N and type I)
ComparisonStats(fittimemodel,c(0, 1, 0, 0, 1, 0))
```

```
##  Estimate    95% LB    95% UB   p-value
## 0.8916851 0.7972190 0.9973450 0.0448000
```

```
# (ED-P&N and type I) vs (ED-N and type I)
ComparisonStats(fittimemodel,c(0, 0, 1, 0, 0, 1))
```

```
##  Estimate    95% LB    95% UB   p-value
## 0.9516914 0.8449521 1.0719146 0.4146000
```

```
# (ED-P and type I) vs (ED-P&N and type I)
ComparisonStats(fittimemodel,c(0, 1,-1, 0, 1,-1))
```

```
##  Estimate    95% LB    95% UB   p-value
## 0.9369478 0.8377156 1.0479345 0.2542000
```

F - Study B (LD) - Statistical models and output

Here we present the statistical models used specifically for study 2 and the associated output.

Models

For the overall comparison, the following model was fitted to the accuracy data

$$\log\left[\frac{\Pr(Y_{ik} \leq j)}{1-\Pr(Y_{ik} \leq j)}\right] = \beta_{0j} + \beta_1 x_{ik1} + \beta_2 x_{ik2}$$

where

- $\Pr(Y_{ik} \leq j)$ is the probability for participant i to provide at most $j-1$ correct answers to question k and where $j = 1, 2, 3, 4, 5$.

- x_{ik1} is the indicator that the diagram given to participant i for answering question k was *LD-P*,
- x_{ik2} is the indicator that the diagram given to participant i for answering question k was *LD-P&N*

for $i = 1, \ldots, 272$ and $k = 1, \ldots, 32$.

For the overall comparison, the following model was fitted to the time data

$$\log(t_{ik}) = \delta_0 + \delta_1 x_{ik1} + \delta_2 x_{ik2}$$

where

- t_{ik} is the time needed for participant i to answer question k,
- x_{ik1} and x_{ik2} are defined in the previous model

for $i = 1, \ldots, 272$ and $k = 1, \ldots, 32$.

For the set and intersection comparisons, the following model was fitted to the accuracy data

$$\log\left[\frac{\Pr(Y_{ik} \leq j)}{1 - \Pr(Y_{ik} \leq j)}\right] = \beta_{0j} + \beta_1 x_{ik1} + \beta_2 x_{ik2} + \beta_3 x_{ik3} + \beta_4(x_{ik1} \times x_{ik3}) + \beta_5(x_{ik2} \times x_{ik3})$$

where

- $\Pr(Y_{ik} \leq j)$ is the probability for participant i to provide at most $j-1$ correct answers to question k and where $j = 1, 2, 3, 4, 5$.
- x_{ik1} is the indicator that the diagram given to participant i for answering question k was *LD-P*,
- x_{ik2} is the indicator that the diagram given to participant i for answering question k was *LD-P&N*
- x_{ik3} is the indicator that the diagram given to participant i for answering question k was of type I

for $i = 1, \ldots, 272$ and $k = 1, \ldots, 32$.

For the set and intersection comparisons, the following model was fitted to the time data

$$\log(t_{ik}) = \delta_0 + \delta_1 x_{ik1} + \delta_2 x_{ik2} + \delta_3 x_{ik3} + \delta_4(x_{ik1} \times x_{ik3}) + \delta_5(x_{ik2} \times x_{ik3})$$

where

- t_{ik} is the time needed for participant i to answer question k,
- x_{ik1}, x_{ik2} and x_{ik3} are defined in the previous model

for $i = 1, \ldots, 272$ and $k = 1, \ldots, 32$.

Overall results

Accuracy analysis:

```
# LD-P vs LD-N
ComparisonStats(fitmodel,c(0, 0, 0, 0, 0, 1, 0))
```

```
##  Estimate    95% LB    95% UB   p-value
## 1.1348457 0.9399509 1.3701512 0.1882000
```

```r
# LD-P&N vs LD-N
ComparisonStats(fitmodel,c(0, 0, 0, 0, 0, 0, 1))
```

```
##  Estimate    95% LB    95% UB   p-value
## 1.1018091 0.8897432 1.3644198 0.3741000
```

```r
# LD-P vs LD-P&N
ComparisonStats(fitmodel,c(0, 0, 0, 0, 0, 1, -1))
```

```
##  Estimate    95% LB    95% UB   p-value
## 1.0299840 0.8456953 1.2544318 0.7690000
```

There is no difference between the three types of diagrams.

Time analysis:

```r
# LD-P vs LD-N
ComparisonStats(fittimemodel,c(0, 1, 0))
```

```
##  Estimate    95% LB    95% UB   p-value
## 0.8134194 0.7435824 0.8898155 0.0000000
```

```r
# LD-P&N vs LD-N
ComparisonStats(fittimemodel,c(0, 0, 1))
```

```
##  Estimate    95% LB    95% UB   p-value
## 0.9797777 0.8980481 1.0689453 0.6457000
```

```r
# LD-P vs LD-P&N
ComparisonStats(fittimemodel,c(0, 1, -1))
```

```
##  Estimate    95% LB    95% UB   p-value
## 0.8302082 0.7636182 0.9026049 0.0000000
```

Participants needed less time with *LD-P* diagrams than with *LD-P&N* or *LD-N* diagrams and that there is no significance difference between *LD-P&N* and *LD-N* diagrams.

Set cardinalities results

Accuracy analysis:

```r
# (LD-P and type S) vs (LD-N and type S)
ComparisonStats(fitmodel,c(0, 0, 0, 0, 0, 1, 0, 0, 0, 0))
```

```
##  Estimate    95% LB    95% UB   p-value
## 1.0263526 0.7901575 1.3331515 0.8454000
```

```r
# (LD-P&N and type S) vs (LD-N and type S)
ComparisonStats(fitmodel,c(0, 0, 0, 0, 0, 0, 1, 0, 0, 0))
```

```
##  Estimate    95% LB    95% UB   p-value
## 1.2253349 0.9403471 1.5966931 0.1324000
```

```r
# (LD-P and type S) vs (LD-P&N and type S)
ComparisonStats(fitmodel,c(0, 0, 0, 0, 0, 1,-1, 0, 0, 0))
```

```
##  Estimate    95% LB    95% UB   p-value
```

```
## 0.8376098 0.6365894 1.1021079 0.2057000
```

For type *S*, there is no difference among the three types of diagrams.

Time analysis:

```r
# (LD-P and type S) vs (LD-N and type S)
ComparisonStats(fittimemodel,c(0, 1, 0, 0, 0, 0))
```

```
##  Estimate    95% LB    95% UB   p-value
## 0.7846409 0.7208330 0.8540971 0.0000000
```

```r
# (LD-P&N and type S) vs (LD-N and type S)
ComparisonStats(fittimemodel,c(0, 0, 1, 0, 0, 0))
```

```
##  Estimate    95% LB    95% UB   p-value
## 0.9970524 0.9150658 1.0863846 0.9462000
```

```r
# (LD-P and type S) vs (LD-P&N and type S)
ComparisonStats(fittimemodel,c(0, 1,-1, 0, 0, 0))
```

```
##  Estimate    95% LB    95% UB   p-value
## 0.7869606 0.7257799 0.8532986 0.0000000
```

For type *S*, participants needed less time with *LD-P* diagrams than with *LD-P&N* or *LD-N* and there is no significance difference between *LD-P&N* and *LD-N* diagrams.

Intersection cardinalities results

Accuracy analysis:

```r
# (LD-P and type I) vs (LD-N and type I)
ComparisonStats(fitmodel,c(0, 0, 0, 0, 0, 1, 0, 0, 1, 0))
```

```
##  Estimate    95% LB    95% UB   p-value
## 1.2038819 0.9449141 1.5338235 0.1332000
```

```r
# (LD-P&N and type I) vs (LD-N and type I)
ComparisonStats(fitmodel,c(0, 0, 0, 0, 0, 0, 1, 0, 0, 1))
```

```
##  Estimate    95% LB    95% UB   p-value
## 1.0341693 0.7835026 1.3650321 0.8125000
```

```r
# (LD-P and type I) vs (LD-P&N and type I)
ComparisonStats(fitmodel,c(0, 0, 0, 0, 0, 1,-1, 0, 1,-1))
```

```
##  Estimate    95% LB    95% UB   p-value
## 1.1641052 0.9025326 1.5014869 0.2419000
```

Time analysis:

```r
# (LD-P and type Z) vs (LD-N and type Z)
ComparisonStats(fittimemodel,c(0, 1, 0, 0, 1, 0))
```

```
##  Estimate    95% LB    95% UB   p-value
## 0.8432534 0.7600814 0.9355265 0.0013000
```

```r
# (LD-P&N and type Z) vs (LD-N and type Z)
ComparisonStats(fittimemodel,c(0, 0, 1, 0, 0, 1))
```

```
##  Estimate    95% LB    95% UB   p-value
## 0.9628023 0.8730566 1.0617733 0.4477000
```

```r
# (LD-P and type Z) vs (LD-P&N and type Z)
ComparisonStats(fittimemodel,c(0, 1,-1, 0, 1,-1))
```

```
##  Estimate    95% LB    95% UB   p-value
## 0.8758324 0.7958562 0.9638455 0.0067000
```

G - Study C (ED vs LD) - Statistical models and output

Here we present the statistical models used specifically for study 3 and the associated output.

Models

For the overall comparison, the following regression model was fitted to the accuracy data

$$\log\left[\frac{\Pr(Y_{ik} \leq j)}{1-\Pr(Y_{ik} \leq j)}\right] = \beta_{0j} + \beta_1 x_{ik1}$$

where

- $\Pr(Y_{ik} \leq j)$ is the probability for participant i to provide at most $j-1$ correct answers to question k and where $j = 1, 2, 3, 4, 5$.
- x_{ik1} is the indicator that an *ED-P&N* diagram was given to participant i for answering question k,

for $i = 1, \ldots, 185$ and $k = 1, \ldots, 32$.

For the overall comparison, the following regression model was fitted to the time data

$$\log\left(t_{ik}\right) = \delta_0 + \delta_1 x_{ik1}$$

where

- t_{ik} is the time needed for participant i to answer question t,
- x_{ik1} is defined as in the model for the accuracy data

for $i = 1, \ldots, 185$ and $k = 1, \ldots, 32$.

For the set and intersection comparisons, the following regression model was fitted to the accuracy data

$$\log\left[\frac{\Pr(Y_{ik} \leq j)}{1-\Pr(Y_{ik} \leq j)}\right] = \beta_{0j} + \beta_1 x_{ik1} + \beta_2 x_{ik2} + \beta_3 (x_{ik1} \times x_{ik2})$$

where

- $\Pr(Y_{ik} \leq j)$ is the probability for participant i to provide at most $j-1$ correct answers to question k and where $j = 1, 2, 3, 4, 5$.
- x_{ik1} is the indicator that an *ED-P&N* diagram was given to participant i for answering question k,
- x_{ik2} is the indicator that the k-th question given to participant i was type I

for $i = 1, \ldots, 185$ and $k = 1, \ldots, 32$.

For the set and intersection comparisons, the following regression model was fitted to the time data

$$\log(t_{ik}) = \delta_0 + \delta_1 x_{ik1} + \delta_2 x_{ik2} + \delta_3 (x_{ik1} \times x_{ik2})$$

where

- t_{ik} is the time needed for participant i to answer question k,
- x_{ik1} and x_{ik2} are defined as in the model for the accuracy data

for $i = 1, \ldots, 185$ and $k = 1, \ldots, 32$.

Overall results

Accuracy analysis:

```
# (ED-P&N) vs (LD-P)
ComparisonStats(fitmodel, c(0, 0, 0, 0, 0, 1))
```

```
##  Estimate    95% LB    95% UB   p-value
## 0.7814704 0.6249702 0.9771602 0.0306000
```

The *ED-P&N* diagrams are better than *LD-P* when the questions type is ignored.

Time analysis:

```
# (ED-P&N) vs (LD-P)
ComparisonStats(fittimemodel, c(0, 1))
```

```
## Estimate   95% LB   95% UB  p-value
## 1.159591 1.037295 1.296306 0.009200
```

The time needed with a *ED-P&N* diagram is 1.15 times that needed with a *LD-P* diagram when the question type was ignored.

Set cardinalities results

Accuracy analysis:

```
# (ED-P&N and typeS) vs (LD-P and typeS)
ComparisonStats(fitmodel, c(0, 0, 0, 0, 0, 1, 0, 0))
```

```
##  Estimate    95% LB    95% UB   p-value
## 0.9628247 0.6884480 1.3465527 0.8248000
```

There is no difference between *ED-P&N* and *LD-P* diagrams with for questions of type *S*.

Time analysis:

```
# (ED-P&N and typeS) vs (LD-P and typeS)
ComparisonStats(fittimemodel, c(0, 1, 0, 0))
```

```
## Estimate   95% LB   95% UB  p-value
## 1.255564 1.130982 1.393870 0.000000
```

To answer questions of type *S*, the time needed with a *ED-P&N* diagram is 1.26 times that needed with a *LD-P* diagram.

Intersection cardinalities results

Accuracy analysis:

```
# (ED-P&N and typeI) vs (LD-P and typeI)
ComparisonStats(fitmodel, c(0, 0, 0, 0, 0, 1, 0, 1))
```

```
##  Estimate    95% LB    95% UB   p-value
## 0.6571699 0.5113266 0.8446114 0.0010000
```

For questions of type *I*, *ED-P&N* diagrams are more likely to increase the number of correct answers when comprared to *LD-P* diagrams.

Time analysis:

```
# (ED-P&N and typeI) vs (LD-P and typeI)
ComparisonStats(fittimemodel, c(0, 1, 0, 1))
```

```
##  Estimate    95% LB    95% UB   p-value
## 1.0709541 0.9448343 1.2139088 0.2836000
```

References

1. K. Y. Liang and S. L. Zeger. Longitudinal data analysis using generalized linear models. *Biometrika*, 73:13–22, 1986.
2. A. Touloumis, A. Agresti, and M. Kateri. Generalized estimating equations for multinomial responses using a local odds ratios parameterization. *Biometrics*, 69(3):633–640, 2013.
